# Evaluation of mindfulness based stress reduction in symptomatic knee or hip osteoarthritis patients: a pilot randomized controlled trial

**DOI:** 10.1186/s41927-022-00277-9

**Published:** 2022-05-30

**Authors:** Clémentine Marais, Yujie Song, Rosanna Ferreira, Safa Aounti, Claire Duflos, Grégory Baptista, Yves-Marie Pers

**Affiliations:** 1 Inserm U1183, CHU Montpellier, IRMB, University of Montpellier, Montpellier, France; 2 Département Information Médicale, Hôpital La Colombière, CHU de Montpellier, Montpellier, France; 3 Centre de Mindfulness de Montpellier, Montpellier, France; 4 Service d’Immunologie Clinique et Thérapeutique ostéo-articulaire, Hôpital Lapeyronie, CHU Montpellier, 371, avenue du doyen Gaston Giraud, 34295 Montpellier, France

**Keywords:** Knee osteoarthritis, Hip osteoarthritis, Pain, Mindfulness, Randomized clinical trial

## Abstract

**Background:**

To evaluate the efficacy for symptomatic knee and hip osteoarthritis (OA) patients of a mindfulness-based stress reduction (MBSR) program versus usual care.

**Methods:**

Randomized, physician-blind, clinical trial in a monocentric prospective pilot study. Adult participants with symptomatic knee or hip OA were randomized into either intervention or control groups. The intervention group completed the MBSR program for a two-and-a-half-hour weekly session for 8 weeks. Usual care management was similar in both groups. All patients were evaluated at baseline, 3 months and 6 months. The primary objective was to evaluate the change in WOMAC pain score between baseline and 3 months in the MBSR group compared to usual care group. Secondary objectives were to evaluate changes in pain VAS, WOMAC scores, quality of life (SF-36), HAD scores between baseline and 3/6 months.

**Results:**

Forty patients were enrolled in the study. No differences in the WOMAC pain score between the two groups were observed in the different time points. A similar pattern was found for the other assessment outcomes. However, a significant pain VAS reduction in favor of the MBSR group between baseline and 6 months (− 29.6 ± 26.6 vs − 9.3 ± 27.3; *p* = 0.03) has been reached.

**Conclusions:**

Our pilot RCT found contrasting results with no benefit on WOMAC pain and function and a delayed but long-term efficacy in pain VAS following a MBSR program in symptomatic knee or hip OA patients. Future studies with larger sample size are mandatory to confirm these preliminary results.

*Trial registration* The study was registered in ClinicalTrials.gov (NCT03644615, 23/08/2018).

**Supplementary Information:**

The online version contains supplementary material available at 10.1186/s41927-022-00277-9.

## Introduction

Osteoarthritis (OA) is the most common and endemic rheumatism in the world, resulting in pain and deformation which can lead to loss of function. OA is the first cause of disability in the elderly, characterized by a major socio-economic cost [1, 2]. The pathophysiological process is complex related to mechanical, inflammatory and metabolic factors resulting in imbalance between anabolism and catabolism factors that affect articular cartilage but also the entire joint, including the synovium, articular ligaments and subchondral bone [3]. Currently, no treatment limiting the progression of the disease is available, thus, the physician aims to relieve pain and to restore function in order to minimize the impact of OA on quality of life.

Actually, OA patients are known to suffering of chronic nociceptive and neuropathic pain, induced by an association of peripheral and central mechanisms. The pain experience is modulated by many factors, including the individual patient’s psychological, socio economics context and genetic factors. Several studies of neuroimaging using functional MRI [4–7] have demonstrated that pain in knee and hip OA patients modified both the structure and function of the brain with abnormal activation of areas, especially the medial and prefrontal-limbic cortical areas, which are involved in emotional state, and recent studies which analyzed brain volume found some modifications in gray matter. That is particularly interesting because these alterations seem reversible in 6–9 months after effective hip or knee surgery with gray matter regeneration on MRI [8]. On the other hand, chronic pain seemed to reorganize the dynamics of the default mode network with decreased connectivity of medial prefrontal cortex, and increased connectivity to the insular cortex proportionally to the intensity of pain [9]. Finally, an increased supra-spinal facilitation of nociceptive signals in the dorsal horn and reduced descending pain inhibitory mechanisms have been found, which confirmed the central sensitization in knee and hip OA patients [10]. Regarding this concern, medications are useful. Notably local or systemic steroid and anti-inflammatory drugs are recommended during inflammatory flare, but not for chronic use because of their numerous cardiovascular, gastrointestinal and kidney sides effects. That’s why non-pharmacological interventions, such as active rehabilitation, weight reduction, and regular physical activity to stimulate cellular turnover and promote muscular strutting, are essential regardless of the medication used [11]. To date, studies have already demonstrated the efficacy of psychosocial, physical and mind–body interventions in reduction of pain in knee OA patients [12–14].

Although current OA medications using are active on peripheral nociceptive and neuropathic pain, these central modifications could still be a target. Mindfulness is a technique of attention training, which involves bringing attention back to the present moment and examining the sensations that arise in the mind. The "Mindfulness-Based Stress Reduction" (MBSR) cognitive therapy is a meditative exercise program aimed at reducing stress and eliminating anxiety states developed by Jon Kabat-Zinn [15, 16]. The MBSR program is an 8-week course that combines meditation to help patients cope with stress, pain, and disease using moment to moment awareness. MBSR programs help participants to find their inner resources for good health and well-being. A recent study published in 2015 demonstrated that mindfulness-based therapy is an effective alternative as treatment with antidepressants in the prevention of depressive relapses [17]. With regard to rheumatic diseases, MBSR program has shown its effectiveness in chronic pain [18]. In a recent meta-analysis, the authors compared cerebral MRI of adept of meditation with chronic pain patients and healthy individuals with experimental pain [9]. They founded that meditation was associated with an improvement in the affective experience of pain, while reductions of pain intensity were less consistent. Indeed, meditation led to deactivate the periaqueductal gray region, and the thalamus, which are a central node in opioid mediated descending inhibition, and a critical node of ascending nociceptive information from the spinal cord, respectively. Remarkably, all effects mentioned were proportional to meditation level of participants [19]. In musculoskeletal pain in particular, MBSR program had already shown efficacy in chronic low back pain [20] or in rheumatoid arthritis [21]. Regarding MBSR in OA, data are missing. A recent work observed that mindfulness exercises were significantly associated with a greater likelihood of response to non-pharmacological exercise interventions in knee OA [22]. In addition, the same group found a correlation between a predisposition to mindfulness and less pain and/or better quality of life in patients with knee OA [23]. Therefore, we conducted a randomized controlled trial (RCT) aiming to evaluate the efficacy on pain and function of a MBSR program, comparatively to its absence, in symptomatic knee and hip OA patients undergoing usual care during a 6-month follow-up.

## Patients and methods

### Study design

A prospective pilot controlled randomized monocentric study was performed, and patients aged 30 to 75 years with knee OA or hip OA were recruited into the Rheumatology Department at the Montpellier University Hospital, France. The measurements were conducted between September, 2018 and September 2019. Patients, diagnosed as having knee or hip OA according to the American College of Rheumatology clinical criteria [24], were eligible if they had reported joint pain for longer than 3 months, had radiological confirmation of OA (Kellgren-Lawrence score ≥ 2), had a pain score intensity > 4 out of 10 on the visual analogic scale (VAS) (0–10); and a stable dose of analgesics during the last week before inclusion. Exclusion criteria included the following: use of corticosteroid treatment in the last month, intra articular injection of steroid or hyaluronic acid within the 3 months, inflammatory rheumatic disease, depression, psychotic syndrome or other mental diseases, and the usual practice of relaxation technics.

The MINDFULNESS-OA study was approved by the French ethics committee for Health Research (CPP Ile de France III, June 2018) and by the national competent authority (ANSM). The study was registered in ClinicalTrials.gov (NCT03644615,23/08/2018). All the participant gave their written informed consent according to the Declaration of Helsinki prior to inclusion.

### Measurements and procedures

#### Randomization and blinding

Eligible participants were randomized to 2 groups by a central randomization system at a 1:1 ratio, using a random block sequence, and a stratification on the OA site (hip or knee).

#### Interventions

The MBSR program consisted of two-and-a-half-hour weekly sessions for 8 weeks, without the 7-h retreat, with a total of 20 h. It was delivered by an instructor (physician and psychotherapist) who trained at the Association pour le Développement de la Mindfulness (French representative of Mindfulness Center at Brown University, USA), attended silent retreats and have more than 3 years of teaching experience mindfulness-based interventions. MBSR program included training in mindfulness through (1) a body scan, the gradual moving of attention through the body from head to feet while lying on a mat on the floor, bringing awareness particularly to bodily sensations; (2) sitting meditation, in which attention is brought to breathing sensations and the flow of bodily sensations, sounds, thoughts, and emotions; and (3) mindful stretching exercises, to cultivate awareness during simple stretching movement. The program included 45-min daily homework exercises that consisted of guided (MP3 sent by email) or unguided awareness exercises directed at increasing moment-by-moment non-judgmental awareness of bodily sensations, thoughts and feelings, together with exercises designed to integrate the application of awareness skills into daily life. The key themes of MBSR included the empowerment of participants and a focus on awareness and acceptance of experience of the present moment [25]. The MBSR program did not focus specifically on a particular condition such as pain.

Usual care management was similar in both groups and included advice on the importance of weight loss, physical activity and self-management education in a booklet given to the patient. Management of pain and depressive symptoms was carried out according to standard practice.

#### Clinical evaluation

All patients were clinically evaluated at baseline and for each visit, by a blinded physician. Two following visits were scheduled at 3 months and 6 months. Measurements of weight, height, blood pressure, were collected for each subject as well as the examination of knees and hips.

Pain was assessed by VAS, functional impact was collected as well as analgesics consumption and eventual adverse events. The VAS used in this study was a 10-cm line ranging from 0 (no pain) to 10 (pain as bad as it could be) that assessed peak pain intensity over the last 24 h. Several questionnaires were also performed at each visit: Western Ontario and McMaster Universities Osteoarthritis Index (WOMAC), 36-Item Short-Form Health Survey (SF-36) [26], Five Facets Mindfulness Questionnaire (FFMQ) [27] and Hospital Anxiety and Depression (HAD) [28]. The WOMAC index [29] consists of three domains, namely pain (5 items), stiffness (2 items), and physical function (17 items), and each item is scored based on a 5-point Likert numerable rating scale representing different degrees of intensity (none, mild, moderate, severe, or extreme). The final score of WOMAC was determined by adding the aggregate scores for three subscales, which ranges from 0 to 100, and a greater score indicates greater pain and dysfunction.

#### Clinical assessment

The primary outcome was to evaluate the change in WOMAC pain score between baseline and 3 months in the MBSR group compared to usual care group. Secondary objectives were to evaluate changes in pain VAS, WOMAC scores (function, stiffness), quality of life (SF-36), HAD scores between baseline and 3 months, and between baseline and 6 months. We also performed the FFMQ questionnaire to find a predictive factor of patient response to the MBSR program.

#### Radiographic assessment

Bilateral knees postero-anterior (PA) radiographs in standing and pelvis position were performed at baseline for each patient to evaluate the Kellgren-Lawrence (K/L) score unless it had been carried out within 6 months.

#### Sample size

The current methods for setting pilot trial sample sizes are based on a set of rules, which is called flat rules of thumb. Browne recommended a general rule to use at least 30 subjects or greater to estimate a parameter [30], whereas Kieser and Wassmer suggested a pilot trial sample size between 20 and 40 [31]. Noteworthy, the simple size they mentioned was the total sample size required for a two-arm trial. Thus, in this pilot study, sample size was limited to 20 patients in each group, 40 in total, which was in accordance with the above criteria. However. no power calculation was done.

### Statistical analysis

Variables were described using mean and standard deviation (SD) for gaussian quantitative variables, median and quartiles for non-gaussian quantitative variables, and counts and percent for categorical variables. Variables were described in each group and compared using usual tests, after assessment of application conditions: t-test or Wilcoxon-Mann–Whitney test for quantitative variables, and Chi-square of Fisher’s exact test for qualitative variables.

## Results

### Patient characteristics

**Fig. 1:**
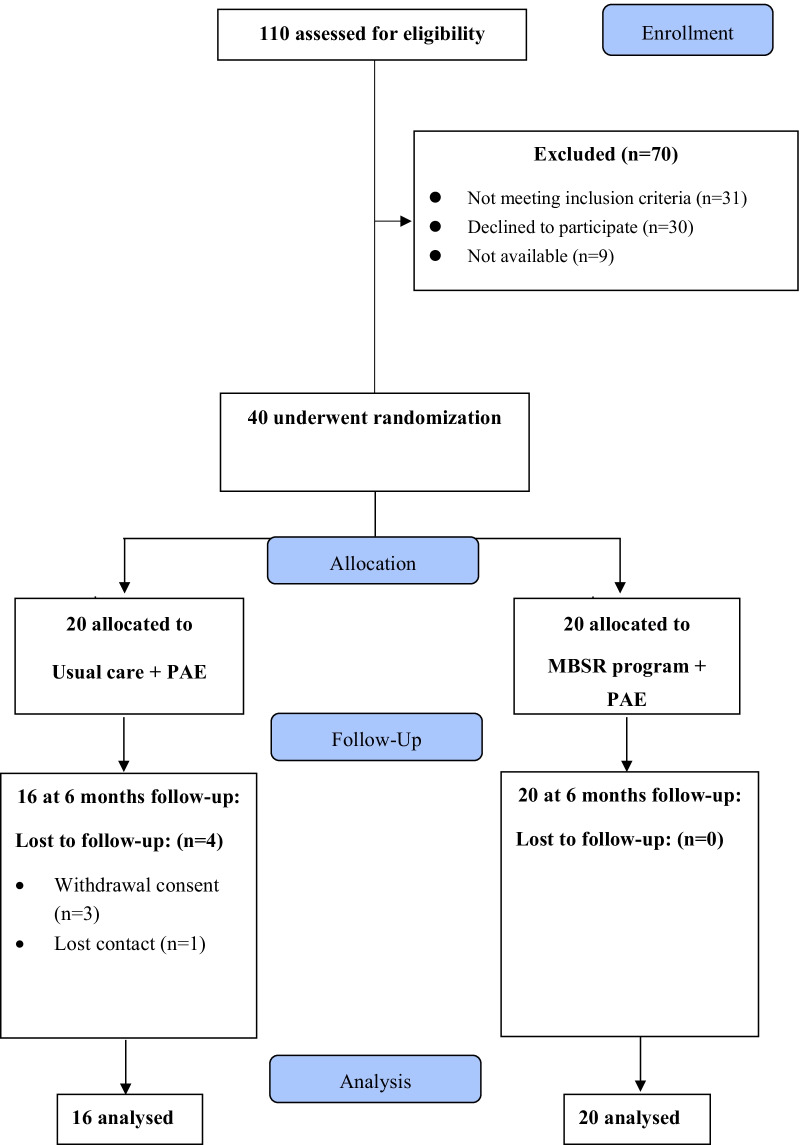
Flowchart of patients throughout the trial (PAE, physical activity exercise; MBSR, mindfulness-based stress reduction)

Forty patients were enrolled and randomly allocated in either MBSR group or the usual care group (Fig. 1). At the 6-month visit, four patients in the usual care group were lost to follow-up whereas no one was missing in the MBSR group. Demographic and baseline characteristics of the study patients are shown in Table 1. There were no significant differences between the two groups. Patients were more frequently suffering from knee than hip with frequent bilateral involvement. We found a frequent association with spine, hand, or shoulder OA localization with 69.2%, 69.2%, 30.8% of patients in the MBSR group and 66.7%, 33.3%, 22.2% in the usual care group, respectively. The functional impact was mild with a limitation perimeter only for three participants in the MBSR group and two in the usual care group.

**Table 1:** Characteristics of knee and hip osteoarthritis patients at baseline

	MBSR group (n = 20)	Usual care group (n = 20)
Demographic		
Men/women	16/4	15/5
Age (years) (mean (SD))	63.0 (7.4)	56.2 (12.9)
BMI (kg/m^2^) (mean (SD))	26.1 (6.1)	28.8 (6.7)
Smoking habits, n (%)	1 (5)	2 (10)
Socio-professional category		
Active, n (%)	5 (25)	9 (45)
Radiological evaluation (left + right) (%)		
At least 1 knee/hip K/L ≥ 2	100	100
Knee/hip K/L = 4	20	10
Clinical symptoms (%)		
Monoarticular knee/hip	15	30
Polyarticular knee/hip	42.5	27.5
Other osteoarthritis symptomatic localisations (N = 22)		
Hand (n (%))	9 (69.2)	3 (33.3)
Spine (n (%))	9 (69.2)	6 (66.7)
Shoulder (n (%))	4 (30.8)	2 (22.2)
Functional impact		
Limitation (n)	3	2
If yes: walking perimeter (m) (mean (SD))	383.3 (293.0)	325.0 (247.5)

*MBSR* mindfulness-based stress reduction, *BMI* body mass index, *K/L* Kellgren and Lawrence scale, *VAS* visual analogic scale, *SD* standard deviation

### Primary outcome

We did not observe any differences in the WOMAC pain score between the two groups at baseline, 3 months, and the final follow-up visit (6 months) (Table 2). The changes in WOMAC pain score between baseline and 3 months was not different between groups (Table 3). However, there was a trend of higher pain relief in the MBSR group (− 16.8 ± 15.2 vs − 10.7 ± 19.3; *p* = 0.33) at 6 months (Table 4).

**Table 2:** Outcomes measures at the baseline, the end of the MBSR program (3 months) (V1), and the final follow-up (6 months) (V2)

	MBSR group	Usual care group	*P* value
*Primary Outcome (mean (SD))*			
WOMAC _pain_			
Baseline	55.6 (16.7) (n = 20)	47.9 (14.8) (n = 20)	0.15
V1	47.2 (17.6) (n = 18)	36.7 (25.7) (n = 12)	0.19
V2	38.0 (19.8) (n = 20)	39.7 (20.3) (n = 15)	0.80
*Secondary Outcomes (mean (SD))*			
WOMAC _function_			
Baseline	49.0 (19.2) (n = 20)	45.5 (14.4) (n = 20)	0.54
V1	43.3 (17.0) (n = 13)	36.4 (24.3) (n = 13)	0.41
V2	38.5 (19.6) (n = 18)	39.7 (22.0) (n = 14)	0.86
WOMAC _stiffness_			
Baseline	59.4 (17.6) (n = 20)	52.2 (22.1) (n = 20)	0.28
V1	55.3 (23.7) (n = 19)	48.3 (19.4) (n = 15)	0.36
V2	40.6 (16.7) (n = 20)	48.4 (25.8) (n = 16)	0.27
WOMAC _total_			
Baseline	55.1 (13.6) (n = 20)	48.6 (14.8) (n = 20)	0.20
V1	46.4 (16.6) (n = 12)	38.4 (22.5) (n = 11)	0.34
V2	40.7 (16.5) (n = 18)	43.7 (22.4) (n = 14)	0.67
VAS _pain_			
Baseline	64.0 (12.2) (n = 20)	60.0 (12.7) (n = 20)	0.30
V1	41.4 (25.3) (n = 20)	44.8 (26.5) (n = 16)	0.70
V2	34.4 (27.2) (n = 20)	48.7 (26.9) (n = 16)	0.12
SF36 _physical score_			
Baseline	34.8 (8.1) (n = 20)	36.1 (8.2) (n = 18)	0.62
V1	36.6 (7.9) (n = 20)	39.5 (9.5) (n = 15)	0.33
V2	38.6 (10.5) (n = 20)	40.1 (11.8) (n = 15)	0.68
SF36 _mental score_			
Baseline	48.1 (9.6) (n = 20)	42.4 (12.0) (n = 20)	0.11
V1	50.7 (7.9) (n = 20)	41.8 (11.4) (n = 15)	0.01*
V2	50.0 (8.6) (n = 20)	41.3 (14.3) (n = 15)	0.11
HAD _anxiety_			
Baseline	7.3 (4.0) (n = 20)	8.4 (4.3) (n = 20)	0.40
V1	5.2 (3.4) (n = 20)	8.4 (4.3) (n = 15)	0.08
V2	6.2 (4.4) (n = 20)	7.5 (4.7) (n = 16)	0.08
HAD _depression_			
Baseline	4.1 (3.3) (n = 20)	5.3 (3.6) (n = 20)	0.28
V1	3.4 (2.2) (n = 20)	4.8 (3.5) (n = 15)	0.14
V2	3.5 (2.9) (n = 20)	5.1 (3.9) (n = 16)	0.21
FFMQ _observation factor_			
Baseline	29.3 (5.5) (n = 19)	26.9 (5.9) (n = 20)	0.20
V1	31.9 (4.2) (n = 20)	26.9 (5.9) (n = 16)	0.03*
V2	32.1 (4.5) (n = 20)	26.6 (5.8) (n = 16)	0.01*
FFMQ _description experience factor_			
Baseline	29.0 (5.4) (n = 19)	28.3 (6.8) (n = 19)	0.74
V1	30.1 (4.7) (n = 20)	29.7 (7.4) (n = 15)	0.87
V2	31.1 (4.7) (n = 20)	30.0 (8.2) (n = 16)	0.83
FFMQ _mindfulness factor_			
Baseline	28.6 (6.3) (n = 19)	27.8 (5.7) (n = 19)	0.70
V1	29.0 (6.5) (n = 19)	28.1 (6.9) (n = 16)	0.71
V2	30.1 (6.6) (n = 20)	29.4 (6.8) (n = 15)	0.76
FFMQ _private event factor_			
Baseline	18.8 (5.0) (n = 19)	21.2 (3.9) (n = 20)	0.11
V1	23.8 (5.7) (n = 19)	19.6 (4.1) (n = 15)	0.02*
V2	23.1 (5.2) (n = 20)	21.2 (4.3) (n = 16)	0.25
FFMQ _no judgment factor_			
Baseline	28.0 (5.8) (n = 20)	26.3 (7.4) (n = 20)	0.41
V1	28.2 (6.2) (n = 19)	28.5 (6.6) (n = 15)	0.86
V2	30.7 (6.4) (n = 20)	27.1 (7.5) (n = 16)	0.13
FFMQ _total mindfulness_			
Baseline	133.2 (17.0) (n = 18)	131.1 (21.8) (n = 18)	0.74
V1	145.0 (20.0) (n = 18)	131.5 (25.0) (n = 13)	0.10
V2	147.0 (17.3) (n = 20)	133.7 (26.4) (n = 15)	0.08

*VAS* visual analogic scale, *WOMAC* Western Ontario and McMaster Universities Osteoarthritis Index, *SF-36* 36-Item Short-Form Health Survey, *FFMQ* Five Facets Mindfulness Questionnaire, *HAD* hospital anxiety and depression

*Significant difference between groups

**Table 3:** Changes in outcomes parameters between baseline and 3 months

	MBSR group	Usual care group	*P* value
VAS (mean (SD))			
Pain	− 22.6 (22.3) (n = 20)	− 15.2 (27.2) (n = 16)	0.37
WOMAC (mean (SD))			
Pain	− 5.3 (14.6) (n = 16)	− 8.3 (24.0) (n = 12)	0.68
Function	− 1.6 (13.3) (n = 11)	− 4.2 (16.2) (n = 12)	0.68
Stiffness	− 2.6 (23.0) (n = 19)	− 1.7 (14.8) (n = 15)	0.87
Total	− 5.7 (11.5) (n = 9)	− 4.5 (16.6) (n = 10)	0.85
SF-36 scale (mean (SD))			
Physical score	1.7 (7.1) (n = 20)	2.5 (8.0) (n = 14)	0.75
Mental score	2.6 (7.9) (n = 20)	1.0 (9.0) (n = 14)	0.59
HAD scale (mean (SD))			
Anxiety	− 2.1 (2.4) (n = 20)	− 0.7 (3.8) (n = 15)	0.17
Depression	− 0.8 (2.4) (n = 20)	− 0.7 (2.8) (n = 15)	0.92
OMERACT-OARSI responders (mean (SD))			
	7.0 (63.6) (n = 11)	5.0 (62.5) (n = 8)	1

*VAS* visual analogic scale, *WOMAC* Western Ontario and McMaster Universities Osteoarthritis Index, *SF-36* 36-Item Short-Form Health Survey, *HAD* hospital anxiety and depression

**Table 4:** Changes in outcomes parameters between baseline and 6 months

	MBSR group	Usual care group	*P* value
VAS (mean (SD))			
Pain	− 29.6 (26.6) (n = 20)	− 9.3 (27.3) (n = 16)	0.03*
WOMAC (mean (SD))			
Pain	− 16.8 (15.2) (n = 17)	− 10.7 (19.3) (n = 14)	0.33
Function	− 13.8 (16.3) (n = 15)	− 8.7 (15.3) (n = 14)	0.39
Stiffness	− 18.8 (17.4) (n = 20)	− 4.7 (29.9) (n = 16)	0.10
Total	− 16.3 (11.9) (n = 14)	− 9.3 (16.5) (n = 13)	0.21
SF36 scale (mean (SD))			
Physical score	3.7 (6.7) (n = 20)	4.2 (7.8) (n = 14)	0.85
Mental score	1.9 (9.5) (n = 20)	0.9 (11.4) (n = 14)	0.90
HAD scale (mean (SD))			
Anxiety	− 1.1 (2.7) (n = 20)	− 0.5 (3.1) (n = 16)	0.54
Depression	− 0.6 (2.4) (n = 20)	− 0.3 (2.8) (n = 16)	0.74
OMERACT-OARSI responders (mean (SD))			
	8.0 (57.1) (n = 14)	5.0 (62.5) (n = 18)	1

*VAS* visual analogic scale, *WOMAC* Western Ontario and McMaster Universities Osteoarthritis Index, *SF-36* 36-Item Short-Form Health Survey, *HAD* hospital anxiety and depression

*Significant difference between groups

### Secondary outcomes

No significant difference between the two groups either in 3-month or in 6-month visit were observed concerning the WOMAC subscales (Function, stiffness and total score) or the OMERACT-OARSI response (Table 2).

We also studied the efficacy of the MBSR program on pain relief by assessing VAS variation. A higher pain reduction but non-significant was found in the MBSR group between baseline and 3 months (− 22.6 ± 22.3 vs − 15.2 ± 27.2; *p* = 0.37) (Table 3). Nevertheless, we observed a significant pain VAS reduction in favor of the MBSR group between baseline and 6 months (− 29.6 ± 26.6 vs − 9.3 ± 27.3; *p* = 0.03) (Table 4).

No differences between the two groups were reported neither after 3 months nor 6 months regarding anxiety/depression (HAD) or quality of life (SF-36). However, it was interesting to note that SF-36 mental score was exclusively improved in the MBSR group (48.1 ± 9.6 at baseline and 50.0 ± 8.6 at 6 months) while a decrease trend in the usual care group was found (42.4 ± 12.0 at baseline and 41.3 ± 14.3 after 6 months) (Table 2).

Finally, we analyzed FFMQ subscores at different time points (Table 2), but we did not find predictive factors associated with a better response to the MBSR program whatever the outcome criteria used (pain VAS, WOMAC subscales) (data not shown).

## Discussion

As far as we know, the present study is the first RCT to prospectively investigate pain and function following a MBSR program in patients with symptomatic knee and hip OA. We mainly found a significant decrease in pain VAS between baseline and 6 months in the MBSR group, which revealed a long-lasting effect of the MBSR program on pain and could be complementary to the immediate action from analgesic medications. In addition, the patients did not experiment significant results in other outcomes parameters (WOMAC, OMERACT-OARSI response, HAD, SF-36 or painkillers consumption) at 3 months or 6 months. Several reasons can explain the lack of differences between the two groups. First, we enrolled a low number of patients due to the exploratory design of the study that could lead to a poor statistical power. The WOMAC score is also more difficult and complicated for patients to understand than the VAS scale [32, 33] and may cause underestimation in the results. For instance, in our study, eight and five patients did not answer to the WOMAC pain questionnaires at 3-month and 6-month visit, respectively, while only four patients missed answering VAS scale at each visit. Furtherly, although no significant difference was noted in anxiety and depression based on HAD score, nor in the quality of life evaluated by SF-36, a trend towards better improvement in each outcome measurement could be observed immediately after the program and 3 months later in the MBSR group. Notably, our results were consistent with the literature regarding HAD score [17, 34–37], and SF-36 score [38, 39].

The reduction of pain and improved function in knee or hip OA after mindfulness workshops have already been reported. Ahn et al. demonstrated a pain reduction (WOMAC pain) in 15 patients with symptomatic knee OA after receiving ten home-based sessions of transcranial direct current stimulation paired with mindfulness over 2 weeks [40]. The study from Dowsey et al. evaluated the efficacy of a MBSR program before total joint arthroplasty in 65 knee OA patients suffering from moderate to severe psychological distress in comparison with 62 patients with usual care management [41]. They found a greater improvement in the mindfulness group for the WOMAC pain (− 10.3 points; 95% CI − 19.0 to − 1.6; *p* = 0.02) and the WOMAC function (− 10.2 points; 95% CI − 19.2 to − 1.3; *p* = 0.03) 12 months post-surgery, but no between group differences were observed at 3-months for any outcome which is similar to the results of ours at the same time point. This might imply the importance of observation of long-term effect of MBSR program. It’s also interesting to point out in their study that participants could only be included if patient’s SF-12 survey mental component summary score was less than 40 points, other than patients in general, which might overrate the efficacy of intervention.

With regard to MBSR assiduousness, we distinguished session assiduousness and home training assiduousness (Additional files 1 and 2: data). We observed a great participation of patients during the program, which can explain good long-lasting results in particular after 6 months i.e. nearly 3 months after the end of the MBSR program. As expected, we found a drop of motivation after the end of the program with a reduction of home training sessions at the 6-month visit since only 7 of 20 patients pursued meditation > 3 times a week (Additional files 1 and 2: data). However, it did not affect the results. In addition, we did not find differences in pain VAS between patients who continued to practice mindfulness > 3 times a week in comparison with those did not, suggesting that a regular mindfulness practice would be sufficient to achieve an improvement in pain and function in OA patients.

Finally, it is worth mentioning that although a significant difference could be observed in a pilot trial, it does not necessarily lead to a definitive conclusion. Conversely, no significant difference between groups does not mean that it would not have significance in the main trial. Therefore, the favorable results of this study are a possibility towards MBSR program for OA patients, but conclusive results will require future studies with larger sample sizes to verify its validity.

The strengths of our study include the RCT design, the use of standardized scales, and the good assiduousness of participants without many losses of follow-up. The non-invasive and safely MBSR program may become an alternative pain treatment strategy in OA care management. Nevertheless, limitations of the study may be acknowledged. Firstly, due to the nature of the intervention group, participants were not blinded, which might affect some outcome measurements, and could be the reason of four participants loss of follow-up in the control group compared to none in the MBSR group at the final visit. It may be due to their disappointment of not receiving active treatment. Secondly, this preliminary study included a small number of patients which could be responsible for the lack of power analysis; also, the follow-up time was relatively short, further research evaluating over 12-month efficacy would be required for this matter. Thirdly, the lack of active comparator, as rehabilitation or cognitive behavioral program prevented us from making comparison among them to draw a more definitive conclusion. Finally, due to the pilot design, the alpha risk was not controlled for secondary outcomes, which needs particular precociousness in their interpretation.

## Conclusion

Our pilot RCT found encouraging results with a delayed but long-term efficacy in pain VAS following a MBSR program in symptomatic knee or hip OA patients. Patients may experiment wider benefits in their daily life with a stress reduction, a quality of life improvement, and less depressive symptoms, compared to usual care. However, future studies with larger sample size are mandatory to confirm these preliminary results.

## Supplementary Information

**Additional file 1.** Painkillers consumption during the 6-month study among all participants.

**Additional file 2.** Analysis of assiduousness to the MBSR workshops.

## Data Availability

The datasets used and/or analysed during the current study are available from the corresponding author on reasonable request.
