# Supplementary material for: Evaluation of mindfulness based stress reduction in symptomatic knee or hip osteoarthritis patients: a pilot randomized controlled trial
Source: BMC Rheumatol. 2022 May 30;6:46. doi: 10.1186/s41927-022-00277-9 (PMC9150306; doi:10.1186/s41927-022-00277-9)
Supplement: Supplementary file 1 — Additional file 1. Painkillers consumption during the 6-month study among all participants. [file 41927_2022_277_MOESM1_ESM.docx]

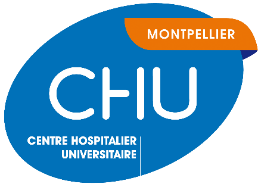


**STATISTICAL ANALYSIS REPORT OF 14/09/2020**

**Title: Randomized controlled trial of a mindfulness program (MBSR) in the management of symptomatic osteoarthritis**

**Principal Investigator: Yves-Marie Pers**

**Statistician : Safa AOUINTI**

Affiliation : Clinical Research and Epidemiology Unit, CHU Montpellier, Univ Montpellier, Montpellier, France

Mail : [s-aouinti@chu-montpellier.fr](mailto:s-aouinti@chu-montpellier.fr)

**Responsible for methodology : Claire DUFLOS**

Affiliation : Clinical Research and Epidemiology Unit, CHU Montpellier, Univ Montpellier, Montpellier, France

Mail : [c-duflos@chu-montpellier.fr](mailto:c-duflos@chu-montpellier.fr)

# Objectives of the study

### Primary objective

The main objective of the project is to evaluate the effectiveness of a mindfulness program as a treatment for hip or knee osteoarthritis on the WOMAC pain score, compared to controlled program.

### secondary Objectives

- Evalute the impact of the program on **pain and patient function**

• Evalute the impact of the programme on **quality of life**

• Evalute the impact the **psychological impact** of the program

• **Identify a patient profil that is prédictive of reponse to the mindfulness program**

# Statistical Analysis

## Type of study

This study involved 40 patients, randomized individually. The intervention group was treated during group sessions, in groups of about 10 participants (i.e. 2 groups). As the facilitator was always the same, this group treatment did not result in a possible center effect.

### EXPECTED NUMBER OF PEOPLE TO BE INCLUDED IN THE STUDY

We will consecutively recruit patients treated in the rheumatology department of the Montpellier University Hospital for knee or hip osteoarthritis. The number of patients included is limited to 40 (20 in each group) because of the pilot nature of this study.

### Description of the statistical analysis plan

The statistical analysis will be performed by the Clinical and Epidemiological Research Unit of the CHU of

Montpellier University Hospital. The significance threshold will be set at 5% for all tests performed.

***Descriptive analysis***

The patient flow chart will be presented. Quantitative variables will be described by their means and standard deviations, and medians and quartiles. Qualitative variables will be described by their numbers and percentages.

**Primary Analysis**

The primary analysis will be conducted on an intention-to-treat basis. The evolution of the WOMAC pain score between inclusion and the end of the program will be compared between the two groups by a Wilcoxon-Mann-Whitney test for independent data.

**Secondairy Analysis**

Analysis of secondary endpoints: quantitative variables will be compared between the two groups by a Wilcoxon-Mann-Whitney test for independent data. The responder rate will be compared between the two groups by the appropriate test (Chi-square or Fisher exact test).

Identification of a profile of responder patients

A multivariate linear model including randomization group, FFMQ score at inclusion, and the interaction between the two will be derived to look for a different effectiveness of the intervention according to the FFMQ score. The treatment of the FFMQ variable (continuous or qualitative) will depend on statistical conditions to be verified on the data.

Total population 40 (20 in each group):

- Classic Group : 20 (- 4 patients left)

# DEFINITION DES VARIABLES NON EXPLICITES

**Formulas used to calculate the total Womac score :**

sc_tot_womac_cal = WOMAC_DOULEUR1 + WOMAC_DOULEUR2 + WOMAC_DOULEUR3 + WOMAC_DOULEUR4 + WOMAC_DOULEUR5 +

WOMAC_FONCTION1 + WOMAC_FONCTION2 + WOMAC_FONCTION3 +

WOMAC_FONCTION4 + WOMAC_FONCTION5 + WOMAC_FONCTION6 + WOMAC_FONCTION7 + WOMAC_FONCTION8 + WOMAC_FONCTION9 + WOMAC_FONCTION10 + WOMAC_FONCTION11+

WOMAC_FONCTION12+ WOMAC_FONCTION13 + WOMAC_FONCTION14 +

WOMAC_FONCTION15 + WOMAC_FONCTION16 + WOMAC_FONCTION17 +

WOMAC_RAIDEUR1 + WOMAC_RAIDEUR2

sc_tot_womac_cal100 = (sc_tot_womac_cal/**96**)***100**

- WOMAC douleur = Sum of the questions in the domain from 1 to 5 (maximum 20).

To obtain the score out of 100, multiply the number by 5.

For example: Total of the questions of the domain from 1 to 5 = 11 or 11 x 5 = 55 on 100
- WOMAC fonction = Sum of the questions in the domain from 1 to 17 (maximum 68).

To obtain the score out of 100, divide the number by 68 and multiply by 100 (rule of 3).

For example: Total of the questions of the domain from 1 to 17 = 27 or 27/68 = 0,397X100 =39.7 out of 100- WOMAC raideur = Sum of the questions in the domain from 1 to 2 (maximum 8).

To obtain the score out of 100, multiply the number by 12.5.

For example: Total of the questions from domain 1 to 2 = 6 or 6 x 12.5 = 75 out of 100

# Résultats

## Flow Chart

ENROLLMENT

**Visit 0**

**(baseline)**

**Discontinued the study (n= 0)**

**Visits 1 & 2**

**MBSR Program (n= 20)**

Discontinued the study (n= 4)

- Patient's refusal to continue the study (n=3)
- lost view (n=1)

ASSESSMENT

**Classic follow-up and general advice (n= 20)**

ANALYSIS

**Participants n= 20**

**Participants n= 16**

**Randomized (n= 40)**

## DESCRIPTION OF THE POPULATION

### INCLUSION VISIT (V0)

#### Sociodemographic data

|  | | | **Group Name** | |  | |
| --- | --- | --- | --- | --- | --- | --- |
| **variable** | **Detalis** | **Total**  **population** | **Program MBSR** | **Classic follow-up and general advice** | **Test** | **p** |
| Sex (n(%col)) |  | N=40 | n=20 | n=20 | FISHER | 1.000 |
|  | Women | 31 (77.50) | 15 (75.00) | 16 (80.00) |  |  |
|  | Men | 9 (22.50) | 5 (25.00) | 4 (20.00) |  |  |
| Age |  | N=40 | n=20 | n=20 | STUDENT | 0.052 |
|  | Mean (± SD) | 59.53 (± 10.94) | 62.90 (± 7.43) | 56.15 (± 12.91) |  |  |
|  | Median (Q1;Q3) | 63.50 (52.00 ; 68.50) | 64.50 (59.50 ; 68.50) | 57.00 (44.00 ; 69.00) |  |  |
|  | Median (min;max) | 63.50 (34.00 ; 74.00) | 64.50 (44.00 ; 74.00) | 57.00 (34.00 ; 74.00) |  |  |
| Professional situation (n(%col)) |  | N=40 | n=20 | n=20 | FISHER | 0.447 |
|  | Activite | 14 (35.00) | 5 (25.00) | 9 (45.00) |  |  |
|  | Retired | 21 (52.50) | 13 (65.00) | 8 (40.00) |  |  |
|  | Disability | 3 (7.50) | 1 (5.00) | 2 (10.00) |  |  |
|  | Unemployed, minimum wage | 2 (5.00) | 1 (5.00) | 1 (5.00) |  |  |

| **Current or past profession** | | | | |
| --- | --- | --- | --- | --- |
| **Occupations** | **Frequency** | **Percentage** | **Frequency cumulative** | **Percentage cumulative** |
| **ADMINISTRATIVE OFFICER** | 1 | 2.56 | 1 | 2.56 |
| **INTENSIVE CARE NURSE'S AIDE** | 1 | 2.56 | 2 | 5.13 |
| **NURSING AID** | 2 | 5.13 | 4 | 10.26 |
| **SOCIAL ASSISTANT** | 1 | 2.56 | 5 | 12.82 |
| **CNRS COMMUNICATION ASSISTANT** | 1 | 2.56 | 6 | 15.38 |
| **CADRE EDF** | 1 | 2.56 | 7 | 17.95 |
| **HEAD OF DEPARTMENT LABO BOIRON** | 1 | 2.56 | 8 | 20.51 |
| **RESEARCHER** | 1 | 2.56 | 9 | 23.08 |
| **COIFFEUSE** | 1 | 2.56 | 10 | 25.64 |
| **MERCHANT** | 1 | 2.56 | 11 | 28.21 |
| **EMPLOYMENT ADVISOR** | 1 | 2.56 | 12 | 30.77 |
| **LIBRARY CURATOR** | 1 | 2.56 | 13 | 33.33 |
| **CAT CONTROLLER** | 1 | 2.56 | 14 | 35.90 |
| **MANAGEMENT SERVICE/CREPS** | 1 | 2.56 | 15 | 38.46 |
| **HOUSEWIFE** | 1 | 2.56 | 16 | 41.03 |
| **TERRITORIAL OFFICIAL** | 1 | 2.56 | 17 | 43.59 |
| **TRAINER IN LAW/ECONOMICS** | 1 | 2.56 | 18 | 46.15 |
| **TRAINER** | 1 | 2.56 | 19 | 48.72 |
| **TRAINER FOR ADULTS** | 1 | 2.56 | 20 | 51.28 |
| **COMPANY MANAGER** | 1 | 2.56 | 21 | 53.85 |
| **ASSET MANAGER** | 1 | 2.56 | 22 | 56.41 |
| **JOURNALIST** | 1 | 2.56 | 23 | 58.97 |
| **MOTHER AT HOME** | 1 | 2.56 | 24 | 61.54 |
| **ORTOPHONIST** | 1 | 2.56 | 25 | 64.10 |
| **OSTEOPATHE** | 1 | 2.56 | 26 | 66.67 |
| **LITERATURE TEACHER** | 1 | 2.56 | 27 | 69.23 |
| **NATIONAL EDUCATION TEACHER** | 1 | 2.56 | 28 | 71.79 |
| **PSYCHOLOGIST** | 1 | 2.56 | 29 | 74.36 |
| **NURSE** | 1 | 2.56 | 30 | 76.92 |
| **PEDIATRIC EMERGENCY NURSE** | 1 | 2.56 | 31 | 79.49 |
| **PURCHASING MANAGER** | 1 | 2.56 | 32 | 82.05 |
| **STORE MANAGER (FRANCE TELECOM)** | 1 | 2.56 | 33 | 84.62 |
| **IT MANAGER** | 1 | 2.56 | 34 | 87.18 |
| **SECRETARY** | 1 | 2.56 | 35 | 89.74 |
| **MEDICAL SCHOOL SECRETARY** | 1 | 2.56 | 36 | 92.31 |
| **SOPHROLOGIST** | 1 | 2.56 | 37 | 94.87 |
| **SALESWOMAN IN GARDEN CENTER** | 1 | 2.56 | 38 | 97.44 |
| **SALES** | 1 | 2.56 | 39 | 100.00 |
| **Missing frequency = 1** | | | | |

|  | | | **Group Name** | |  | |
| --- | --- | --- | --- | --- | --- | --- |
| **variable** | **Details** | **Total**  **population** | **Program MBSR** | **Classic follow-up and general advice** | **Test** | **p** |
| Smoking (n(%col)) |  | N=40 | n=20 | n=20 | FISHER | 0.612 |
|  | Never smoked | 24 (60.00) | 11 (55.00) | 13 (65.00) |  |  |
|  | Current smoker | 3 (7.50) | 1 (5.00) | 2 (10.00) |  |  |
|  | Former smoker | 13 (32.50) | 8 (40.00) | 5 (25.00) |  |  |
| Year quit smoking (n(%col)) |  | N=13 | n=8 | n=5 | FISHER | 1.000 |
|  | 1984 | 1 (7.69) | 1 (12.50) | 0 (0.00) |  |  |
|  | 1992 | 1 (7.69) | 1 (12.50) | 0 (0.00) |  |  |
|  | 1998 | 1 (7.69) | 1 (12.50) | 0 (0.00) |  |  |
|  | 2003 | 3 (23.08) | 1 (12.50) | 2 (40.00) |  |  |
|  | 2006 | 1 (7.69) | 1 (12.50) | 0 (0.00) |  |  |
|  | 2008 | 2 (15.38) | 1 (12.50) | 1 (20.00) |  |  |
|  | 2013 | 1 (7.69) | 1 (12.50) | 0 (0.00) |  |  |
|  | 2016 | 1 (7.69) | 0 (0.00) | 1 (20.00) |  |  |
|  | 2017 | 1 (7.69) | 1 (12.50) | 0 (0.00) |  |  |
|  | 2018 | 1 (7.69) | 0 (0.00) | 1 (20.00) |  |  |
| No. of packages year |  | N=16 | n=9 | n=7 | STUDENT | 0.047 |
|  | Mean (± SD) | 24.44 (± 24.00) | 34.22 (± 27.85) | 11.86 (± 8.78) |  |  |
|  | Median (Q1;Q3) | 15.00 (7.50 ; 40.00) | 40.00 (10.00 ; 46.00) | 9.00 (5.00 ; 21.00) |  |  |
|  | Mwdian (min;max) | 15.00 (1.00 ; 90.00) | 40.00 (5.00 ; 90.00) | 9.00 (1.00 ; 25.00) |  |  |

#### Histoire de la maladie

|  | | | **Group Name** | |  | |
| --- | --- | --- | --- | --- | --- | --- |
| **variable** | **Details** | **Total**  **population** | **Program MBSR** | **Classic follow-up and general advice** | **Test** | **p** |
| Gonarthrosis (n(%col)) |  | N=40 | n=20 | n=20 | FISHER | 1.000 |
|  | No | 1 (2.50) | 1 (5.00) | 0 (0.00) |  |  |
|  | Yes | 39 (97.50) | 19 (95.00) | 20 (100.00) |  |  |
| Gonarthrosis side (n(%col)) |  | N=39 | n=19 | n=20 | FISHER | 0.020 |
|  | Right | 4 (10.26) | 1 (5.26) | 3 (15.00) |  |  |
|  | Left | 8 (20.51) | 1 (5.26) | 7 (35.00) |  |  |
|  | Right and left | 27 (69.23) | 17 (89.47) | 10 (50.00) |  |  |
| Right Gonarthrosis Stage (n(%col)) |  | N=29 | n=17 | n=12 | FISHER | 0.932 |
|  | I | 1 (3.45) | 1 (5.88) | 0 (0.00) |  |  |
|  | II | 15 (51.72) | 8 (47.06) | 7 (58.33) |  |  |
|  | III | 7 (24.14) | 4 (23.53) | 3 (25.00) |  |  |
|  | IV | 6 (20.69) | 4 (23.53) | 2 (16.67) |  |  |
| Left Gonarthrosis Stage (n(%col)) |  | N=35 | n=18 | n=17 | FISHER | 0.494 |
|  | II | 15 (42.86) | 6 (33.33) | 9 (52.94) |  |  |
|  | III | 14 (40.00) | 8 (44.44) | 6 (35.29) |  |  |
|  | IV | 6 (17.14) | 4 (22.22) | 2 (11.76) |  |  |
| Coxarthrosis (n(%col)) |  | N=40 | n=20 | n=20 | FISHER | 1.000 |
|  | No | 33 (82.50) | 16 (80.00) | 17 (85.00) |  |  |
|  | Yes | 7 (17.50) | 4 (20.00) | 3 (15.00) |  |  |
| Right Coxarthrosis Stage (n(%col)) |  | N=6 | n=3 | n=3 | FISHER | 0.400 |
|  | I | 1 (16.67) | 1 (33.33) | 0 (0.00) |  |  |
|  | II | 4 (66.67) | 1 (33.33) | 3 (100.00) |  |  |
|  | III | 1 (16.67) | 1 (33.33) | 0 (0.00) |  |  |
| Left Coxarthrosis Stage (n(%col)) |  | N=2 | n=1 | n=1 | FISHER | 1.000 |
|  | I | 1 (50.00) | 1 (100.00) | 0 (0.00) |  |  |
|  | II | 1 (50.00) | 0 (0.00) | 1 (100.00) |  |  |
| CI2 OK (n(%col)) |  | N=40 | n=20 | n=20 | INVALID | . |
|  | Yes | 40 (100.00) | 20 (100.00) | 20 (100.00) |  |  |
| Other arthritis (n(%col)) |  | N=40 | n=20 | n=20 | CHI2 | 0.342 |
|  | No | 19 (47.50) | 8 (40.00) | 11 (55.00) |  |  |
|  | Yes | 21 (52.50) | 12 (60.00) | 9 (45.00) |  |  |
| hand arthrosis (n(%col)) |  | N=22 | n=13 | n=9 | FISHER | 0.192 |
|  | No | 10 (45.45) | 4 (30.77) | 6 (66.67) |  |  |
|  | Yes | 12 (54.55) | 9 (69.23) | 3 (33.33) |  |  |
| shoulder arthrosis (n(%col)) |  | N=22 | n=13 | n=9 | FISHER | 1.000 |
|  | No | 16 (72.73) | 9 (69.23) | 7 (77.78) |  |  |
|  | Yes | 6 (27.27) | 4 (30.77) | 2 (22.22) |  |  |
| arthrosis of the spine (n(%col)) |  | N=22 | n=13 | n=9 | FISHER | 1.000 |
|  | No | 7 (31.82) | 4 (30.77) | 3 (33.33) |  |  |
|  | Yes | 15 (68.18) | 9 (69.23) | 6 (66.67) |  |  |

**Note:** Missing data for the variables highlighted in green in the previous table were filled in with zeros in the case where the joint was not affected based on the location/side of the OA.

A new variable "At least one joint in stage III or IV" was created.

|  | | | **Group Name** | |  | |
| --- | --- | --- | --- | --- | --- | --- |
| **variable** | **Details** | **Total population** | **Program MBSR** | **Classic follow-up and general advice** | **Test** | **p** |
| Right Gonarthrosis Stage (n(%col)) |  | N=38 | n=19 | n=19 | FISHER | 0.331 |
|  | 0 | 9 (23.68) | 2 (10.53) | 7 (36.84) |  |  |
|  | I | 1 (2.63) | 1 (5.26) | 0 (0.00) |  |  |
|  | II | 15 (39.47) | 8 (42.11) | 7 (36.84) |  |  |
|  | III | 7 (18.42) | 4 (21.05) | 3 (15.79) |  |  |
|  | IV | 6 (15.79) | 4 (21.05) | 2 (10.53) |  |  |
| Right Gonarthrosis Stage (n(%col)) |  | N=38 | n=19 | n=19 | FISHER | 0.331 |
|  | 0 | 9 (23.68) | 2 (10.53) | 7 (36.84) |  |  |
|  | I | 1 (2.63) | 1 (5.26) | 0 (0.00) |  |  |
|  | II | 15 (39.47) | 8 (42.11) | 7 (36.84) |  |  |
|  | III | 7 (18.42) | 4 (21.05) | 3 (15.79) |  |  |
|  | IV | 6 (15.79) | 4 (21.05) | 2 (10.53) |  |  |
| Left Gonarthrosis Stage (n(%col)) |  | N=40 | n=20 | n=20 | FISHER | 0.667 |
|  | 0 | 5 (12.50) | 2 (10.00) | 3 (15.00) |  |  |
|  | II | 15 (37.50) | 6 (30.00) | 9 (45.00) |  |  |
|  | III | 14 (35.00) | 8 (40.00) | 6 (30.00) |  |  |
|  | IV | 6 (15.00) | 4 (20.00) | 2 (10.00) |  |  |
| Left Gonarthrosis Stage (n(%col)) |  | N=40 | n=20 | n=20 | FISHER | 0.667 |
|  | 0 | 5 (12.50) | 2 (10.00) | 3 (15.00) |  |  |
|  | II | 15 (37.50) | 6 (30.00) | 9 (45.00) |  |  |
|  | III | 14 (35.00) | 8 (40.00) | 6 (30.00) |  |  |
|  | IV | 6 (15.00) | 4 (20.00) | 2 (10.00) |  |  |
| Right Coxarthrosis Stage (n(%col)) |  | N=39 | n=19 | n=20 | FISHER | 0.605 |
|  | 0 | 33 (84.62) | 16 (84.21) | 17 (85.00) |  |  |
|  | I | 1 (2.56) | 1 (5.26) | 0 (0.00) |  |  |
|  | II | 4 (10.26) | 1 (5.26) | 3 (15.00) |  |  |
|  | III | 1 (2.56) | 1 (5.26) | 0 (0.00) |  |  |
| Right Coxarthrosis Stage (n(%col)) |  | N=39 | n=19 | n=20 | FISHER | 0.605 |
|  | 0 | 33 (84.62) | 16 (84.21) | 17 (85.00) |  |  |
|  | I | 1 (2.56) | 1 (5.26) | 0 (0.00) |  |  |
|  | II | 4 (10.26) | 1 (5.26) | 3 (15.00) |  |  |
|  | III | 1 (2.56) | 1 (5.26) | 0 (0.00) |  |  |
| Left Coxarthrosis Stage (n(%col)) |  | N=39 | n=19 | n=20 | FISHER | 1.000 |
|  | 0 | 37 (94.87) | 18 (94.74) | 19 (95.00) |  |  |
|  | I | 1 (2.56) | 1 (5.26) | 0 (0.00) |  |  |
|  | II | 1 (2.56) | 0 (0.00) | 1 (5.00) |  |  |
| Left Coxarthrosis Stage (n(%col)) |  | N=39 | n=19 | n=20 | FISHER | 1.000 |
|  | 0 | 37 (94.87) | 18 (94.74) | 19 (95.00) |  |  |
|  | I | 1 (2.56) | 1 (5.26) | 0 (0.00) |  |  |
|  | II | 1 (2.56) | 0 (0.00) | 1 (5.00) |  |  |
| At least one joint in stage III or IV (n(%col)) |  | N=39 | n=20 | n=19 | CHI2 | 0.076 |
|  | No | 15 (38.46) | 5 (25.00) | 10 (52.63) |  |  |
|  | Yes | 24 (61.54) | 15 (75.00) | 9 (47.37) |  |  |

|  | | | **Group Name** | |  | |
| --- | --- | --- | --- | --- | --- | --- |
| **variable** | **Details** | **total population** | **Program MBSR** | **Classic follow-up and general advice** | **Test** | **p** |
| At least one joint in stage II or III or IV (n(% col)) |  | N=40 | n=20 | n=20 | INVALID | . |
|  | 1 | 40 (100.00) | 20 (100.00) | 20 (100.00) |  |  |

#### Clinical examinations

|  | | | **Group Name** | |  | |
| --- | --- | --- | --- | --- | --- | --- |
| **variable** | **detail** | **Total population** | **Program MBSR** | **Classic follow-up and general advice** | **test** | **p** |
| Weight |  | N=40 | n=20 | n=20 | STUDENT | 0.261 |
|  | Mean (± SD) | 74.78 (± 18.91) | 71.38 (± 19.68) | 78.18 (± 17.95) |  |  |
|  | Median (Q1;Q3) | 71.00 (60.25 ; 84.00) | 68.00 (54.00 ; 84.00) | 77.50 (65.50 ; 85.50) |  |  |
|  | Median (min;max) | 71.00 (46.40 ; 121.70) | 68.00 (46.40 ; 121.70) | 77.50 (56.00 ; 120.00) |  |  |
| BMI |  | N=40 | n=20 | n=20 | STUDENT | 0.190 |
|  | Mean (± SD) | 27.42 (± 6.47) | 26.07 (± 6.14) | 28.78 (± 6.66) |  |  |
|  | Median (Q1;Q3) | 26.78 (22.72 ; 29.40) | 25.77 (20.90 ; 29.12) | 27.23 (24.25 ; 31.37) |  |  |
|  | Median (min;max) | 26.78 (17.72 ; 47.47) | 25.77 (17.72 ; 41.67) | 27.23 (19.38 ; 47.47) |  |  |
| Hight |  | N=40 | n=20 | n=20 | WMW | 0.674 |
|  | Mean (± SD) | 164.98 (± 8.65) | 165.00 (± 10.06) | 164.95 (± 7.24) |  |  |
|  | Median (Q1;Q3) | 164.00 (159.00 ; 170.00) | 162.00 (159.50 ; 169.00) | 167.00 (159.00 ; 170.50) |  |  |
|  | Median (min;max) | 164.00 (151.00 ; 191.00) | 162.00 (152.00 ; 191.00) | 167.00 (151.00 ; 175.00) |  |  |
| BP Systole |  | N=37 | n=19 | n=18 | STUDENT | 0.736 |
|  | Mean (± SD) | 134.30 (± 19.76) | 133.21 (± 20.07) | 135.44 (± 19.94) |  |  |
|  | Median (Q1;Q3) | 139.00 (118.00 ; 148.00) | 138.00 (116.00 ; 148.00) | 141.50 (118.00 ; 148.00) |  |  |
|  | Median (min;max) | 139.00 (101.00 ; 171.00) | 138.00 (101.00 ; 168.00) | 141.50 (102.00 ; 171.00) |  |  |
| BP Diastole |  | N=37 | n=19 | n=18 | STUDENT | 0.654 |
|  | Mean (± SD) | 80.00 (± 12.93) | 79.05 (± 14.12) | 81.00 (± 11.88) |  |  |
|  | Median (Q1;Q3) | 81.00 (71.00 ; 88.00) | 80.00 (71.00 ; 85.00) | 82.00 (71.00 ; 90.00) |  |  |
|  | Median (min;max) | 81.00 (54.00 ; 108.00) | 80.00 (54.00 ; 108.00) | 82.00 (61.00 ; 100.00) |  |  |

##### Clinical examination of joints

|  | | | **Group Name** | |  | |
| --- | --- | --- | --- | --- | --- | --- |
| **variable** | **details** | **Total population** | **Program MBSR** | **Classic follow-up and general advice** | **Test** | **p** |
| Pain in the right knee (n(%col)) |  | N=40 | n=20 | n=20 | FISHER | 0.348 |
|  | No | 23 (57.50) | 11 (55.00) | 12 (60.00) |  |  |
|  | Yes | 15 (37.50) | 9 (45.00) | 6 (30.00) |  |  |
|  | NA | 2 (5.00) | 0 (0.00) | 2 (10.00) |  |  |
| Right knee effusion (n(%col)) |  | N=40 | n=20 | n=20 | FISHER | 0.487 |
|  | No | 37 (92.50) | 18 (90.00) | 19 (95.00) |  |  |
|  | Yes | 2 (5.00) | 2 (10.00) | 0 (0.00) |  |  |
|  | NA | 1 (2.50) | 0 (0.00) | 1 (5.00) |  |  |
| Femoral patellar syndrome right knee (n(%col)) |  | N=40 | n=20 | n=20 | FISHER | 1.000 |
|  | No | 22 (55.00) | 11 (55.00) | 11 (55.00) |  |  |
|  | Yes | 15 (37.50) | 8 (40.00) | 7 (35.00) |  |  |
|  | NA | 3 (7.50) | 1 (5.00) | 2 (10.00) |  |  |
| Flessum right knee (n(%col)) |  | N=40 | n=20 | n=20 | FISHER | 1.000 |
|  | No | 35 (87.50) | 17 (85.00) | 18 (90.00) |  |  |
|  | Yes | 3 (7.50) | 2 (10.00) | 1 (5.00) |  |  |
|  | NA | 2 (5.00) | 1 (5.00) | 1 (5.00) |  |  |
| Pain in the left knee O/N (n(%col)) |  | N=40 | n=20 | n=20 | FISHER | 0.341 |
|  | No | 21 (52.50) | 9 (45.00) | 12 (60.00) |  |  |
|  | Yes | 18 (45.00) | 11 (55.00) | 7 (35.00) |  |  |
|  | NA | 1 (2.50) | 0 (0.00) | 1 (5.00) |  |  |
| Left knee effusion (n(%col)) |  | N=40 | n=20 | n=20 | FISHER | 1.000 |
|  | No | 36 (90.00) | 18 (90.00) | 18 (90.00) |  |  |
|  | Yes | 3 (7.50) | 2 (10.00) | 1 (5.00) |  |  |
|  | NA | 1 (2.50) | 0 (0.00) | 1 (5.00) |  |  |
| Femoral patellar syndrome left knee (n(%col)) |  | N=40 | n=20 | n=20 | FISHER | 1.000 |
|  | No | 23 (57.50) | 12 (60.00) | 11 (55.00) |  |  |
|  | Yes | 16 (40.00) | 8 (40.00) | 8 (40.00) |  |  |
|  | NA | 1 (2.50) | 0 (0.00) | 1 (5.00) |  |  |
| Flessum left knee (n(%col)) |  | N=40 | n=20 | n=20 | FISHER | 0.737 |
|  | No | 36 (90.00) | 17 (85.00) | 19 (95.00) |  |  |
|  | Yes | 2 (5.00) | 2 (10.00) | 0 (0.00) |  |  |
|  | NA | 2 (5.00) | 1 (5.00) | 1 (5.00) |  |  |
| Right hip pain Y/N (n(%col)) |  | N=40 | n=20 | n=20 | FISHER | 1.000 |
|  | No | 9 (22.50) | 5 (25.00) | 4 (20.00) |  |  |
|  | Yes | 4 (10.00) | 2 (10.00) | 2 (10.00) |  |  |
|  | NA | 27 (67.50) | 13 (65.00) | 14 (70.00) |  |  |
| Right hip Y/N (n(%col)) |  | N=40 | n=20 | n=20 | CHI2 | 0.723 |
|  | No | 11 (27.50) | 6 (30.00) | 5 (25.00) |  |  |
|  | NA | 29 (72.50) | 14 (70.00) | 15 (75.00) |  |  |
| Left hip pain Y/N (n(%col)) |  | N=40 | n=20 | n=20 | FISHER | 0.314 |
|  | No | 11 (27.50) | 7 (35.00) | 4 (20.00) |  |  |
|  | Yes | 2 (5.00) | 0 (0.00) | 2 (10.00) |  |  |
|  | NA | 27 (67.50) | 13 (65.00) | 14 (70.00) |  |  |
| Flessum left hip Y/N (n(%col)) |  | N=40 | n=20 | n=20 | CHI2 | 0.723 |
|  | No | 11 (27.50) | 6 (30.00) | 5 (25.00) |  |  |
|  | NA | 29 (72.50) | 14 (70.00) | 15 (75.00) |  |  |

|  | | | **Group Name** | |  | |
| --- | --- | --- | --- | --- | --- | --- |
| **variable** | **details** | **Total population** | **Program MBSR** | **Classic follow-up and general advice** | **Test** | **p** |
| Bilateral knee pain (n(% col)) |  | N=37 | n=20 | n=17 | CHI2 | 0.447 |
|  | No | 26 (70.27) | 13 (65.00) | 13 (76.47) |  |  |
|  | Yes | 11 (29.73) | 7 (35.00) | 4 (23.53) |  |  |
| Unilateral knee pain (n(% col)) |  | N=40 | n=20 | n=20 | CHI2 | 0.465 |
|  | No | 30 (75.00) | 14 (70.00) | 16 (80.00) |  |  |
|  | Yes | 10 (25.00) | 6 (30.00) | 4 (20.00) |  |  |
| Bilateral knee effusion (n(% col)) |  | N=38 | n=20 | n=18 | INVALID | . |
|  | No | 38 (100.00) | 20 (100.00) | 18 (100.00) |  |  |
| Unilateral knee effusion (n(% col)) |  | N=40 | n=20 | n=20 | FISHER | 0.342 |
|  | No | 35 (87.50) | 16 (80.00) | 19 (95.00) |  |  |
|  | Yes | 5 (12.50) | 4 (20.00) | 1 (5.00) |  |  |
| At least one knee effusion (n(% col)) |  | N=40 | n=20 | n=20 | FISHER | 0.342 |
|  | No | 35 (87.50) | 16 (80.00) | 19 (95.00) |  |  |
|  | Yes | 5 (12.50) | 4 (20.00) | 1 (5.00) |  |  |
| Bilateral femoro patellar knee syndrome (n(% col)) |  | N=36 | n=19 | n=17 | FISHER | 1.000 |
|  | No | 26 (72.22) | 14 (73.68) | 12 (70.59) |  |  |
|  | Yes | 10 (27.78) | 5 (26.32) | 5 (29.41) |  |  |
| Unilateral femoral patellar knee syndrome (n(% col)) |  | N=40 | n=20 | n=20 | FISHER | 1.000 |
|  | No | 31 (77.50) | 15 (75.00) | 16 (80.00) |  |  |
|  | Yes | 9 (22.50) | 5 (25.00) | 4 (20.00) |  |  |
| At least one femoro patellar knee syndrome (n(% col)) |  | N=40 | n=20 | n=20 | CHI2 | 0.752 |
|  | No | 19 (47.50) | 9 (45.00) | 10 (50.00) |  |  |
|  | Yes | 21 (52.50) | 11 (55.00) | 10 (50.00) |  |  |
| Flessum knee bilateral (n(% col)) |  | N=37 | n=19 | n=18 | FISHER | 1.000 |
|  | No | 36 (97.30) | 18 (94.74) | 18 (100.00) |  |  |
|  | Yes | 1 (2.70) | 1 (5.26) | 0 (0.00) |  |  |
| Unilateral knee flessum (n(% col)) |  | N=39 | n=19 | n=20 | FISHER | 0.605 |
|  | No | 36 (92.31) | 17 (89.47) | 19 (95.00) |  |  |
|  | Yes | 3 (7.69) | 2 (10.53) | 1 (5.00) |  |  |
| At least one knee flessum (n(% col)) |  | N=39 | n=19 | n=20 | FISHER | 0.342 |
|  | No | 35 (89.74) | 16 (84.21) | 19 (95.00) |  |  |
|  | Yes | 4 (10.26) | 3 (15.79) | 1 (5.00) |  |  |
| Bilateral hip pain (n(% col)) |  | N=13 | n=7 | n=6 | FISHER | 0.462 |
|  | No | 12 (92.31) | 7 (100.00) | 5 (83.33) |  |  |
|  | Yes | 1 (7.69) | 0 (0.00) | 1 (16.67) |  |  |
| Unilateral hip pain (n(% col)) |  | N=13 | n=7 | n=6 | FISHER | 1.000 |
|  | No | 9 (69.23) | 5 (71.43) | 4 (66.67) |  |  |
|  | Yes | 4 (30.77) | 2 (28.57) | 2 (33.33) |  |  |
| Bilateral hip flessum (n(% col)) |  | N=11 | n=6 | n=5 | INVALID | . |
|  | No | 11 (100.00) | 6 (100.00) | 5 (100.00) |  |  |
| Unilateral hip flessum (n(% col)) |  | N=11 | n=6 | n=5 | INVALID | . |
|  | No | 11 (100.00) | 6 (100.00) | 5 (100.00) |  |  |
| At least one hip flessum (n(% col)) |  | N=11 | n=6 | n=5 | INVALID | . |
|  | No | 11 (100.00) | 6 (100.00) | 5 (100.00) |  |  |

|  | | | **Group Name** | |  | |
| --- | --- | --- | --- | --- | --- | --- |
| **variable** | **details** | **Total population** | **Program**  **MBSR** | **Classic follow-up and general advice** | **Test** | **p** |
| At least one painful joint (n(% col)) |  | N=25 | n=14 | n=11 | INVALID | . |
|  | Yes | 25 (100.00) | 14 (100.00) | 11 (100.00) |  |  |

##### Hip passive mobility

|  | | | **Group Name** | |  | |
| --- | --- | --- | --- | --- | --- | --- |
| **variable** | **details** | **Total population** | **Program MBSR** | **Classic follow-up and general advice** | **test** | **p** |
| Right hip flexion |  | N=31 | n=16 | n=15 | WMW | 0.704 |
|  | Mean (± SD) | 131.29 (± 22.51) | 133.75 (± 17.56) | 128.67 (± 27.22) |  |  |
|  | Median (Q1;Q3) | 130.00 (120.00 ; 145.00) | 132.50 (130.00 ; 142.50) | 130.00 (120.00 ; 145.00) |  |  |
|  | Median (min;max) | 130.00 (45.00 ; 160.00) | 132.50 (90.00 ; 160.00) | 130.00 (45.00 ; 160.00) |  |  |
| Right hip abduction |  | N=36 | n=18 | n=18 | WMW | 0.741 |
|  | Mean (± SD) | 52.92 (± 10.91) | 53.89 (± 7.96) | 51.94 (± 13.41) |  |  |
|  | Médiane (Q1;Q3) | 60.00 (45.00 ; 60.00) | 60.00 (45.00 ; 60.00) | 55.00 (45.00 ; 60.00) |  |  |
|  | Median(min;max) | 60.00 (20.00 ; 80.00) | 60.00 (40.00 ; 60.00) | 55.00 (20.00 ; 80.00) |  |  |
| Internal rotation right hip ° |  | N=36 | n=18 | n=18 | WMW | 0.818 |
|  | Mean (± SD) | 32.92 (± 13.59) | 33.06 (± 16.55) | 32.78 (± 10.32) |  |  |
|  | Median (Q1;Q3) | 30.00 (30.00 ; 42.50) | 30.00 (30.00 ; 45.00) | 30.00 (30.00 ; 40.00) |  |  |
|  | Mwdian (min;max) | 30.00 (10.00 ; 80.00) | 30.00 (10.00 ; 80.00) | 30.00 (10.00 ; 45.00) |  |  |
|  |  | N=36 | n=18 | n=18 | WMW | 0.115 |
|  | Mean (± SD) | 40.97 (± 9.17) | 42.78 (± 6.24) | 39.17 (± 11.28) |  |  |
|  | Median (Q1;Q3) | 45.00 (37.50 ; 45.00) | 45.00 (40.00 ; 45.00) | 40.00 (30.00 ; 45.00) |  |  |
|  | Median (min;max) | 45.00 (15.00 ; 60.00) | 45.00 (20.00 ; 50.00) | 40.00 (15.00 ; 60.00) |  |  |
| Left hip flexion |  | N=31 | n=16 | n=15 | WMW | 0.442 |
|  | Mean (± SD) | 130.32 (± 27.54) | 136.25 (± 14.66) | 124.00 (± 36.21) |  |  |
|  | Median (Q1;Q3) | 130.00 (130.00 ; 145.00) | 137.50 (130.00 ; 142.50) | 130.00 (120.00 ; 145.00) |  |  |
|  | Median (min;max) | 130.00 (35.00 ; 160.00) | 137.50 (100.00 ; 160.00) | 130.00 (35.00 ; 160.00) |  |  |
| Left hip abduction |  | N=36 | n=18 | n=18 | WMW | 0.605 |
|  | Mean (± SD) | 54.31 (± 11.90) | 55.83 (± 9.74) | 52.78 (± 13.85) |  |  |
|  | Median (Q1;Q3) | 60.00 (45.00 ; 60.00) | 60.00 (45.00 ; 60.00) | 60.00 (45.00 ; 60.00) |  |  |
|  | Median (min;max) | 60.00 (20.00 ; 80.00) | 60.00 (40.00 ; 80.00) | 60.00 (20.00 ; 80.00) |  |  |
| Internal rotation left hip ° |  | N=36 | n=18 | n=18 | WMW | 0.583 |
|  | Mean (± SD) | 33.61 (± 11.63) | 34.17 (± 13.09) | 33.06 (± 10.31) |  |  |
|  | Median (Q1;Q3) | 30.00 (30.00 ; 40.00) | 30.00 (30.00 ; 35.00) | 32.50 (30.00 ; 40.00) |  |  |
|  | Median (min;max) | 30.00 (10.00 ; 80.00) | 30.00 (20.00 ; 80.00) | 32.50 (10.00 ; 45.00) |  |  |
| External rotation of the left hip °. |  | N=36 | n=18 | n=18 | WMW | 0.501 |
|  | Mean (± SD) | 43.19 (± 7.94) | 42.22 (± 6.91) | 44.17 (± 8.95) |  |  |
|  | Median (Q1;Q3) | 45.00 (40.00 ; 45.00) | 45.00 (40.00 ; 45.00) | 45.00 (40.00 ; 45.00) |  |  |
|  | Median (min;max) | 45.00 (20.00 ; 60.00) | 45.00 (20.00 ; 50.00) | 45.00 (20.00 ; 60.00) |  |  |

##### Walking perimeter

|  | | | **Group Name** | |  | |
| --- | --- | --- | --- | --- | --- | --- |
| **variable** | **details** | **Total population** | **Program MBSR** | **Classic follow-up and general advice** | **test** | **p** |
| Walking range (m) |  | N=5 | n=3 | n=2 | STUDENT | 0.833 |
|  | Mean (± SD) | 360.00 (± 243.41) | 383.33 (± 292.97) | 325.00 (± 247.49) |  |  |
|  | Median (Q1;Q3) | 500.00 (150.00 ; 500.00) | 500.00 (50.00 ; 600.00) | 325.00 (150.00 ; 500.00) |  |  |
|  | Median (min;max) | 500.00 (50.00 ; 600.00) | 500.00 (50.00 ; 600.00) | 325.00 (150.00 ; 500.00) |  |  |
| No limitation of walking perimeter (>1 km) (n(%col)) |  | N=35 | n=17 | n=18 | INVALID | . |
|  | Yes | 35 (100.00) | 17 (100.00) | 18 (100.00) |  |  |

#### Analgesics and/or NSAIDs during the last 7 days

|  | | | **Group Name** | |  | |
| --- | --- | --- | --- | --- | --- | --- |
| **variable** | **details** | **total population** | **Program MBSR** | **Classic follow-up and general advice** | **Test** | **p** |
| Analgesic/NSAID use Y/N (n(%col)) |  | N=40 | n=20 | n=20 | CHI2 | 1.000 |
|  | No | 28 (70.00) | 14 (70.00) | 14 (70.00) |  |  |
|  | Yes | 12 (30.00) | 6 (30.00) | 6 (30.00) |  |  |
| Current Analgesic 1 (n(%col)) |  | N=5 | n=4 | n=1 | INVALID | . |
|  | Yes | 5 (100.00) | 4 (100.00) | 1 (100.00) |  |  |
| Name of Analgesic 1 (n(%col)) |  | N=12 | n=6 | n=6 | FISHER | 0.015 |
|  | 1159 \| ARCOXIA | 1 (8.33) | 1 (16.67) | 0 (0.00) |  |  |
|  | 1265 \| ASPEGIC | 1 (8.33) | 0 (0.00) | 1 (16.67) |  |  |
|  | 13109 \| SPIFEN (CP) | 1 (8.33) | 1 (16.67) | 0 (0.00) |  |  |
|  | 14083 \| TRAMADOLARROW | 1 (8.33) | 1 (16.67) | 0 (0.00) |  |  |
|  | 15099 \| VOLTARENE (CPGASTRORESIS) | 1 (8.33) | 1 (16.67) | 0 (0.00) |  |  |
|  | 15103 \| VOLTARENELP | 1 (8.33) | 1 (16.67) | 0 (0.00) |  |  |
|  | 4548 \| DOLIPRANE (CP) | 5 (41.67) | 0 (0.00) | 5 (83.33) |  |  |
|  | 9462 \| NAPROXENETEVA | 1 (8.33) | 1 (16.67) | 0 (0.00) |  |  |
| Tablet Dose (mg) Antalgic 1 |  | N=12 | n=6 | n=6 | WMW | 0.002 |
|  | Mean (± SD) | 608.33 (± 434.11) | 216.67 (± 215.47) | 1000.00 (± 0.00) |  |  |
|  | Median (Q1;Q3) | 775.00 (150.00 ; 1000.00) | 150.00 (30.00 ; 400.00) | 1000.00 (1000.00 ; 1000.00) |  |  |
|  | Median (min;max) | 775.00 (20.00 ; 1000.00) | 150.00 (20.00 ; 550.00) | 1000.00 (1000.00 ; 1000.00) |  |  |
| No. of tablets per week Antalgic 1 |  | N=12 | n=6 | n=6 | WMW | 0.028 |
|  | Mean (± SD) | 5.17 (± 4.17) | 7.83 (± 3.97) | 2.50 (± 2.35) |  |  |
|  | Median (Q1;Q3) | 5.00 (1.50 ; 7.00) | 7.00 (7.00 ; 10.00) | 1.50 (1.00 ; 3.00) |  |  |
|  | Median (min;max) | 5.00 (1.00 ; 14.00) | 7.00 (2.00 ; 14.00) | 1.50 (1.00 ; 7.00) |  |  |
| Current Analgesic 2 (n(%col)) |  | N=2 | n=2 | n=0 | INVALID | . |
|  | Yes | 2 (100.00) | 2 (100.00) | 0 (0.00) |  |  |
| Name Analgesic 2 (n(%col)) |  | N=3 | n=3 | n=0 | INVALID | . |
|  | 10632 \| PARACETAMOLARROW (CP) | 1 (33.33) | 1 (33.33) | 0 (0.00) |  |  |
|  | 22879 \| IBUPROFEN TEVA SANTE | 1 (33.33) | 1 (33.33) | 0 (0.00) |  |  |
|  | 23337 \| IZALGI | 1 (33.33) | 1 (33.33) | 0 (0.00) |  |  |
| Tablet Dose (mg) Antalgic 2 |  | N=2 | n=2 | n=0 | INVALID | . |
|  | Mean (± SD) | 750.00 (± 353.55) | 750.00 (± 353.55) | . (± .) |  |  |
|  | Median (Q1;Q3) | 750.00 (500.00 ; 1000.00) | 750.00 (500.00 ; 1000.00) | . (. ; .) |  |  |
|  | Median (min;max) | 750.00 (500.00 ; 1000.00) | 750.00 (500.00 ; 1000.00) | . (. ; .) |  |  |
| No. of tablets per week Antalgic 2 |  | N=2 | n=2 | n=0 | INVALID | . |
|  | Mean (± SD) | 17.00 (± 4.24) | 17.00 (± 4.24) | . (± .) |  |  |
|  | Median (Q1;Q3) | 17.00 (14.00 ; 20.00) | 17.00 (14.00 ; 20.00) | . (. ; .) |  |  |
|  | Median (min;max) | 17.00 (14.00 ; 20.00) | 17.00 (14.00 ; 20.00) | . (. ; .) |  |  |
| Name Analgesic 3 (n(%col)) |  | N=1 | n=1 | n=0 | INVALID | . |
|  | 4548 \| DOLIPRANE (CP) | 1 (100.00) | 1 (100.00) | 0 (0.00) |  |  |
| Tablet dose (mg) Antalgic 3 |  | N=1 | n=1 | n=0 | INVALID | . |
|  | Mean (± SD) | 1000.00 (± .) | 1000.00 (± .) | . (± .) |  |  |
|  | Median (Q1;Q3) | 1000.00 (1000.00 ; 1000.00) | 1000.00 (1000.00 ; 1000.00) | . (. ; .) |  |  |
|  | Median (min;max) | 1000.00 (1000.00 ; 1000.00) | 1000.00 (1000.00 ; 1000.00) | . (. ; .) |  |  |
| No. tablets per week Analgesic 3 |  | N=1 | n=1 | n=0 | INVALID | . |
|  | Mean (± SD) | 2.00 (± .) | 2.00 (± .) | . (± .) |  |  |
|  | Median (Q1;Q3) | 2.00 (2.00 ; 2.00) | 2.00 (2.00 ; 2.00) | . (. ; .) |  |  |
|  | Median (min;max) | 2.00 (2.00 ; 2.00) | 2.00 (2.00 ; 2.00) | . (. ; .) |  |  |

Classement des Antalgiques à V0 par palier :

|  | | | **Group Name** | |  | |
| --- | --- | --- | --- | --- | --- | --- |
| **variable** | **Details** | **total population** | **Program MBSR** | **Classic follow-up and general advice** | **Test** | **p** |
| V0_level (n(% col)) |  | N=40 | n=20 | n=20 | FISHER | 0.010 |
|  | At least one level 1 | 6 (15.00) | 0 (0.00) | 6 (30.00) |  |  |
|  | At least one level 2 | 2 (5.00) | 2 (10.00) | 0 (0.00) |  |  |
|  | Nothing | 32 (80.00) | 18 (90.00) | 14 (70.00) |  |  |

#### Randomisation

|  | | | **Group Name** | |  | |
| --- | --- | --- | --- | --- | --- | --- |
| **variable** | **Details** | **total population** | **Program MBSR** | **Classic follow-up and general advice** | **Test** | **p** |
| Most symptomatic joint location (n(%col)) |  | N=40 | n=20 | n=20 | FISHER | 1.000 |
|  | Hip | 4 (10.00) | 2 (10.00) | 2 (10.00) |  |  |
|  | Knee | 36 (90.00) | 18 (90.00) | 18 (90.00) |  |  |
| Session 1 MBSR Y/N 1 (n(%col)) |  | N=20 | n=20 | n=0 | INVALID | . |
|  | No | 1 (5.00) | 1 (5.00) | 0 (0.00) |  |  |
|  | Yes | 19 (95.00) | 19 (95.00) | 0 (0.00) |  |  |
| MBSR 1 not done Reason 1 (n(%col)) |  | N=1 | n=1 | n=0 | INVALID | . |
|  | CUSTODY OF HER LITTLE GIRL | 1 (100.00) | 1 (100.00) | 0 (0.00) |  |  |
| Session 2 MBSR Y/N 2 (n(%col)) |  | N=20 | n=20 | n=0 | INVALID | . |
|  | No | 1 (5.00) | 1 (5.00) | 0 (0.00) |  |  |
|  | Yes | 19 (95.00) | 19 (95.00) | 0 (0.00) |  |  |
| MBSR 2 not done Reason 2 (n(%col)) |  | N=1 | n=1 | n=0 | INVALID | . |
|  | NO TIME - RESUMPTION OF ACTIVITY | 1 (100.00) | 1 (100.00) | 0 (0.00) |  |  |
| Session 3 MBSR Y/N 3 (n(%col)) |  | N=20 | n=20 | n=0 | INVALID | . |
|  | No | 2 (10.00) | 2 (10.00) | 0 (0.00) |  |  |
|  | Yes | 18 (90.00) | 18 (90.00) | 0 (0.00) |  |  |
| MBSR 3 not done Reason 3 (n(%col)) |  | N=2 | n=2 | n=0 | INVALID | . |
|  | NO TIME-RESUME ACTIVITY | 1 (50.00) | 1 (50.00) | 0 (0.00) |  |  |
|  | STAY OUTSIDE OF MONTPELLIER ALREADY SCHEDULED IN ADVANCE | 1 (50.00) | 1 (50.00) | 0 (0.00) |  |  |
| Session 4 MBSR Y/N 4 (n(%col)) | Hip | N=20 | n=20 | n=0 | INVALID | . |
|  | Knee | 4 (20.00) | 4 (20.00) | 0 (0.00) |  |  |
|  |  | 16 (80.00) | 16 (80.00) | 0 (0.00) |  |  |
| MBSR 4 not done Reason 4 (n(%col)) | No | N=4 | n=4 | n=0 | INVALID | . |
|  | Yes | 1 (25.00) | 1 (25.00) | 0 (0.00) |  |  |
|  |  | 1 (25.00) | 1 (25.00) | 0 (0.00) |  |  |
|  | CUSTODIAL OF HER GRANDDAUGHTER | 1 (25.00) | 1 (25.00) | 0 (0.00) |  |  |
|  |  | 1 (25.00) | 1 (25.00) | 0 (0.00) |  |  |
| Session 5 MBSR Y/N 5 (n(%col)) | No | N=20 | n=20 | n=0 | INVALID | . |
|  | Yes | 2 (10.00) | 2 (10.00) | 0 (0.00) |  |  |
|  |  | 18 (90.00) | 18 (90.00) | 0 (0.00) |  |  |
| MBSR 5 not done Reason 5 (n(%col)) | NO TIME - RESUMPTION OF ACTIVITY | N=2 | n=2 | n=0 | INVALID | . |
|  |  | 1 (50.00) | 1 (50.00) | 0 (0.00) |  |  |
|  | No | 1 (50.00) | 1 (50.00) | 0 (0.00) |  |  |
| Session 6 MBSR Y/N 6 (n(%col)) | Yes | N=20 | n=20 | n=0 | INVALID | . |
|  |  | 2 (10.00) | 2 (10.00) | 0 (0.00) |  |  |
|  | NO TIME-RESUME ACTIVITY | 18 (90.00) | 18 (90.00) | 0 (0.00) |  |  |
| MBSR 6 not done Reason 6 (n(%col)) | STAY OUTSIDE OF MONTPELLIER ALREADY SCHEDULED IN ADVANCE | N=2 | n=2 | n=0 | INVALID | . |
|  | Hip | 1 (50.00) | 1 (50.00) | 0 (0.00) |  |  |
|  | Knee | 1 (50.00) | 1 (50.00) | 0 (0.00) |  |  |
| Session 7 MBSR Y/N 7 (n(%col)) |  | N=20 | n=20 | n=0 | INVALID | . |
|  | No | 6 (30.00) | 6 (30.00) | 0 (0.00) |  |  |
|  | Yes | 14 (70.00) | 14 (70.00) | 0 (0.00) |  |  |
| MBSR 7 not done Reason 7 (n(%col)) |  | N=6 | n=6 | n=0 | INVALID | . |
|  | BABYSITTING HIS LITTLE GIRL | 1 (16.67) | 1 (16.67) | 0 (0.00) |  |  |
|  |  | 1 (16.67) | 1 (16.67) | 0 (0.00) |  |  |
|  | STAFF | 3 (50.00) | 3 (50.00) | 0 (0.00) |  |  |
|  | TRAVEL PROGRAM. INITIALLY THE SESSIONS WERE TO END BEFORE | 1 (16.67) | 1 (16.67) | 0 (0.00) |  |  |
| Session 8 MBSR Y/N 8 (n(%col)) |  | N=20 | n=20 | n=0 | INVALID | . |
|  | No | 4 (20.00) | 4 (20.00) | 0 (0.00) |  |  |
|  | Yes | 16 (80.00) | 16 (80.00) | 0 (0.00) |  |  |
| MBSR 8 not done Reason 8 (n(%col)) |  | N=4 | n=4 | n=0 | INVALID | . |
|  | SICK | 1 (25.00) | 1 (25.00) | 0 (0.00) |  |  |
|  | NOT COMMUNICATED | 1 (25.00) | 1 (25.00) | 0 (0.00) |  |  |
|  | NO TIME TO RESUME ACTIVITY | 1 (25.00) | 1 (25.00) | 0 (0.00) |  |  |
|  | STAFF | 1 (25.00) | 1 (25.00) | 0 (0.00) |  |  |
| AE Y/N (n(%col)) |  | N=20 | n=20 | n=0 | INVALID | . |
|  | No | 17 (85.00) | 17 (85.00) | 0 (0.00) |  |  |
|  | Yes | 3 (15.00) | 3 (15.00) | 0 (0.00) |  |  |
| Date of last group session (n(%col)) |  | N=20 | n=20 | n=0 | INVALID | . |
|  | 04/03/2019 | 12 (60.00) | 12 (60.00) | 0 (0.00) |  |  |
|  | 20/11/2018 | 8 (40.00) | 8 (40.00) | 0 (0.00) |  |  |
| Date of last group session (n(%col)) |  | N=20 | n=20 | n=0 | INVALID | . |
|  | 20/11/18 | 8 (40.00) | 8 (40.00) | 0 (0.00) |  |  |
|  | 04/03/19 | 12 (60.00) | 12 (60.00) | 0 (0.00) |  |  |
| F/NF satisfaction (n(%col)) |  | N=20 | n=20 | n=0 | INVALID | . |
|  | No | 2 (10.00) | 2 (10.00) | 0 (0.00) |  |  |
|  | Yes | 18 (90.00) | 18 (90.00) | 0 (0.00) |  |  |
| Satisfaction score (n(%col)) |  | N=18 | n=18 | n=0 | INVALID | . |
|  | 8 | 1 (5.56) | 1 (5.56) | 0 (0.00) |  |  |
|  | 9 | 5 (27.78) | 5 (27.78) | 0 (0.00) |  |  |
|  | 10 | 12 (66.67) | 12 (66.67) | 0 (0.00) |  |  |
| Satisfaction not done Reason (n(%col)) |  | N=2 | n=2 | n=0 | INVALID | . |
|  | ATTENDED ONLY 3 SESSIONS, NOT ENOUGH FOR EVALUATION | 1 (50.00) | 1 (50.00) | 0 (0.00) |  |  |
|  | JUST 1 SESSION DONE | 1 (50.00) | 1 (50.00) | 0 (0.00) |  |  |

|  | | | **Group Name** | |  | |
| --- | --- | --- | --- | --- | --- | --- |
| **variable** | **Details** | **total population** | **Program MBSR** | **Classic follow-up and general advice** | **Test** | **p** |
| Nb of MBSR sessions (n(% col)) |  | N=20 | n=20 | n=0 | INVALID | . |
|  | 1 | 1 (5.00) | 1 (5.00) | 0 (0.00) |  |  |
|  | 3 | 1 (5.00) | 1 (5.00) | 0 (0.00) |  |  |
|  | 6 | 1 (5.00) | 1 (5.00) | 0 (0.00) |  |  |
|  | 7 | 8 (40.00) | 8 (40.00) | 0 (0.00) |  |  |
|  | 8 | 9 (45.00) | 9 (45.00) | 0 (0.00) |  |  |
| Nb of MBSR sessions >= 6 (n(% col)) |  | N=20 | n=20 | n=0 | INVALID | . |
|  | No | 2 (10.00) | 2 (10.00) | 0 (0.00) |  |  |
|  | Yes | 18 (90.00) | 18 (90.00) | 0 (0.00) |  |  |

#### Activité de la maladie (V0)

|  | | | **Group Name** | |  | |
| --- | --- | --- | --- | --- | --- | --- |
| **variable** | **Details** | **total population** | **Program MBSR** | **Classic follow-up and general advice** | **Test** | **p** |
| VAS: physician's assessment of illness (F/NF) (n(%col)) |  | N=40 | n=20 | n=20 | INVALID | . |
|  | Done | 40 (100.00) | 20 (100.00) | 20 (100.00) |  |  |
| VAS: physician's disease assessment score |  | N=40 | n=20 | n=20 | WMW | 0.054 |
|  | Mean (± SD) | 56.63 (± 9.50) | 59.50 (± 9.85) | 53.75 (± 8.41) |  |  |
|  | Median (Q1;Q3) | 60.00 (50.00 ; 60.00) | 60.00 (55.00 ; 60.00) | 50.00 (50.00 ; 60.00) |  |  |
|  | Median (min;max) | 60.00 (40.00 ; 80.00) | 60.00 (40.00 ; 80.00) | 50.00 (40.00 ; 70.00) |  |  |

#### Auto-questionnaire (V0)

|  | | | **Group Name** | |  | |
| --- | --- | --- | --- | --- | --- | --- |
| **variable** | **Details** | **total population** | **Program MBSR** | **Classic follow-up and general advice** | **Test** | **p** |
| VAS: patient pain assessment (n(%col)) |  | N=40 | n=20 | n=20 | INVALID | . |
|  | Done | 40 (100.00) | 20 (100.00) | 20 (100.00) |  |  |
| VAS: patient's assessment of pain |  | N=40 | n=20 | n=20 | STUDENT | 0.309 |
|  | Mean (± SD) | 61.98 (± 12.43) | 64.00 (± 12.17) | 59.95 (± 12.68) |  |  |
|  | Median (Q1;Q3) | 61.50 (51.00 ; 72.00) | 61.50 (57.50 ; 73.00) | 57.50 (50.50 ; 71.50) |  |  |
|  | Median (min;max) | 61.50 (41.00 ; 94.00) | 61.50 (47.00 ; 94.00) | 57.50 (41.00 ; 81.00) |  |  |
| VAS: patient assessment of disease activity (n(%col)) |  | N=40 | n=20 | n=20 | INVALID | . |
|  | Done | 40 (100.00) | 20 (100.00) | 20 (100.00) |  |  |
| VAS: patient's assessment of disease activity |  | N=40 | n=20 | n=20 | WMW | 0.137 |
|  | Average (± SD) | 59.00 (± 18.02) | 63.40 (± 18.91) | 54.60 (± 16.37) |  |  |
|  | Median (Q1;Q3) | 60.50 (51.00 ; 71.00) | 62.50 (51.50 ; 78.50) | 56.00 (51.00 ; 65.50) |  |  |
|  | Median (min;max) | 60.50 (8.00 ; 91.00) | 62.50 (25.00 ; 91.00) | 56.00 (8.00 ; 74.00) |  |  |
| VAS: patient health assessment F/NF (n(%col)) |  | N=40 | n=20 | n=20 | INVALID | . |
|  | Done | 40 (100.00) | 20 (100.00) | 20 (100.00) |  |  |
| VAS: patient health assessment score |  | N=40 | n=20 | n=20 | STUDENT | 0.596 |
|  | Mean (± SD) | 57.85 (± 23.43) | 59.85 (± 22.88) | 55.85 (± 24.38) |  |  |
|  | Median (Q1;Q3) | 61.00 (39.50 ; 75.50) | 60.50 (43.00 ; 78.00) | 65.00 (31.00 ; 74.00) |  |  |
|  | Median (min;max) | 61.00 (14.00 ; 100.00) | 60.50 (14.00 ; 100.00) | 65.00 (15.00 ; 95.00) |  |  |
| WOMAC F/NF (n(%col)) |  | N=40 | n=20 | n=20 | INVALID | . |
|  | Done | 40 (100.00) | 20 (100.00) | 20 (100.00) |  |  |
| SF36 F/NF (n(%col)) |  | N=40 | n=20 | n=20 | INVALID | . |
|  | Done | 40 (100.00) | 20 (100.00) | 20 (100.00) |  |  |
| HAD F/NF (n(%col)) |  | N=40 | n=20 | n=20 | INVALID | . |
|  | Done | 40 (100.00) | 20 (100.00) | 20 (100.00) |  |  |
| FFMQ F/NF (n(%col)) |  | N=40 | n=20 | n=20 | INVALID | . |
|  | Done | 40 (100.00) | 20 (100.00) | 20 (100.00) |  |  |
| Meditation practice Y/N (n(%col)) |  | N=7 | n=6 | n=1 | FISHER | 1.000 |
|  | Not Done | 3 (42.86) | 3 (50.00) | 0 (0.00) |  |  |
|  | Done | 4 (57.14) | 3 (50.00) | 1 (100.00) |  |  |
| Reason for not practicing meditation daily (n(%col)) |  | N=2 | n=2 | n=0 | INVALID | . |
|  | DM | 1 (50.00) | 1 (50.00) | 0 (0.00) |  |  |
|  | NON CONNU | 1 (50.00) | 1 (50.00) | 0 (0.00) |  |  |

#### WOMAC Index (V0)

|  | | | **Group Name** | |  | |
| --- | --- | --- | --- | --- | --- | --- |
| **variable** | **Details** | **total population** | **Program MBSR** | **Classic follow-up and general advice** | **test** | **p** |
| Womac : Score pain |  | N=36 | n=17 | n=19 | STUDENT | 0.152 |
|  | Mean (± SD) | 51.53 (± 15.98) | 55.59 (± 16.67) | 47.89 (± 14.84) |  |  |
|  | Median (Q1;Q3) | 50.00 (40.00 ; 65.00) | 60.00 (40.00 ; 65.00) | 50.00 (35.00 ; 60.00) |  |  |
|  | Median (min;max) | 50.00 (20.00 ; 95.00) | 60.00 (30.00 ; 95.00) | 50.00 (20.00 ; 75.00) |  |  |
| WOMAC : Score function |  | N=36 | n=17 | n=19 | STUDENT | 0.544 |
|  | Mean (± SD) | 47.14 (± 16.71) | 48.96 (± 19.24) | 45.51 (± 14.43) |  |  |
|  | Median (Q1;Q3) | 48.53 (37.50 ; 58.09) | 51.47 (42.65 ; 58.82) | 48.53 (36.76 ; 57.35) |  |  |
|  | Median (min;max) | 48.53 (7.35 ; 79.41) | 51.47 (7.35 ; 79.41) | 48.53 (19.12 ; 69.12) |  |  |
| WOMAC : Score Stiffness |  | N=40 | n=20 | n=20 | STUDENT | 0.283 |
|  | Mean (± SD) | 55.94 (± 20.01) | 59.38 (± 17.62) | 52.50 (± 22.06) |  |  |
|  | Median (Q1;Q3) | 50.00 (50.00 ; 75.00) | 56.25 (50.00 ; 75.00) | 50.00 (43.75 ; 68.75) |  |  |
|  | Median (min;max) | 50.00 (0.00 ; 100.00) | 56.25 (25.00 ; 87.50) | 50.00 (0.00 ; 100.00) |  |  |
| WOMAC : Score Total |  | N=33 | n=15 | n=18 | STUDENT | 0.206 |
|  | Mean (± SD) | 51.54 (± 14.43) | 55.05 (± 13.57) | 48.61 (± 14.84) |  |  |
|  | Median (Q1;Q3) | 50.00 (39.07 ; 62.45) | 55.78 (43.14 ; 66.27) | 46.30 (35.59 ; 61.81) |  |  |
|  | Median (min;max) | 50.00 (24.71 ; 75.64) | 55.78 (33.92 ; 75.64) | 46.30 (24.71 ; 73.14) |  |  |

#### SF-36 health status questionnaire without inconsistencies (V0)

|  | | | **Group Name** | |  | |
| --- | --- | --- | --- | --- | --- | --- |
| **variable** | **Details** | **total population** | **Program MBSR** | **Classic follow-up and general advice** | **test** | **p** |
| Score : Physical Activity(i) |  | N=39 | n=20 | n=19 | STUDENT | 0.770 |
|  | Mean (± SD) | 51.20 (± 22.98) | 50.13 (± 22.01) | 52.32 (± 24.52) |  |  |
|  | Median (Q1;Q3) | 45.00 (35.00 ; 70.00) | 45.00 (35.00 ; 67.50) | 45.00 (40.00 ; 70.00) |  |  |
|  | Median (min;max) | 45.00 (5.00 ; 95.00) | 45.00 (5.00 ; 85.00) | 45.00 (10.00 ; 95.00) |  |  |
| Score : Limitations due to physical condition(i) |  | N=39 | n=20 | n=19 | WMW | 0.405 |
|  | Mean (± SD) | 35.90 (± 37.08) | 41.25 (± 39.96) | 30.26 (± 33.93) |  |  |
|  | Median (Q1;Q3) | 25.00 (0.00 ; 75.00) | 25.00 (0.00 ; 75.00) | 25.00 (0.00 ; 75.00) |  |  |
|  | Median (min;max) | 25.00 (0.00 ; 100.00) | 25.00 (0.00 ; 100.00) | 25.00 (0.00 ; 100.00) |  |  |
| Score : Limitations due to psychological state(i) |  | N=40 | n=20 | n=20 | WMW | 0.173 |
|  | Mean (± SD) | 55.83 (± 42.29) | 65.00 (± 41.15) | 46.67 (± 42.44) |  |  |
|  | Median (Q1;Q3) | 66.67 (0.00 ; 100.00) | 83.33 (33.33 ; 100.00) | 33.33 (0.00 ; 100.00) |  |  |
|  | Median (min;max) | 66.67 (0.00 ; 100.00) | 83.33 (0.00 ; 100.00) | 33.33 (0.00 ; 100.00) |  |  |
| Score : Life and relationship with others(i) |  | N=40 | n=20 | n=20 | STUDENT | 0.873 |
|  | Mean (± SD) | 60.00 (± 24.22) | 59.38 (± 26.56) | 60.63 (± 22.31) |  |  |
|  | Median (Q1;Q3) | 62.50 (50.00 ; 75.00) | 50.00 (43.75 ; 87.50) | 62.50 (50.00 ; 68.75) |  |  |
|  | Median (min;max) | 62.50 (0.00 ; 100.00) | 50.00 (0.00 ; 100.00) | 62.50 (12.50 ; 100.00) |  |  |
| Score : Physical pain(i) |  | N=40 | n=20 | n=20 | WMW | 0.591 |
|  | Mean (± SD) | 36.70 (± 15.68) | 34.95 (± 18.79) | 38.45 (± 12.05) |  |  |
|  | Median (Q1;Q3) | 41.00 (22.00 ; 41.00) | 36.50 (22.00 ; 51.00) | 41.00 (31.50 ; 41.00) |  |  |
|  | Médiane (min;max) | 41.00 (0.00 ; 72.00) | 36.50 (0.00 ; 72.00) | 41.00 (22.00 ; 62.00) |  |  |
| Score : Psychological health(i) |  | N=40 | n=20 | n=20 | STUDENT | 0.146 |
|  | Mean (± SD) | 61.13 (± 16.98) | 65.05 (± 15.01) | 57.20 (± 18.27) |  |  |
|  | Median (Q1;Q3) | 62.00 (48.00 ; 76.00) | 66.50 (52.00 ; 76.00) | 58.00 (44.00 ; 72.00) |  |  |
|  | Median (min;max) | 62.00 (24.00 ; 92.00) | 66.50 (32.00 ; 92.00) | 58.00 (24.00 ; 84.00) |  |  |
| Score: Vitality(i) |  | N=40 | n=20 | n=20 | WMW | 0.354 |
|  | Mean (± SD) | 48.25 (± 20.96) | 53.00 (± 22.38) | 43.50 (± 18.79) |  |  |
|  | Median (Q1;Q3) | 45.00 (40.00 ; 62.50) | 47.50 (35.00 ; 72.50) | 45.00 (40.00 ; 52.50) |  |  |
|  | Median (min;max) | 45.00 (0.00 ; 100.00) | 47.50 (20.00 ; 100.00) | 45.00 (0.00 ; 75.00) |  |  |
| Score:Perceived health(i) |  | N=40 | n=20 | n=20 | WMW | 0.578 |
|  | Mean (± SD) | 56.38 (± 18.36) | 55.80 (± 16.18) | 56.95 (± 20.72) |  |  |
|  | Median (Q1;Q3) | 57.00 (42.88 ; 69.50) | 51.00 (41.88 ; 69.50) | 62.00 (44.50 ; 69.50) |  |  |
|  | Median (min;max) | 57.00 (15.00 ; 87.00) | 51.00 (37.00 ; 87.00) | 62.00 (15.00 ; 87.00) |  |  |
| Aggregate physical score(i) |  | N=38 | n=20 | n=18 | STUDENT | 0.625 |
|  | Mean (± SD) | 35.45 (± 8.04) | 34.83 (± 8.06) | 36.14 (± 8.19) |  |  |
|  | Median (Q1;Q3) | 35.68 (28.76 ; 41.51) | 36.13 (29.82 ; 41.22) | 35.68 (28.67 ; 42.31) |  |  |
|  | Median (min;max) | 35.68 (19.97 ; 55.12) | 36.13 (19.97 ; 48.99) | 35.68 (27.03 ; 55.12) |  |  |
| Aggregate psychic score(i) |  | N=38 | n=20 | n=18 | STUDENT | 0.114 |
|  | Mean (± SD) | 45.42 (± 11.03) | 48.12 (± 9.55) | 42.43 (± 12.04) |  |  |
|  | Median (Q1;Q3) | 47.53 (37.35 ; 56.55) | 47.82 (43.19 ; 57.23) | 38.30 (32.56 ; 52.43) |  |  |
|  | Median (min;max) | 47.53 (25.87 ; 64.09) | 47.82 (28.02 ; 60.99) | 38.30 (25.87 ; 64.09) |  |  |
| Sum of the scores of the 8 dim of the SF36(/800)(i) |  | N=38 | n=20 | n=18 | STUDENT | 0.354 |
|  | Mean (± SD) | 402.46 (± 152.75) | 424.55 (± 151.39) | 377.92 (± 154.78) |  |  |
|  | Median (Q1;Q3) | 397.54 (249.50 ; 521.50) | 422.42 (332.83 ; 541.00) | 383.60 (249.33 ; 517.50) |  |  |
|  | Median (min;max) | 397.54 (138.50 ; 711.00) | 422.42 (144.75 ; 711.00) | 383.60 (138.50 ; 677.00) |  |  |
| Average of the scores of the 8 dim of the SF36 (i) |  | N=38 | n=20 | n=18 | STUDENT | 0.354 |
|  | Mean (± SD) | 50.31 (± 19.09) | 53.07 (± 18.92) | 47.24 (± 19.35) |  |  |
|  | Median (Q1;Q3) | 49.69 (31.19 ; 65.19) | 52.80 (41.60 ; 67.63) | 47.95 (31.17 ; 64.69) |  |  |
|  | Median (min;max) | 49.69 (17.31 ; 88.88) | 52.80 (18.09 ; 88.88) | 47.95 (17.31 ; 84.63) |  |  |

#### HADS (V0)

|  | | | **Group Name** | |  | |
| --- | --- | --- | --- | --- | --- | --- |
| **variable** | **Details** | **total population** | **Program MBSR** | **Classic follow-up and general advice** | **test** | **p** |
| HADS : Score Anxiety |  | N=40 | n=20 | n=20 | STUDENT | 0.406 |
|  | Mean (± SD) | 7.80 (± 4.12) | 7.25 (± 3.95) | 8.35 (± 4.32) |  |  |
|  | Median (Q1;Q3) | 7.00 (5.00 ; 11.00) | 6.00 (4.50 ; 10.00) | 8.00 (5.50 ; 11.50) |  |  |
|  | Median (min;max) | 7.00 (1.00 ; 17.00) | 6.00 (1.00 ; 15.00) | 8.00 (1.00 ; 17.00) |  |  |
| HADS : Score Depression |  | N=40 | n=20 | n=20 | WMW | 0.281 |
|  | Mean (± SD) | 4.68 (± 3.44) | 4.10 (± 3.31) | 5.25 (± 3.57) |  |  |
|  | Median (Q1;Q3) | 4.00 (2.00 ; 8.00) | 3.00 (1.50 ; 6.00) | 4.00 (2.00 ; 8.50) |  |  |
|  | Median (min;max) | 4.00 (0.00 ; 12.00) | 3.00 (1.00 ; 11.00) | 4.00 (0.00 ; 12.00) |  |  |
| HADS : Score Total |  | N=40 | n=20 | n=20 | STUDENT | 0.288 |
|  | Mean (± SD) | 12.48 (± 6.61) | 11.35 (± 6.60) | 13.60 (± 6.60) |  |  |
|  | Median (Q1;Q3) | 11.50 (8.00 ; 17.50) | 9.50 (7.00 ; 14.00) | 13.00 (8.50 ; 18.50) |  |  |
|  | Median (min;max) | 11.50 (2.00 ; 27.00) | 9.50 (2.00 ; 25.00) | 13.00 (3.00 ; 27.00) |  |  |

#### FFMQ (V0)

|  | | | **Group Name** | |  | |
| --- | --- | --- | --- | --- | --- | --- |
| **variable** | **Details** | **total population** | **Program MBSR** | **Classic follow-up and general advice** | **test** | **p** |
| FFMQ : Observation Factor |  | N=39 | n=19 | n=20 | STUDENT | 0.202 |
|  | Mean (± SD) | 28.05 (± 5.74) | 29.26 (± 5.51) | 26.90 (± 5.85) |  |  |
|  | Median (Q1;Q3) | 29.00 (25.00 ; 33.00) | 29.00 (25.00 ; 34.00) | 26.50 (23.00 ; 31.50) |  |  |
|  | Median (min;max) | 29.00 (14.00 ; 36.00) | 29.00 (15.00 ; 36.00) | 26.50 (14.00 ; 36.00) |  |  |
| FFMQ: Description Factor Experience |  | N=39 | n=20 | n=19 | STUDENT | 0.747 |
|  | Mean (± SD) | 28.64 (± 6.03) | 28.95 (± 5.40) | 28.32 (± 6.76) |  |  |
|  | Median (Q1;Q3) | 30.00 (25.00 ; 34.00) | 30.00 (26.00 ; 31.50) | 30.00 (24.00 ; 34.00) |  |  |
|  | Median (min;max) | 30.00 (15.00 ; 39.00) | 30.00 (15.00 ; 37.00) | 30.00 (16.00 ; 39.00) |  |  |
| FFMQ : Factor Full consciousness |  | N=38 | n=19 | n=19 | STUDENT | 0.707 |
|  | Mean (± SD) | 28.21 (± 5.92) | 28.58 (± 6.28) | 27.84 (± 5.69) |  |  |
|  | Median (Q1;Q3) | 28.50 (23.00 ; 32.00) | 29.00 (23.00 ; 32.00) | 28.00 (23.00 ; 34.00) |  |  |
|  | Median (min;max) | 28.50 (18.00 ; 38.00) | 29.00 (18.00 ; 38.00) | 28.00 (18.00 ; 37.00) |  |  |
| FFMQ: Private Evt Factor |  | N=39 | n=19 | n=20 | STUDENT | 0.113 |
|  | Mean (± SD) | 20.03 (± 4.53) | 18.84 (± 4.97) | 21.15 (± 3.87) |  |  |
|  | Median (Q1;Q3) | 20.00 (17.00 ; 23.00) | 18.00 (16.00 ; 23.00) | 21.00 (20.00 ; 23.50) |  |  |
|  | Median (min;max) | 20.00 (8.00 ; 27.00) | 18.00 (8.00 ; 27.00) | 21.00 (12.00 ; 27.00) |  |  |
| FFMQ: Non-judgment Factor |  | N=40 | n=20 | n=20 | STUDENT | 0.410 |
|  | Mean (± SD) | 27.13 (± 6.61) | 28.00 (± 5.78) | 26.25 (± 7.40) |  |  |
|  | Median (Q1;Q3) | 27.00 (21.00 ; 31.00) | 28.00 (23.50 ; 31.00) | 26.00 (20.00 ; 31.00) |  |  |
|  | Median (min;max) | 27.00 (14.00 ; 40.00) | 28.00 (20.00 ; 40.00) | 26.00 (14.00 ; 40.00) |  |  |
| FFMQ : Mindfulness |  | N=36 | n=18 | n=18 | STUDENT | 0.742 |
|  | Mean (± SD) | 132.19 (± 19.31) | 133.28 (± 17.00) | 131.11 (± 21.83) |  |  |
|  | Median (Q1;Q3) | 131.00 (117.00 ; 149.50) | 135.00 (118.00 ; 149.00) | 127.00 (115.00 ; 151.00) |  |  |
|  | Median (min;max) | 131.00 (86.00 ; 162.00) | 135.00 (104.00 ; 159.00) | 127.00 (86.00 ; 162.00) |  |  |

### VISIT V1- END OF PROGRAM (+/-15 DAYS)

|  | | | **Group Name** | |  | |
| --- | --- | --- | --- | --- | --- | --- |
| **variable** | **Details** | **total population** | **Program MBSR** | **Classic follow-up and general advice** | **Test** | **p** |
| Follow-up done Y/N (n(%col)) |  | N=40 | n=20 | n=20 | FISHER | 0.044 |
|  | No | 8 (20.00) | 1 (5.00) | 7 (35.00) |  |  |
|  | Yes | 32 (80.00) | 19 (95.00) | 13 (65.00) |  |  |
| Visit not made: Questionnaires sent Y/N (n(%col)) |  | N=4 | n=1 | n=3 | INVALID | . |
|  | Yes | 4 (100.00) | 1 (100.00) | 3 (100.00) |  |  |
| Reason Not Done (n(%col)) |  | N=8 | n=1 | n=7 | INVALID | . |
|  | Other reason | 8 (100.00) | 1 (100.00) | 7 (100.00) |  |  |
| Other Reason Not Done (n(%col)) |  | N=8 | n=1 | n=7 | FISHER | 1.000 |
|  | ABSENT | 1 (12.50) | 0 (0.00) | 1 (14.29) |  |  |
|  | SICK GRIPPE | 1 (12.50) | 1 (100.00) | 0 (0.00) |  |  |
|  | DOES NOT WISH TO CONTINUE | 1 (12.50) | 0 (0.00) | 1 (14.29) |  |  |
|  | DOES NOT WANT TO COME ANYMORE, NO MORE PAIN DOES NOT FEEL CONCERNED ANYMORE | 1 (12.50) | 0 (0.00) | 1 (14.29) |  |  |
|  | PATIENT DID NOT COME | 1 (12.50) | 0 (0.00) | 1 (14.29) |  |  |
|  | PATIENT DID NOT COME | 1 (12.50) | 0 (0.00) | 1 (14.29) |  |  |
|  | LOST TO VIEW | 1 (12.50) | 0 (0.00) | 1 (14.29) |  |  |
|  | APPOINTMENT CANCELLED | 1 (12.50) | 0 (0.00) | 1 (14.29) |  |  |
| Alert AE (n(%col)) |  | N=2 | n=1 | n=1 | INVALID | . |
|  | Remember to enter the EvI/EvIG at the end of the booklet!!! | 2 (100.00) | 1 (100.00) | 1 (100.00) |  |  |
| Ev Undesirable Y/N (n(%col)) |  | N=35 | n=20 | n=15 | FISHER | 1.000 |
|  | No | 33 (94.29) | 19 (95.00) | 14 (93.33) |  |  |
|  | Yes | 2 (5.71) | 1 (5.00) | 1 (6.67) |  |  |

#### Clinical examination (V1)

|  | | | **Group Name** | |  | |
| --- | --- | --- | --- | --- | --- | --- |
| **variable** | **Details** | **total population** | **Program MBSR** | **Classic follow-up and general advice** | **test** | **p** |
| Weight |  | N=32 | n=19 | n=13 | STUDENT | 0.31 |
|  | Mean (± SD) | 74.06 (± 19.45) | 71.11 (± 20.19) | 78.38 (± 18.22) |  |  |
|  | Median (Q1;Q3) | 70.50 (59.40 ; 85.00) | 68.00 (54.00 ; 85.00) | 77.90 (68.00 ; 85.00) |  |  |
|  | Median (min;max) | 70.50 (47.30 ; 125.00) | 68.00 (47.30 ; 122.00) | 77.90 (52.00 ; 125.00) |  |  |
| Hight |  | N=32 | n=19 | n=13 | WMW | 0.97 |
|  | Mean (± SD) | 165.03 (± 8.75) | 165.53 (± 9.97) | 164.31 (± 6.92) |  |  |
|  | Median (Q1;Q3) | 163.50 (159.00 ; 170.00) | 162.00 (159.00 ; 170.00) | 166.00 (159.00 ; 170.00) |  |  |
|  | Median (min;max) | 163.50 (153.00 ; 191.00) | 162.00 (153.00 ; 191.00) | 166.00 (154.00 ; 175.00) |  |  |
| BMI |  | N=32 | n=19 | n=13 | STUDENT | 0.16 |
|  | Mean (± SD) | 27.17 (± 6.83) | 25.74 (± 5.97) | 29.25 (± 7.70) |  |  |
|  | Median (Q1;Q3) | 27.10 (21.77 ; 30.05) | 24.98 (20.70 ; 29.72) | 27.24 (26.40 ; 31.89) |  |  |
|  | Median (min;max) | 27.10 (17.72 ; 49.44) | 24.98 (17.72 ; 41.91) | 27.24 (17.99 ; 49.44) |  |  |
| BP Systole |  | N=32 | n=19 | n=13 | STUDENT | 0.16 |
|  | Mean (± SD) | 131.78 (± 17.95) | 128.05 (± 14.66) | 137.23 (± 21.34) |  |  |
|  | Median (Q1;Q3) | 131.00 (122.00 ; 142.50) | 131.00 (117.00 ; 135.00) | 135.00 (122.00 ; 156.00) |  |  |
|  | Median (min;max) | 131.00 (97.00 ; 168.00) | 131.00 (97.00 ; 152.00) | 135.00 (103.00 ; 168.00) |  |  |

##### Examen clinique articulations

|  | | | **Group Name** | |  | |
| --- | --- | --- | --- | --- | --- | --- |
| **variable** | **Details** | **total population** | **Program MBSR** | **Classic follow-up and general advice** | **Test** | **p** |
| Right knee pain Y/N (n(%col)) |  | N=32 | n=19 | n=13 | FISHER | 0.401 |
|  | No | 25 (78.13) | 16 (84.21) | 9 (69.23) |  |  |
|  | Yes | 7 (21.88) | 3 (15.79) | 4 (30.77) |  |  |
| Right knee effusion Y/N (n(%col)) |  | N=32 | n=19 | n=13 | FISHER | 1.000 |
|  | No | 30 (93.75) | 18 (94.74) | 12 (92.31) |  |  |
|  | Yes | 2 (6.25) | 1 (5.26) | 1 (7.69) |  |  |
| Patellofemoral syndrome right knee Y/N (n(%col)) |  | N=32 | n=19 | n=13 | FISHER | 0.401 |
|  | No | 25 (78.13) | 16 (84.21) | 9 (69.23) |  |  |
|  | Yes | 7 (21.88) | 3 (15.79) | 4 (30.77) |  |  |
| Flessum right knee Y/N (n(%col)) |  | N=32 | n=19 | n=13 | FISHER | 1.000 |
|  | No | 31 (96.88) | 18 (94.74) | 13 (100.00) |  |  |
|  | Yes | 1 (3.13) | 1 (5.26) | 0 (0.00) |  |  |
| Left knee pain Y/N (n(%col)) |  | N=32 | n=19 | n=13 | FISHER | 0.283 |
|  | No | 21 (65.63) | 14 (73.68) | 7 (53.85) |  |  |
|  | Yes | 11 (34.38) | 5 (26.32) | 6 (46.15) |  |  |
| Left knee effusion Y/N (n(%col)) |  | N=32 | n=19 | n=13 | FISHER | 1.000 |
|  | No | 30 (93.75) | 18 (94.74) | 12 (92.31) |  |  |
|  | Yes | 2 (6.25) | 1 (5.26) | 1 (7.69) |  |  |
| Patellofemoral syndrome left knee Y/N (n(%col)) |  | N=32 | n=19 | n=13 | FISHER | 0.684 |
|  | No | 24 (75.00) | 15 (78.95) | 9 (69.23) |  |  |
|  | Yes | 8 (25.00) | 4 (21.05) | 4 (30.77) |  |  |
| Flessum left knee Y/N (n(%col)) |  | N=32 | n=19 | n=13 | FISHER | 0.253 |
|  | No | 29 (90.63) | 16 (84.21) | 13 (100.00) |  |  |
|  | Yes | 3 (9.38) | 3 (15.79) | 0 (0.00) |  |  |
| Right hip pain Y/N (n(%col)) |  | N=32 | n=19 | n=13 | FISHER | 0.147 |
|  | No | 18 (56.25) | 8 (42.11) | 10 (76.92) |  |  |
|  | Yes | 2 (6.25) | 2 (10.53) | 0 (0.00) |  |  |
|  | NA | 12 (37.50) | 9 (47.37) | 3 (23.08) |  |  |
| Flessum right hip Y/N (n(%col)) |  | N=32 | n=19 | n=13 | CHI2 | 0.513 |
|  | No | 15 (46.88) | 8 (42.11) | 7 (53.85) |  |  |
|  | NA | 17 (53.13) | 11 (57.89) | 6 (46.15) |  |  |
| Flessum left hip Y/N (n(%col)) |  | N=32 | n=19 | n=13 | FISHER | 0.081 |
|  | No | 17 (53.13) | 7 (36.84) | 10 (76.92) |  |  |
|  | Yes | 3 (9.38) | 3 (15.79) | 0 (0.00) |  |  |
|  | NA | 12 (37.50) | 9 (47.37) | 3 (23.08) |  |  |
| Flessum left hip Y/N (n(%col)) |  | N=32 | n=19 | n=13 | CHI2 | 0.513 |
|  | No | 15 (46.88) | 8 (42.11) | 7 (53.85) |  |  |
|  | NA | 17 (53.13) | 11 (57.89) | 6 (46.15) |  |  |

|  | | | **Group Name** | |  | |
| --- | --- | --- | --- | --- | --- | --- |
| **variable** | **Details** | **total population** | **Program MBSR** | **Classic follow-up and general advice** | **Test** | **p** |
| Bilateral knee pain (n(% col)) |  | N=32 | n=19 | n=13 | FISHER | 0.374 |
|  | No | 27 (84.38) | 17 (89.47) | 10 (76.92) |  |  |
|  | Yes | 5 (15.63) | 2 (10.53) | 3 (23.08) |  |  |
| Unilateral knee pain (n(% col)) |  | N=32 | n=19 | n=13 | FISHER | 0.684 |
|  | No | 24 (75.00) | 15 (78.95) | 9 (69.23) |  |  |
|  | Yes | 8 (25.00) | 4 (21.05) | 4 (30.77) |  |  |
| Bilateral knee effusion (n(% col)) |  | N=32 | n=19 | n=13 | INVALID | . |
|  | No | 32 (100.00) | 19 (100.00) | 13 (100.00) |  |  |
| Unilateral knee effusion (n(% col)) |  | N=32 | n=19 | n=13 | FISHER | 1.000 |
|  | No | 28 (87.50) | 17 (89.47) | 11 (84.62) |  |  |
|  | Yes | 4 (12.50) | 2 (10.53) | 2 (15.38) |  |  |
| At least one knee effusion (n(% col)) |  | N=32 | n=19 | n=13 | FISHER | 1.000 |
|  | No | 28 (87.50) | 17 (89.47) | 11 (84.62) |  |  |
|  | Yes | 4 (12.50) | 2 (10.53) | 2 (15.38) |  |  |
| Bilateral patellofemoral syndrome (n(% col)) |  | N=32 | n=19 | n=13 | FISHER | 0.374 |
|  | No | 27 (84.38) | 17 (89.47) | 10 (76.92) |  |  |
|  | Yes | 5 (15.63) | 2 (10.53) | 3 (23.08) |  |  |
| Unilateral femoral patellar knee syndrome (n(% col)) |  | N=32 | n=19 | n=13 | FISHER | 1.000 |
|  | No | 27 (84.38) | 16 (84.21) | 11 (84.62) |  |  |
|  | Yes | 5 (15.63) | 3 (15.79) | 2 (15.38) |  |  |
| At least one femoro patellar knee syndrome (n(% col)) |  | N=32 | n=19 | n=13 | FISHER | 0.699 |
|  | No | 22 (68.75) | 14 (73.68) | 8 (61.54) |  |  |
|  | Yes | 10 (31.25) | 5 (26.32) | 5 (38.46) |  |  |
| Flessum knee bilateral (n(% col)) |  | N=32 | n=19 | n=13 | FISHER | 1.000 |
|  | No | 31 (96.88) | 18 (94.74) | 13 (100.00) |  |  |
|  | Yes | 1 (3.13) | 1 (5.26) | 0 (0.00) |  |  |
| Flessum knee unilateral (n(% col)) |  | N=32 | n=19 | n=13 | FISHER | 0.502 |
|  | No | 30 (93.75) | 17 (89.47) | 13 (100.00) |  |  |
|  | Yes | 2 (6.25) | 2 (10.53) | 0 (0.00) |  |  |
| At least one flessum knee (n(% col)) |  | N=32 | n=19 | n=13 | FISHER | 0.253 |
|  | No | 29 (90.63) | 16 (84.21) | 13 (100.00) |  |  |
|  | Yes | 3 (9.38) | 3 (15.79) | 0 (0.00) |  |  |
| Hip pain bilateral (n(% col)) |  | N=20 | n=10 | n=10 | FISHER | 0.474 |
|  | No | 18 (90.00) | 8 (80.00) | 10 (100.00) |  |  |
|  | Yes | 2 (10.00) | 2 (20.00) | 0 (0.00) |  |  |
| Unilateral hip pain (n(% col)) |  | N=20 | n=10 | n=10 | FISHER | 1.000 |
|  | No | 19 (95.00) | 9 (90.00) | 10 (100.00) |  |  |
|  | Yes | 1 (5.00) | 1 (10.00) | 0 (0.00) |  |  |
| Bilateral hip flessum (n(% col)) |  | N=15 | n=8 | n=7 | INVALID | . |
|  | No | 15 (100.00) | 8 (100.00) | 7 (100.00) |  |  |
| Unilateral hip flessum (n(% col)) |  | N=15 | n=8 | n=7 | INVALID | . |
|  | No | 15 (100.00) | 8 (100.00) | 7 (100.00) |  |  |
| At least one hip flessum (n(% col)) |  | N=15 | n=8 | n=7 | INVALID | . |
|  | No | 15 (100.00) | 8 (100.00) | 7 (100.00) |  |  |

|  | | | **Group Name** | |  | |
| --- | --- | --- | --- | --- | --- | --- |
| **variable** | **Details** | **total population** | **Program MBSR** | **Classic follow-up and general advice** | **Test** | **p** |
| At least one painful joint at V1 (n(%col)) |  | N=24 | n=12 | n=12 | CHI2 | 1.000 |
|  | No | 10 (41.67) | 5 (41.67) | 5 (41.67) |  |  |
|  | Yes | 14 (58.33) | 7 (58.33) | 7 (58.33) |  |  |

##### Mobilités en passif hanche

|  | | | **Group Name** | |  | |
| --- | --- | --- | --- | --- | --- | --- |
| **variable** | **Details** | **total population** | **Program MBSR** | **Classic follow-up and general advice** | **test** | **p** |
| Right hip flexion |  | N=32 | n=19 | n=13 | WMW | 0.772 |
|  | Mean (± SD) | 132.03 (± 21.02) | 133.16 (± 14.26) | 130.38 (± 28.83) |  |  |
|  | Median (Q1;Q3) | 135.00 (127.50 ; 142.50) | 135.00 (130.00 ; 140.00) | 140.00 (120.00 ; 145.00) |  |  |
|  | Median (min;max) | 135.00 (50.00 ; 160.00) | 135.00 (90.00 ; 160.00) | 140.00 (50.00 ; 160.00) |  |  |
| Right hip abduction |  | N=32 | n=19 | n=13 | WMW | 0.456 |
|  | Mean (± SD) | 56.41 (± 7.43) | 55.53 (± 7.05) | 57.69 (± 8.07) |  |  |
|  | Median (Q1;Q3) | 60.00 (50.00 ; 60.00) | 60.00 (45.00 ; 60.00) | 60.00 (50.00 ; 60.00) |  |  |
|  | Median (min;max) | 60.00 (45.00 ; 70.00) | 60.00 (45.00 ; 65.00) | 60.00 (45.00 ; 70.00) |  |  |
| Right hip internal rotation |  | N=32 | n=19 | n=13 | STUDENT | 0.547 |
|  | Mean (± SD) | 33.88 (± 11.91) | 34.95 (± 11.33) | 32.31 (± 13.01) |  |  |
|  | Median (Q1;Q3) | 30.00 (30.00 ; 45.00) | 30.00 (30.00 ; 45.00) | 35.00 (30.00 ; 45.00) |  |  |
|  | Median (min;max) | 30.00 (5.00 ; 55.00) | 30.00 (15.00 ; 55.00) | 35.00 (5.00 ; 50.00) |  |  |
| Right hip external rotation |  | N=32 | n=19 | n=13 | WMW | 0.344 |
|  | Mean (± SD) | 42.34 (± 13.38) | 40.26 (± 12.74) | 45.38 (± 14.21) |  |  |
|  | Median (Q1;Q3) | 45.00 (32.50 ; 47.50) | 45.00 (30.00 ; 45.00) | 45.00 (45.00 ; 60.00) |  |  |
|  | Median (min;max) | 45.00 (0.00 ; 65.00) | 45.00 (0.00 ; 60.00) | 45.00 (15.00 ; 65.00) |  |  |
| Left hip flexion ° |  | N=32 | n=19 | n=13 | WMW | 0.861 |
|  | Mean (± SD) | 132.66 (± 20.98) | 133.95 (± 14.39) | 130.77 (± 28.64) |  |  |
|  | Median (Q1;Q3) | 135.00 (130.00 ; 145.00) | 135.00 (130.00 ; 140.00) | 140.00 (120.00 ; 145.00) |  |  |
|  | Median (min;max) | 135.00 (50.00 ; 160.00) | 135.00 (90.00 ; 160.00) | 140.00 (50.00 ; 160.00) |  |  |
| Left hip abduction |  | N=32 | n=19 | n=13 | WMW | 0.233 |
|  | Mean (± SD) | 56.72 (± 7.36) | 55.26 (± 7.35) | 58.85 (± 7.12) |  |  |
|  | Median (Q1;Q3) | 60.00 (50.00 ; 60.00) | 60.00 (45.00 ; 60.00) | 60.00 (60.00 ; 60.00) |  |  |
|  | Median (min;max) | 60.00 (45.00 ; 70.00) | 60.00 (45.00 ; 65.00) | 60.00 (45.00 ; 70.00) |  |  |
| Left hip internal rotation |  | N=32 | n=19 | n=13 | WMW | 0.798 |
|  | Mean (± SD) | 34.84 (± 11.03) | 34.74 (± 11.96) | 35.00 (± 10.00) |  |  |
|  | Median (Q1;Q3) | 35.00 (30.00 ; 45.00) | 30.00 (25.00 ; 45.00) | 35.00 (30.00 ; 45.00) |  |  |
|  | Median (min;max) | 35.00 (10.00 ; 55.00) | 30.00 (10.00 ; 55.00) | 35.00 (15.00 ; 45.00) |  |  |
| External rotation left hip ° |  | N=32 | n=19 | n=13 | WMW | 0.147 |
|  | Mean (± SD) | 46.41 (± 10.79) | 44.21 (± 10.31) | 49.62 (± 11.08) |  |  |
|  | Median (Q1;Q3) | 45.00 (45.00 ; 50.00) | 45.00 (35.00 ; 50.00) | 45.00 (45.00 ; 60.00) |  |  |
|  | Median (min;max) | 45.00 (25.00 ; 70.00) | 45.00 (30.00 ; 65.00) | 45.00 (25.00 ; 70.00) |  |  |

##### Walking Perimètre

|  | | | **Group Name** | |  | |
| --- | --- | --- | --- | --- | --- | --- |
| **variable** | **Details** | **total population** | **Program MBSR** | **Classic follow-up and general advice** | **test** | **p** |
| Walking perimeter (m) |  | N=5 | n=2 | n=3 | WMW | 0.100 |
|  | Mean (± SD) | 500.00 (± 254.95) | 700.00 (± 141.42) | 366.67 (± 230.94) |  |  |
|  | Median (Q1;Q3) | 500.00 (500.00 ; 600.00) | 700.00 (600.00 ; 800.00) | 500.00 (100.00 ; 500.00) |  |  |
|  | Median (min;max) | 500.00 (100.00 ; 800.00) | 700.00 (600.00 ; 800.00) | 500.00 (100.00 ; 500.00) |  |  |
| No walking limitation (n(%col)) |  | N=27 | n=17 | n=10 | INVALID | . |
|  | Yes | 27 (100.00) | 17 (100.00) | 10 (100.00) |  |  |

#### Painkillers and/or NSAIDs (V1)

|  | | | **Group Name** | |  | |
| --- | --- | --- | --- | --- | --- | --- |
| **variable** | **Details** | **total population** | **Program MBSR** | **Classic follow-up and general advice** | **Test** | **p** |
| bearingANTALGIC_ATC (n(% col)) |  | N=33 | n=17 | n=16 | FISHER | 0.335 |
|  | Tier 1 = PARACETAMOL | 29 (87.88) | 16 (94.12) | 13 (81.25) |  |  |
|  | Tier 2 = Weak Opioids | 4 (12.12) | 1 (5.88) | 3 (18.75) |  |  |
| levelANTALGIC_ATC1 (n(% col)) |  | N=22 | n=10 | n=12 | FISHER | 0.594 |
|  | Tier 1 = PARACETAMOL | 18 (81.82) | 9 (90.00) | 9 (75.00) |  |  |
|  | Tier 2 = Low Opioids | 4 (18.18) | 1 (10.00) | 3 (25.00) |  |  |
| levelANTALGIC_ATC2 (n(% col)) |  | N=17 | n=8 | n=9 | FISHER | 1.000 |
|  | Tier 1 = PARACETAMOL | 16 (94.12) | 8 (100.00) | 8 (88.89) |  |  |
|  | Tier 2 = Opioid Weak | 1 (5.88) | 0 (0.00) | 1 (11.11) |  |  |

|  | | | **Group Name** | |  | |
| --- | --- | --- | --- | --- | --- | --- |
| **variable** | **Details** | **total population** | **Program MBSR** | **Classic follow-up and general advice** | **Test** | **p** |
| analgesic_level at V1 (n(% col)) |  | N=40 | n=20 | n=20 | FISHER | 1.000 |
|  | At least one level 1 | 15 (37.50) | 8 (40.00) | 7 (35.00) |  |  |
|  | At least one level 2 | 5 (12.50) | 2 (10.00) | 3 (15.00) |  |  |
|  | Nothing | 20 (50.00) | 10 (50.00) | 10 (50.00) |  |  |

|  | | | **Group Name** | |  | |
| --- | --- | --- | --- | --- | --- | --- |
| **variable** | **Details** | **total population** | **Program MBSR** | **Classic follow-up and general advice** | **Test** | **p** |
| At least one NSAID (n(% col)) at V1 |  | N=32 | n=18 | n=14 | CHI2 | 0.198 |
|  | No | 20 (62.50) | 13 (72.22) | 7 (50.00) |  |  |
|  | Yes | 12 (37.50) | 5 (27.78) | 7 (50.00) |  |  |

#### Disease activity (V1)

|  | | | **Group Name** | |  | |
| --- | --- | --- | --- | --- | --- | --- |
| **variable** | **Details** | **total population** | **Program MBSR** | **Classic follow-up and general advice** | **Test** | **p** |
| VAS: physician's assessment of illness (n(%col)) |  | N=37 | n=20 | n=17 | FISHER | 0.159 |
|  | Not done | 5 (13.51) | 1 (5.00) | 4 (23.53) |  |  |
|  | done | 32 (86.49) | 19 (95.00) | 13 (76.47) |  |  |
| VAS: physician's assessment of illness |  | N=32 | n=19 | n=13 | WMW | 0.844 |
|  | Mean (± SD) | 48.44 (± 18.34) | 47.89 (± 19.03) | 49.23 (± 18.01) |  |  |
|  | Median (Q1;Q3) | 50.00 (30.00 ; 60.00) | 50.00 (30.00 ; 60.00) | 60.00 (30.00 ; 60.00) |  |  |
|  | Median (min;max) | 50.00 (10.00 ; 70.00) | 50.00 (10.00 ; 70.00) | 60.00 (20.00 ; 70.00) |  |  |

#### Auto-questionnaire (V1)

|  | | | **Group Name** | |  | |
| --- | --- | --- | --- | --- | --- | --- |
| **variable** | **Details** | **total population** | **Program MBSR** | **Classic follow-up and general advice** | **Test** | **p** |
| VAS: Patient pain rating F/NF (n(%col)) |  | N=37 | n=20 | n=17 | FISHER | 0.459 |
|  | Not done | 1 (2.70) | 0 (0.00) | 1 (5.88) |  |  |
|  | done | 36 (97.30) | 20 (100.00) | 16 (94.12) |  |  |
| VAS: Reason for assessment of pain felt by patient NF (n(%col)) |  | N=1 | n=0 | n=1 | INVALID | . |
|  | NO QUESTIONNAIRE SENT | 1 (100.00) | 0 (0.00) | 1 (100.00) |  |  |
| VAS: patient's pain assessment score |  | N=36 | n=20 | n=16 | STUDENT | 0.701 |
|  | Mean (± SD) | 42.89 (± 25.49) | 41.40 (± 25.27) | 44.75 (± 26.47) |  |  |
|  | Median (Q1;Q3) | 42.00 (22.50 ; 66.50) | 42.00 (21.50 ; 57.00) | 43.00 (22.50 ; 71.00) |  |  |
|  | Median (min;max) | 42.00 (0.00 ; 87.00) | 42.00 (0.00 ; 87.00) | 43.00 (1.00 ; 80.00) |  |  |
| VAS: patient's assessment of disease activity F/NF (n(%col)) |  | N=37 | n=20 | n=17 | FISHER | 0.459 |
|  | Not done | 1 (2.70) | 0 (0.00) | 1 (5.88) |  |  |
|  | done | 36 (97.30) | 20 (100.00) | 16 (94.12) |  |  |
| VAS: patient's assessment of disease activity |  | N=36 | n=20 | n=16 | STUDENT | 0.810 |
|  | Mean (± SD) | 47.94 (± 23.32) | 48.80 (± 23.17) | 46.88 (± 24.22) |  |  |
|  | Median (Q1;Q3) | 43.50 (29.50 ; 68.00) | 40.50 (33.50 ; 71.00) | 48.50 (25.00 ; 65.00) |  |  |
|  | Median (min;max) | 43.50 (2.00 ; 90.00) | 40.50 (5.00 ; 84.00) | 48.50 (2.00 ; 90.00) |  |  |
| VAS: patient assessment of health status F/NF (n(%col)) |  | N=37 | n=20 | n=17 | FISHER | 0.459 |
|  | Not done | 1 (2.70) | 0 (0.00) | 1 (5.88) |  |  |
|  | done | 36 (97.30) | 20 (100.00) | 16 (94.12) |  |  |
| VAS: patient's assessment of health status score |  | N=36 | n=20 | n=16 | STUDENT | 0.411 |
|  | Mean (± SD) | 63.53 (± 20.23) | 66.05 (± 16.26) | 60.38 (± 24.52) |  |  |
|  | Median (Q1;Q3) | 68.00 (52.00 ; 76.50) | 68.00 (54.50 ; 77.00) | 68.00 (45.50 ; 75.50) |  |  |
|  | Median (min;max) | 68.00 (3.00 ; 97.00) | 68.00 (27.00 ; 92.00) | 68.00 (3.00 ; 97.00) |  |  |
| WOMAC F/NF (n(%col)) |  | N=37 | n=20 | n=17 | FISHER | 0.459 |
|  | Not done | 1 (2.70) | 0 (0.00) | 1 (5.88) |  |  |
|  | done | 36 (97.30) | 20 (100.00) | 16 (94.12) |  |  |
| SF36 F/NF (n(%col)) |  | N=37 | n=20 | n=17 | FISHER | 0.459 |
|  | Not done | 1 (2.70) | 0 (0.00) | 1 (5.88) |  |  |
|  | done | 36 (97.30) | 20 (100.00) | 16 (94.12) |  |  |
| HAD F/NF (n(%col)) |  | N=37 | n=20 | n=17 | FISHER | 0.204 |
|  | Not done | 2 (5.41) | 0 (0.00) | 2 (11.76) |  |  |
|  | done | 35 (94.59) | 20 (100.00) | 15 (88.24) |  |  |
| FFMQ F/NF (n(%col)) |  | N=37 | n=20 | n=17 | FISHER | 0.459 |
|  | Not done | 1 (2.70) | 0 (0.00) | 1 (5.88) |  |  |
|  | done | 36 (97.30) | 20 (100.00) | 16 (94.12) |  |  |
| Meditation practice Y/N (n(%col)) |  | N=24 | n=20 | n=4 | FISHER | 1.000 |
|  | Not done | 1 (4.17) | 1 (5.00) | 0 (0.00) |  |  |
|  | done | 23 (95.83) | 19 (95.00) | 4 (100.00) |  |  |
| Reason for not practicing meditation daily (n(%col)) |  | N=1 | n=1 | n=0 | INVALID | . |
|  | NOT COME LAST SESSION | 1 (100.00) | 1 (100.00) | 0 (0.00) |  |  |

#### WOMAC (V1)

|  | | | **Group Name** | |  | |
| --- | --- | --- | --- | --- | --- | --- |
| **variable** | **Details** | **total population** | **Program MBSR** | **Classic follow-up and general advice** | **test** | **p** |
| Womac : Score pain |  | N=30 | n=18 | n=12 | STUDENT | 0.191 |
|  | Mean (± SD) | 43.00 (± 21.44) | 47.22 (± 17.59) | 36.67 (± 25.70) |  |  |
|  | Median (Q1;Q3) | 40.00 (30.00 ; 60.00) | 47.50 (30.00 ; 60.00) | 35.00 (20.00 ; 47.50) |  |  |
|  | Median (min;max) | 40.00 (0.00 ; 95.00) | 47.50 (20.00 ; 85.00) | 35.00 (0.00 ; 95.00) |  |  |
| WOMAC : Score function |  | N=26 | n=13 | n=13 | STUDENT | 0.410 |
|  | Mean (± SD) | 39.88 (± 20.86) | 43.33 (± 16.97) | 36.43 (± 24.34) |  |  |
|  | Median (Q1;Q3) | 40.45 (26.47 ; 52.94) | 42.65 (36.76 ; 55.88) | 39.71 (23.53 ; 51.47) |  |  |
|  | Median (min;max) | 40.45 (0.00 ; 76.47) | 42.65 (10.29 ; 70.59) | 39.71 (0.00 ; 76.47) |  |  |
| WOMAC : Score stiffness |  | N=34 | n=19 | n=15 | STUDENT | 0.367 |
|  | Mean (± SD) | 52.21 (± 21.86) | 55.26 (± 23.69) | 48.33 (± 19.40) |  |  |
|  | Median (Q1;Q3) | 50.00 (37.50 ; 75.00) | 62.50 (37.50 ; 75.00) | 50.00 (37.50 ; 62.50) |  |  |
|  | Median (min;max) | 50.00 (12.50 ; 100.00) | 62.50 (12.50 ; 100.00) | 50.00 (12.50 ; 75.00) |  |  |
| WOMAC : Score Total |  | N=23 | n=12 | n=11 | STUDENT | 0.341 |
|  | Mean (± SD) | 42.57 (± 19.59) | 46.38 (± 16.56) | 38.40 (± 22.51) |  |  |
|  | Median (Q1;Q3) | 44.75 (32.84 ; 49.17) | 45.32 (35.13 ; 55.79) | 43.87 (15.78 ; 45.64) |  |  |
|  | Median (min;max) | 44.75 (5.83 ; 81.18) | 45.32 (21.76 ; 81.03) | 43.87 (5.83 ; 81.18) |  |  |

#### SF-36 health status questionnaire without inconsistencies (V1)

|  | | | **Group Name** | |  | |
| --- | --- | --- | --- | --- | --- | --- |
| **variable** | **Details** | **total population** | **Program MBSR** | **Classic follow-up and general advice** | **test** | **p** |
| Score:Physical activity(i) |  | N=36 | n=20 | n=16 | STUDENT | 0.348 |
|  | Mean (± SD) | 56.93 (± 24.34) | 53.48 (± 24.73) | 61.25 (± 23.91) |  |  |
|  | Median (Q1;Q3) | 60.00 (35.00 ; 75.00) | 50.00 (30.00 ; 77.50) | 67.50 (45.00 ; 75.00) |  |  |
|  | Median (min;max) | 60.00 (10.00 ; 95.00) | 50.00 (15.00 ; 90.00) | 67.50 (10.00 ; 95.00) |  |  |
| Score:Limitations due to physical condition(i) |  | N=35 | n=20 | n=15 | WMW | 0.604 |
|  | Mean (± SD) | 47.86 (± 42.60) | 51.25 (± 42.52) | 43.33 (± 43.78) |  |  |
|  | Median (Q1;Q3) | 50.00 (0.00 ; 100.00) | 62.50 (0.00 ; 100.00) | 25.00 (0.00 ; 100.00) |  |  |
|  | Median (min;max) | 50.00 (0.00 ; 100.00) | 62.50 (0.00 ; 100.00) | 25.00 (0.00 ; 100.00) |  |  |
| Score:Limitations due to psychological state(i) |  | N=35 | n=20 | n=15 | WMW | 0.363 |
|  | Mean (± SD) | 60.00 (± 44.87) | 66.67 (± 41.89) | 51.11 (± 48.58) |  |  |
|  | Median (Q1;Q3) | 100.00 (0.00 ; 100.00) | 100.00 (33.33 ; 100.00) | 33.33 (0.00 ; 100.00) |  |  |
|  | Median (min;max) | 100.00 (0.00 ; 100.00) | 100.00 (0.00 ; 100.00) | 33.33 (0.00 ; 100.00) |  |  |
| Score:Life and relationship with others(i) |  | N=36 | n=20 | n=16 | STUDENT | 0.337 |
|  | Mean (± SD) | 66.67 (± 22.95) | 70.00 (± 23.08) | 62.50 (± 22.82) |  |  |
|  | Median (Q1;Q3) | 62.50 (50.00 ; 87.50) | 68.75 (50.00 ; 87.50) | 62.50 (43.75 ; 75.00) |  |  |
|  | Median (min;max) | 62.50 (12.50 ; 100.00) | 68.75 (12.50 ; 100.00) | 62.50 (25.00 ; 100.00) |  |  |
| Score:Physical pain(i) |  | N=36 | n=20 | n=16 | STUDENT | 0.312 |
|  | Mean (± SD) | 45.19 (± 21.89) | 41.85 (± 20.39) | 49.38 (± 23.62) |  |  |
|  | Median (Q1;Q3) | 41.00 (36.00 ; 61.00) | 41.00 (26.50 ; 56.00) | 46.00 (41.00 ; 62.00) |  |  |
|  | Median (min;max) | 41.00 (0.00 ; 84.00) | 41.00 (0.00 ; 84.00) | 46.00 (0.00 ; 84.00) |  |  |
| Score:Mental health(i) |  | N=36 | n=20 | n=16 | STUDENT | 0.031 |
|  | Mean (± SD) | 65.81 (± 15.92) | 70.85 (± 12.56) | 59.50 (± 17.76) |  |  |
|  | Median (Q1;Q3) | 70.00 (52.00 ; 76.00) | 72.00 (64.50 ; 78.00) | 58.00 (46.00 ; 76.00) |  |  |
|  | Median (min;max) | 70.00 (32.00 ; 92.00) | 72.00 (40.00 ; 92.00) | 58.00 (32.00 ; 84.00) |  |  |
| Score:Vitality(i) |  | N=36 | n=20 | n=16 | STUDENT | 0.010 |
|  | Mean (± SD) | 50.23 (± 19.94) | 57.67 (± 18.94) | 40.94 (± 17.53) |  |  |
|  | Median (Q1;Q3) | 50.00 (32.50 ; 65.00) | 57.50 (45.00 ; 71.67) | 40.00 (30.00 ; 57.50) |  |  |
|  | Median (min;max) | 50.00 (10.00 ; 95.00) | 57.50 (25.00 ; 95.00) | 40.00 (10.00 ; 65.00) |  |  |
| Score:Perceived health(i) |  | N=36 | n=20 | n=16 | STUDENT | 0.706 |
|  | Mean (± SD) | 56.53 (± 21.27) | 57.75 (± 17.69) | 55.00 (± 25.59) |  |  |
|  | Median (Q1;Q3) | 57.00 (40.00 ; 74.50) | 57.00 (41.00 ; 69.50) | 57.00 (35.00 ; 77.00) |  |  |
|  | Median (min;max) | 57.00 (5.00 ; 92.00) | 57.00 (35.00 ; 92.00) | 57.00 (5.00 ; 92.00) |  |  |
| Aggregate physical score(i) |  | N=35 | n=20 | n=15 | STUDENT | 0.334 |
|  | Mean (± SD) | 37.80 (± 8.61) | 36.56 (± 7.94) | 39.45 (± 9.46) |  |  |
|  | Median (Q1;Q3) | 38.16 (30.64 ; 43.94) | 36.04 (30.22 ; 41.88) | 42.48 (30.64 ; 46.97) |  |  |
|  | Median (min;max) | 38.16 (22.16 ; 55.69) | 36.04 (22.16 ; 53.08) | 42.48 (24.75 ; 55.69) |  |  |
| Aggregate psychic score(i) |  | N=35 | n=20 | n=15 | WMW | 0.013 |
|  | Mean (± SD) | 46.90 (± 10.39) | 50.70 (± 7.88) | 41.84 (± 11.39) |  |  |
|  | Median (Q1;Q3) | 51.74 (38.16 ; 55.41) | 54.72 (45.14 ; 56.48) | 40.58 (30.49 ; 52.35) |  |  |
|  | Median (min;max) | 51.74 (29.22 ; 61.50) | 54.72 (29.90 ; 59.45) | 40.58 (29.22 ; 61.50) |  |  |
| Sum of the scores of the 8 dim of the SF36(/800)(i) |  | N=35 | n=20 | n=15 | STUDENT | 0.327 |
|  | Mean (± SD) | 445.60 (± 164.03) | 469.51 (± 148.69) | 413.71 (± 182.85) |  |  |
|  | Median (Q1;Q3) | 454.50 (309.00 ; 595.00) | 475.67 (367.33 ; 601.50) | 405.33 (257.50 ; 577.00) |  |  |
|  | Median (min;max) | 454.50 (150.50 ; 738.00) | 475.67 (187.50 ; 738.00) | 405.33 (150.50 ; 707.00) |  |  |
| Average of the scores of the 8 dim of the SF36 (i) |  | N=35 | n=20 | n=15 | STUDENT | 0.327 |
|  | Mean (± SD) | 55.70 (± 20.50) | 58.69 (± 18.59) | 51.71 (± 22.86) |  |  |
|  | Median (Q1;Q3) | 56.81 (38.63 ; 74.38) | 59.46 (45.92 ; 75.19) | 50.67 (32.19 ; 72.13) |  |  |
|  | Median (min;max) | 56.81 (18.81 ; 92.25) | 59.46 (23.44 ; 92.25) | 50.67 (18.81 ; 88.38) |  |  |

#### HADS (V1)

|  | | | **Group Name** | |  | |
| --- | --- | --- | --- | --- | --- | --- |
| **variable** | **Details** | **total population** | **Program MBSR** | **Classic follow-up and general advice** | **test** | **p** |
| HADS : Score Anxiety |  | N=35 | n=20 | n=15 | STUDENT | 0.088 |
|  | Mean (± SD) | 6.17 (± 4.09) | 5.15 (± 3.38) | 7.53 (± 4.66) |  |  |
|  | Median (Q1;Q3) | 6.00 (3.00 ; 8.00) | 4.50 (2.50 ; 8.00) | 7.00 (4.00 ; 12.00) |  |  |
|  | Median (min;max) | 6.00 (0.00 ; 16.00) | 4.50 (0.00 ; 13.00) | 7.00 (1.00 ; 16.00) |  |  |
| HADS : Score Depression |  | N=35 | n=20 | n=15 | STUDENT | 0.142 |
|  | Mean (± SD) | 3.97 (± 2.87) | 3.35 (± 2.18) | 4.80 (± 3.51) |  |  |
|  | Median (Q1;Q3) | 4.00 (2.00 ; 5.00) | 3.00 (1.50 ; 5.00) | 5.00 (3.00 ; 7.00) |  |  |
|  | Median (min;max) | 4.00 (0.00 ; 14.00) | 3.00 (0.00 ; 7.00) | 5.00 (0.00 ; 14.00) |  |  |
| HADS : Score Total |  | N=35 | n=20 | n=15 | STUDENT | 0.079 |
|  | Mean (± SD) | 10.14 (± 6.40) | 8.50 (± 4.94) | 12.33 (± 7.58) |  |  |
|  | Median (Q1;Q3) | 9.00 (5.00 ; 13.00) | 9.00 (4.00 ; 12.00) | 11.00 (7.00 ; 18.00) |  |  |
|  | Median (min;max) | 9.00 (1.00 ; 28.00) | 9.00 (1.00 ; 20.00) | 11.00 (2.00 ; 28.00) |  |  |

#### FFMQ (V1)

|  | | | **Group Name** | |  | |
| --- | --- | --- | --- | --- | --- | --- |
| **variable** | **Details** | **total population** | **Program MBSR** | **Classic follow-up and general advice** | **test** | **p** |
| FFMQ : Observation Factor |  | N=36 | n=20 | n=16 | STUDENT | 0.003 |
|  | Mean (± SD) | 29.50 (± 5.57) | 31.85 (± 4.23) | 26.56 (± 5.76) |  |  |
|  | Median (Q1;Q3) | 30.00 (25.50 ; 33.00) | 32.00 (28.00 ; 34.50) | 26.00 (22.00 ; 30.50) |  |  |
|  | Median (min;max) | 30.00 (16.00 ; 39.00) | 32.00 (25.00 ; 39.00) | 26.00 (16.00 ; 37.00) |  |  |
| FFMQ: Description Factor Experience |  | N=35 | n=20 | n=15 | STUDENT | 0.878 |
|  | Mean (± SD) | 29.91 (± 5.90) | 30.05 (± 4.70) | 29.73 (± 7.38) |  |  |
|  | Median (Q1;Q3) | 31.00 (27.00 ; 33.00) | 31.00 (28.00 ; 33.00) | 31.00 (24.00 ; 36.00) |  |  |
|  | Median (min;max) | 31.00 (18.00 ; 40.00) | 31.00 (20.00 ; 37.00) | 31.00 (18.00 ; 40.00) |  |  |
| FFMQ : Factor Full consciousness |  | N=35 | n=19 | n=16 | STUDENT | 0.717 |
|  | Mean (± SD) | 28.57 (± 6.55) | 28.95 (± 6.45) | 28.13 (± 6.85) |  |  |
|  | Median (Q1;Q3) | 30.00 (24.00 ; 33.00) | 31.00 (24.00 ; 33.00) | 28.50 (24.50 ; 33.00) |  |  |
|  | Median (min;max) | 30.00 (15.00 ; 40.00) | 31.00 (18.00 ; 40.00) | 28.50 (15.00 ; 39.00) |  |  |
| FFMQ: Private Experience Factor |  | N=34 | n=19 | n=15 | STUDENT | 0.023 |
|  | Mean (± SD) | 21.94 (± 5.42) | 23.79 (± 5.69) | 19.60 (± 4.12) |  |  |
|  | Median (Q1;Q3) | 22.00 (18.00 ; 27.00) | 26.00 (19.00 ; 28.00) | 20.00 (17.00 ; 22.00) |  |  |
|  | Median (min;max) | 22.00 (10.00 ; 31.00) | 26.00 (10.00 ; 31.00) | 20.00 (11.00 ; 27.00) |  |  |
| FFMQ: Non-judgment Factor |  | N=35 | n=20 | n=15 | STUDENT | 0.861 |
|  | Mean (± SD) | 28.31 (± 6.27) | 28.15 (± 6.20) | 28.53 (± 6.58) |  |  |
|  | Median (Q1;Q3) | 28.00 (23.00 ; 32.00) | 28.50 (23.00 ; 31.50) | 28.00 (25.00 ; 33.00) |  |  |
|  | Median (min;max) | 28.00 (17.00 ; 40.00) | 28.50 (17.00 ; 40.00) | 28.00 (17.00 ; 39.00) |  |  |
| FFMQ : Mindfulness |  | N=31 | n=18 | n=13 | STUDENT | 0.106 |
|  | Mean (± SD) | 139.35 (± 22.85) | 145.00 (± 19.99) | 131.54 (± 24.99) |  |  |
|  | Median (Q1;Q3) | 141.00 (125.00 ; 155.00) | 150.00 (135.00 ; 155.00) | 132.00 (114.00 ; 151.00) |  |  |
|  | Median (min;max) | 141.00 (84.00 ; 181.00) | 150.00 (104.00 ; 181.00) | 132.00 (84.00 ; 173.00) |  |  |

#### Self-questionnaire "Daily practice between the 8 sessions

|  | | | **Group Name** | |  | |
| --- | --- | --- | --- | --- | --- | --- |
| **variable** | **Details** | **total population** | **Program MBSR** | **Classic follow-up and general advice** | **Test** | **p** |
| Meditation sessions Q 1 (n(%col)) |  | N=3 | n=1 | n=2 | INVALID | . |
|  | Yes | 3 (100.00) | 1 (100.00) | 2 (100.00) |  |  |
| Meditation sessions Q 2 (n(%col)) |  | N=2 | n=2 | n=0 | INVALID | . |
|  | Yes | 2 (100.00) | 2 (100.00) | 0 (0.00) |  |  |
| Meditation sessions Q 3 (n(%col)) |  | N=6 | n=6 | n=0 | INVALID | . |
|  | Yes | 6 (100.00) | 6 (100.00) | 0 (0.00) |  |  |
| Meditation sessions Q 4 (n(%col)) |  | N=5 | n=5 | n=0 | INVALID | . |
|  | Yes | 5 (100.00) | 5 (100.00) | 0 (0.00) |  |  |
| Meditation sessions Q 5 (n(%col)) |  | N=5 | n=5 | n=0 | INVALID | . |
|  | Yes | 5 (100.00) | 5 (100.00) | 0 (0.00) |  |  |
| Reason for meditation NF (n(%col)) |  | N=11 | n=9 | n=2 | - | - |
|  | - 1 DAY TRAVEL - 1 DAY GASTROENTERITIS | 1 (9.09) | 1 (11.11) | 0 (0.00) |  |  |
|  | IRREGULARLY BECAUSE I HAD DIFFICULTY IN KEEPING UP WITH THE DAILY ROUTINE - CHAN | 1 (9.09) | 1 (11.11) | 0 (0.00) |  |  |
|  | I WAS DESTABILIZED IN THE LAST WEEK BY THE PRESENCE OF MY SON AND THE FACT THAT I HAD NO TIME TO PRACTICE. | 1 (9.09) | 1 (11.11) | 0 (0.00) |  |  |
|  | TIME TO PRACTICE. I WAS CAUGHT UP IN A JOB OF RESPONSIBILITY THAT TOOK A LOT OUT OF ME. | 1 (9.09) | 1 (11.11) | 0 (0.00) |  |  |
|  | THE PAIN IN MY SHOULDER AND KNEE BECAME TOO MUCH. THE GRI | 1 (9.09) | 1 (11.11) | 0 (0.00) |  |  |
|  | NO TIME | 1 (9.09) | 1 (11.11) | 0 (0.00) |  |  |
|  | PATIENT CLASSIC ARM | 1 (9.09) | 0 (0.00) | 1 (50.00) |  |  |
|  | PATIENT CLASSIC FOLLOW-UP | 1 (9.09) | 0 (0.00) | 1 (50.00) |  |  |
|  | WHEN PRACTICING SOPHROLOGY, HAS DIFFICULTY IN DOING ANOTHER MEDIATION PRACTICE | 1 (9.09) | 1 (11.11) | 0 (0.00) |  |  |
|  | A LITTLE BIT OF RELAXATION SOME WEEKENDS | 1 (9.09) | 1 (11.11) | 0 (0.00) |  |  |
|  | TRAVEL | 1 (9.09) | 1 (11.11) | 0 (0.00) |  |  |
| Attendance Y/N (n(%col)) |  | N=22 | n=20 | n=2 | FISHER | 0.494 |
|  | No | 13 (59.09) | 11 (55.00) | 2 (100.00) |  |  |
|  | Yes | 9 (40.91) | 9 (45.00) | 0 (0.00) |  |  |
| Missed sessions (n(%col)) |  | N=12 | n=10 | n=2 | - | - |
|  | 7 | 1 (8.33) | 1 (10.00) | 0 (0.00) |  |  |
|  | 7TH SESSION | 1 (8.33) | 1 (10.00) | 0 (0.00) |  |  |
|  | THE FOURTH SESSION | 1 (8.33) | 1 (10.00) | 0 (0.00) |  |  |
|  | THE SEVENTH | 1 (8.33) | 1 (10.00) | 0 (0.00) |  |  |
|  | THE LAST 4 SESSIONS | 1 (8.33) | 1 (10.00) | 0 (0.00) |  |  |
|  | CLASSIC ARM PATIENT | 1 (8.33) | 0 (0.00) | 1 (50.00) |  |  |
|  | CLASSIC FOLLOW-UP PATIENT | 1 (8.33) | 0 (0.00) | 1 (50.00) |  |  |
|  | SESSION 8 | 1 (8.33) | 1 (10.00) | 0 (0.00) |  |  |
|  | SESSION OF 13/11 | 1 (8.33) | 1 (10.00) | 0 (0.00) |  |  |
|  | SESSION OF 23/10 | 1 (8.33) | 1 (10.00) | 0 (0.00) |  |  |
|  | SESSION NUM 3 | 1 (8.33) | 1 (10.00) | 0 (0.00) |  |  |
|  | ONLY THE FIRST SESSION HAS BEEN DONE | 1 (8.33) | 1 (10.00) | 0 (0.00) |  |  |
| Missed sessions for reason (n(%col)) |  | N=13 | n=11 | n=2 | - | - |
|  | FLIGHT SCHEDULES | 1 (7.69) | 1 (9.09) | 0 (0.00) |  |  |
|  | ILLNESS | 1 (7.69) | 1 (9.09) | 0 (0.00) |  |  |
|  | MEDICAL | 1 (7.69) | 1 (9.09) | 0 (0.00) |  |  |
|  | NO TIME | 1 (7.69) | 1 (9.09) | 0 (0.00) |  |  |
|  | CLASSIC ARM PATIENT | 1 (7.69) | 0 (0.00) | 1 (50.00) |  |  |
|  | CLASSIC FOLLOW-UP PATIENT | 1 (7.69) | 0 (0.00) | 1 (50.00) |  |  |
|  | PERSONAL | 1 (7.69) | 1 (9.09) | 0 (0.00) |  |  |
|  | PERSONAL | 2 (15.38) | 2 (18.18) | 0 (0.00) |  |  |
|  | PERSONNEL (STAY OUTSIDE OF MONTPELLIER SCHEDULED IN ADVANCE) | 1 (7.69) | 1 (9.09) | 0 (0.00) |  |  |
|  | PERSONAL: ASSOCIATION MEETING | 1 (7.69) | 1 (9.09) | 0 (0.00) |  |  |
|  | WHEN I WAS AWAY FROM HOME TO TAKE CARE OF MY OLD PARENTS, I HAD TO GO TO THE HOSPITAL. | 1 (7.69) | 1 (9.09) | 0 (0.00) |  |  |
|  | SESSION 1: WAS LOOKING AFTER HER GRANDDAUGHTER AND SESSION 8: REASON NOT KNOWN | 1 (7.69) | 1 (9.09) | 0 (0.00) |  |  |

|  | | | **Group Name** | |  | |
| --- | --- | --- | --- | --- | --- | --- |
| **variable** | **Details** | **total population** | **Program MBSR** | **Classic follow-up and general advice** | **Test** | **p** |
| V1_Recommended daily meditation practice (n(% col)) |  | N=21 | n=19 | n=2 | FISHER | 0.019 |
|  | 0 to 1 time / week | 3 (14.29) | 1 (5.26) | 2 (100.00) |  |  |
|  | 2 to 3 times / week | 2 (9.52) | 2 (10.53) | 0 (0.00) |  |  |
|  | 4 to 5 times / week | 6 (28.57) | 6 (31.58) | 0 (0.00) |  |  |
|  | Every day of the week, but incomplete in terms of what was said | 5 (23.81) | 5 (26.32) | 0 (0.00) |  |  |
|  | Every day of the week is fairly complete | 5 (23.81) | 5 (26.32) | 0 (0.00) |  |  |

### Visit V2- 3 months post-program (+/- 15days)

|  | | | **Group Name** | |  | |
| --- | --- | --- | --- | --- | --- | --- |
| **variable** | **Details** | **total population** | **Program MBSR** | **Classic follow-up and general advice** | **Test** | **p** |
| Follow-up done Y/N (n(%col)) |  | N=39 | n=20 | n=19 | FISHER | 0.020 |
|  | No | 5 (12.82) | 0 (0.00) | 5 (26.32) |  |  |
|  | Yes | 34 (87.18) | 20 (100.00) | 14 (73.68) |  |  |
| Visit not made: Questionnaires sent Y/N (n(%col)) |  | N=2 | n=0 | n=2 | INVALID | . |
|  | Yes | 2 (100.00) | 0 (0.00) | 2 (100.00) |  |  |
| Reason Not Done (n(%col)) |  | N=5 | n=0 | n=5 | INVALID | . |
|  | Other reason | 5 (100.00) | 0 (0.00) | 5 (100.00) |  |  |
| Other Reason Not Done (n(%col)) |  | N=5 | n=0 | n=5 | INVALID | . |
|  | NO VISIT | 2 (40.00) | 0 (0.00) | 2 (40.00) |  |  |
|  | PATIENT DID NOT COME | 1 (20.00) | 0 (0.00) | 1 (20.00) |  |  |
|  | LOST TO VIEW | 2 (40.00) | 0 (0.00) | 2 (40.00) |  |  |
| AR Alert (n(%col)) |  | N=5 | n=2 | n=3 | INVALID | . |
|  | Remember to enter the EvI/EvIG at the end of the booklet!!! | 5 (100.00) | 2 (100.00) | 3 (100.00) |  |  |
| Undesirable Ev Y/N (n(%col)) |  | N=37 | n=20 | n=17 | FISHER | 0.644 |
|  | No | 32 (86.49) | 18 (90.00) | 14 (82.35) |  |  |
|  | Yes | 5 (13.51) | 2 (10.00) | 3 (17.65) |  |  |

#### Clinical Exam (V2)

|  | | | **Group Name** | |  | |
| --- | --- | --- | --- | --- | --- | --- |
| **variable** | **Details** | **total population** | **Program MBSR** | **Classic follow-up and general advice** | **test** | **p** |
| Weight |  | N=34 | n=20 | n=14 | WMW | 0.22 |
|  | Mean (± SD) | 74.93 (± 18.60) | 72.73 (± 20.21) | 78.07 (± 16.22) |  |  |
|  | Median (Q1;Q3) | 71.00 (62.00 ; 87.00) | 68.00 (55.00 ; 88.50) | 76.00 (70.00 ; 80.00) |  |  |
|  | Median (min;max) | 71.00 (48.00 ; 123.50) | 68.00 (48.00 ; 122.00) | 76.00 (56.00 ; 123.50) |  |  |
| Height |  | N=34 | n=20 | n=14 | WMW | 0.82 |
|  | Mean (± SD) | 164.65 (± 8.71) | 164.85 (± 10.16) | 164.36 (± 6.44) |  |  |
|  | Median (Q1;Q3) | 162.50 (159.00 ; 170.00) | 162.00 (158.00 ; 169.00) | 164.50 (159.00 ; 170.00) |  |  |
|  | Median (min;max) | 162.50 (152.00 ; 191.00) | 162.00 (152.00 ; 191.00) | 164.50 (156.00 ; 175.00) |  |  |
| BMI |  | N=34 | n=20 | n=14 | WMW | 0.29 |
|  | Mean (± SD) | 27.64 (± 6.64) | 26.61 (± 6.33) | 29.11 (± 7.03) |  |  |
|  | Median (Q1;Q3) | 27.52 (22.23 ; 30.78) | 25.65 (21.22 ; 30.19) | 28.27 (25.06 ; 31.64) |  |  |
|  | Median (min;max) | 27.52 (18.42 ; 48.85) | 25.65 (18.42 ; 41.50) | 28.27 (19.38 ; 48.85) |  |  |
| BP Systole |  | N=33 | n=19 | n=14 | STUDENT | 0.12 |
|  | Mean (± SD) | 129.64 (± 13.90) | 132.89 (± 14.09) | 125.21 (± 12.81) |  |  |
|  | Median (Q1;Q3) | 130.00 (118.00 ; 140.00) | 132.00 (126.00 ; 143.00) | 124.50 (113.00 ; 135.00) |  |  |
|  | Median (min;max) | 130.00 (109.00 ; 166.00) | 132.00 (109.00 ; 166.00) | 124.50 (111.00 ; 146.00) |  |  |

##### Clinical examination of joints (V2)

|  | | | **Group Name** | |  | |
| --- | --- | --- | --- | --- | --- | --- |
| **variable** | **Details** | **total population** | **Program MBSR** | **Classic follow-up and general advice** | **Test** | **p** |
| Right knee pain Y/N (n(%col)) |  | N=34 | n=20 | n=14 | CHI2 | 0.324 |
|  | No | 18 (52.94) | 12 (60.00) | 6 (42.86) |  |  |
|  | Yes | 16 (47.06) | 8 (40.00) | 8 (57.14) |  |  |
| Right knee effusion Y/N (n(%col)) |  | N=34 | n=20 | n=14 | FISHER | 0.126 |
|  | No | 30 (88.24) | 16 (80.00) | 14 (100.00) |  |  |
|  | Yes | 4 (11.76) | 4 (20.00) | 0 (0.00) |  |  |
| Patellofemoral syndrome right knee Y/N (n(%col)) |  | N=34 | n=20 | n=14 | CHI2 | 0.643 |
|  | No | 21 (61.76) | 13 (65.00) | 8 (57.14) |  |  |
|  | Yes | 13 (38.24) | 7 (35.00) | 6 (42.86) |  |  |
| Flessum right knee Y/N (n(%col)) |  | N=34 | n=20 | n=14 | INVALID | . |
|  | No | 34 (100.00) | 20 (100.00) | 14 (100.00) |  |  |
| Left knee pain Y/N (n(%col)) |  | N=34 | n=20 | n=14 | CHI2 | 0.868 |
|  | No | 14 (41.18) | 8 (40.00) | 6 (42.86) |  |  |
|  | Yes | 20 (58.82) | 12 (60.00) | 8 (57.14) |  |  |
| Left knee effusion Y/N (n(%col)) |  | N=34 | n=20 | n=14 | FISHER | 0.555 |
|  | No | 31 (91.18) | 19 (95.00) | 12 (85.71) |  |  |
|  | Yes | 3 (8.82) | 1 (5.00) | 2 (14.29) |  |  |
| Patellofemoral syndrome left knee Y/N (n(%col)) |  | N=34 | n=20 | n=14 | FISHER | 1.000 |
|  | No | 23 (67.65) | 13 (65.00) | 10 (71.43) |  |  |
|  | Yes | 11 (32.35) | 7 (35.00) | 4 (28.57) |  |  |
| Flessum left knee Y/N (n(%col)) |  | N=34 | n=20 | n=14 | FISHER | 1.000 |
|  | No | 33 (97.06) | 19 (95.00) | 14 (100.00) |  |  |
|  | Yes | 1 (2.94) | 1 (5.00) | 0 (0.00) |  |  |
| Right hip pain Y/N (n(%col)) |  | N=34 | n=20 | n=14 | FISHER | 1.000 |
|  | No | 23 (67.65) | 14 (70.00) | 9 (64.29) |  |  |
|  | Yes | 4 (11.76) | 2 (10.00) | 2 (14.29) |  |  |
|  | NA | 7 (20.59) | 4 (20.00) | 3 (21.43) |  |  |
| Right hip Y/N (n(%col)) |  | N=34 | n=20 | n=14 | FISHER | 0.672 |
|  | No | 28 (82.35) | 17 (85.00) | 11 (78.57) |  |  |
|  | NA | 6 (17.65) | 3 (15.00) | 3 (21.43) |  |  |
| Left hip pain Y/N (n(%col)) |  | N=34 | n=20 | n=14 | FISHER | 0.672 |
|  | No | 24 (70.59) | 15 (75.00) | 9 (64.29) |  |  |
|  | Yes | 1 (2.94) | 0 (0.00) | 1 (7.14) |  |  |
|  | NA | 9 (26.47) | 5 (25.00) | 4 (28.57) |  |  |
| Flessum left hip Y/N (n(%col)) |  | N=34 | n=20 | n=14 | FISHER | 0.672 |
|  | No | 28 (82.35) | 17 (85.00) | 11 (78.57) |  |  |
|  | NA | 6 (17.65) | 3 (15.00) | 3 (21.43) |  |  |

|  | | | **Group Name** | |  | |
| --- | --- | --- | --- | --- | --- | --- |
| **variable** | **Details** | **total population** | **Program MBSR** | **Classic follow-up and general advice** | **Test** | **p** |
| Bilateral knee pain (n(% col)) |  | N=34 | n=20 | n=14 | CHI2 | 0.643 |
|  | No | 21 (61.76) | 13 (65.00) | 8 (57.14) |  |  |
|  | Yes | 13 (38.24) | 7 (35.00) | 6 (42.86) |  |  |
| Unilateral knee pain (n(% col)) |  | N=34 | n=20 | n=14 | FISHER | 1.000 |
|  | No | 24 (70.59) | 14 (70.00) | 10 (71.43) |  |  |
|  | Yes | 10 (29.41) | 6 (30.00) | 4 (28.57) |  |  |
| Bilateral knee effusion (n(% col)) |  | N=34 | n=20 | n=14 | FISHER | 1.000 |
|  | No | 33 (97.06) | 19 (95.00) | 14 (100.00) |  |  |
|  | Yes | 1 (2.94) | 1 (5.00) | 0 (0.00) |  |  |
| Unilateral knee effusion (n(% col)) |  | N=34 | n=20 | n=14 | FISHER | 1.000 |
|  | No | 29 (85.29) | 17 (85.00) | 12 (85.71) |  |  |
|  | Yes | 5 (14.71) | 3 (15.00) | 2 (14.29) |  |  |
| At least one knee effusion (n(% col)) |  | N=34 | n=20 | n=14 | FISHER | 1.000 |
|  | No | 28 (82.35) | 16 (80.00) | 12 (85.71) |  |  |
|  | Yes | 6 (17.65) | 4 (20.00) | 2 (14.29) |  |  |
| Bilateral femoral patellar knee syndrome (n(% col)) |  | N=34 | n=20 | n=14 | FISHER | 1.000 |
|  | No | 26 (76.47) | 15 (75.00) | 11 (78.57) |  |  |
|  | Yes | 8 (23.53) | 5 (25.00) | 3 (21.43) |  |  |
| Unilateral femoral patellar knee syndrome (n(% col)) |  | N=34 | n=20 | n=14 | FISHER | 0.689 |
|  | No | 26 (76.47) | 16 (80.00) | 10 (71.43) |  |  |
|  | Yes | 8 (23.53) | 4 (20.00) | 4 (28.57) |  |  |
| At least one femoro patellar knee syndrome (n(% col)) |  | N=34 | n=20 | n=14 | CHI2 | 0.774 |
|  | No | 18 (52.94) | 11 (55.00) | 7 (50.00) |  |  |
|  | Yes | 16 (47.06) | 9 (45.00) | 7 (50.00) |  |  |
| Flessum knee bilateral (n(% col)) |  | N=34 | n=20 | n=14 | INVALID | . |
|  | No | 34 (100.00) | 20 (100.00) | 14 (100.00) |  |  |
| Unilateral knee flessum (n(% col)) |  | N=34 | n=20 | n=14 | FISHER | 1.000 |
|  | No | 33 (97.06) | 19 (95.00) | 14 (100.00) |  |  |
|  | Yes | 1 (2.94) | 1 (5.00) | 0 (0.00) |  |  |
| At least one knee flessum (n(% col)) |  | N=34 | n=20 | n=14 | FISHER | 1.000 |
|  | No | 33 (97.06) | 19 (95.00) | 14 (100.00) |  |  |
|  | Yes | 1 (2.94) | 1 (5.00) | 0 (0.00) |  |  |
| Bilateral hip pain (n(% col)) |  | N=25 | n=15 | n=10 | FISHER | 0.400 |
|  | No | 24 (96.00) | 15 (100.00) | 9 (90.00) |  |  |
|  | Yes | 1 (4.00) | 0 (0.00) | 1 (10.00) |  |  |
| Unilateral hip pain (n(% col)) |  | N=27 | n=16 | n=11 | FISHER | 0.499 |
|  | No | 25 (92.59) | 14 (87.50) | 11 (100.00) |  |  |
|  | Yes | 2 (7.41) | 2 (12.50) | 0 (0.00) |  |  |
| Flessum de hanche bilatéral (n(% col)) |  | N=28 | n=17 | n=11 | INVALID | . |
|  | No | 28 (100.00) | 17 (100.00) | 11 (100.00) |  |  |
| Flessum de hanche unilatéral (n(% col)) |  | N=28 | n=17 | n=11 | INVALID | . |
|  | No | 28 (100.00) | 17 (100.00) | 11 (100.00) |  |  |
| Au moins un flessum hanche (n(% col)) |  | N=28 | n=17 | n=11 | INVALID | . |
|  | No | 28 (100.00) | 17 (100.00) | 11 (100.00) |  |  |

|  | | | **Group Name** | |  | |
| --- | --- | --- | --- | --- | --- | --- |
| **variable** | **modalités** | **population totale** | **Program MBSR** | **Classic follow-up and general advice** | **Test** | **p** |
| At least one painful joint at V2 (n(%col)) |  | N=31 | n=18 | n=13 | FISHER | 1.000 |
|  | No | 6 (19.35) | 4 (22.22) | 2 (15.38) |  |  |
|  | Yes | 25 (80.65) | 14 (77.78) | 11 (84.62) |  |  |

##### Passive hip mobility (V2)

|  | | | **Group Name** | |  | |
| --- | --- | --- | --- | --- | --- | --- |
| **variable** | **Details** | **total population** | **Program MBSR** | **Classic follow-up and general advice** | **test** | **p** |
| Right hip flexion |  | N=34 | n=20 | n=14 | WMW | 0.749 |
|  | Mean (± SD) | 129.12 (± 20.28) | 131.25 (± 16.85) | 126.07 (± 24.74) |  |  |
|  | Median (Q1;Q3) | 132.50 (130.00 ; 140.00) | 135.00 (125.00 ; 140.00) | 130.00 (130.00 ; 140.00) |  |  |
|  | Median (min;max) | 132.50 (55.00 ; 160.00) | 135.00 (90.00 ; 160.00) | 130.00 (55.00 ; 145.00) |  |  |
| Right hip abduction |  | N=34 | n=20 | n=14 | WMW | 1.000 |
|  | Mean (± SD) | 59.12 (± 12.34) | 60.00 (± 13.08) | 57.86 (± 11.55) |  |  |
|  | Median (Q1;Q3) | 60.00 (60.00 ; 60.00) | 60.00 (52.50 ; 60.00) | 60.00 (60.00 ; 60.00) |  |  |
|  | Median (min;max) | 60.00 (30.00 ; 100.00) | 60.00 (45.00 ; 100.00) | 60.00 (30.00 ; 80.00) |  |  |
| Internal rotation right hip ° |  | N=34 | n=20 | n=14 | WMW | 0.389 |
|  | Mean (± SD) | 31.56 (± 11.28) | 30.15 (± 11.98) | 33.57 (± 10.27) |  |  |
|  | Median (Q1;Q3) | 30.00 (30.00 ; 45.00) | 30.00 (20.00 ; 40.00) | 30.00 (30.00 ; 45.00) |  |  |
|  | Median (min;max) | 30.00 (3.00 ; 45.00) | 30.00 (3.00 ; 45.00) | 30.00 (10.00 ; 45.00) |  |  |
|  |  | N=34 | n=20 | n=14 | WMW | 0.355 |
|  | Mean (± SD) | 47.79 (± 12.92) | 46.25 (± 13.36) | 50.00 (± 12.40) |  |  |
|  | Median (Q1;Q3) | 45.00 (45.00 ; 50.00) | 45.00 (45.00 ; 47.50) | 45.00 (45.00 ; 50.00) |  |  |
|  | Median (min;max) | 45.00 (10.00 ; 80.00) | 45.00 (10.00 ; 80.00) | 45.00 (30.00 ; 80.00) |  |  |
| Left hip flexion |  | N=34 | n=20 | n=14 | WMW | 0.957 |
|  | Mean (± SD) | 129.41 (± 20.18) | 131.00 (± 15.86) | 127.14 (± 25.62) |  |  |
|  | Median (Q1;Q3) | 130.00 (130.00 ; 140.00) | 130.00 (125.00 ; 140.00) | 130.00 (130.00 ; 140.00) |  |  |
|  | Median (min;max) | 130.00 (55.00 ; 160.00) | 130.00 (90.00 ; 160.00) | 130.00 (55.00 ; 150.00) |  |  |
| Left hip abduction |  | N=34 | n=20 | n=14 | WMW | 0.954 |
|  | Mean (± SD) | 57.79 (± 10.74) | 58.75 (± 11.57) | 56.43 (± 9.69) |  |  |
|  | Median (Q1;Q3) | 60.00 (45.00 ; 60.00) | 60.00 (45.00 ; 60.00) | 60.00 (60.00 ; 60.00) |  |  |
|  | Median (min;max) | 60.00 (30.00 ; 80.00) | 60.00 (45.00 ; 80.00) | 60.00 (30.00 ; 65.00) |  |  |
| Internal rotation left hip ° |  | N=34 | n=20 | n=14 | WMW | 0.297 |
|  | Mean (± SD) | 33.09 (± 8.71) | 31.75 (± 8.16) | 35.00 (± 9.41) |  |  |
|  | Median (Q1;Q3) | 30.00 (30.00 ; 40.00) | 30.00 (30.00 ; 37.50) | 32.50 (30.00 ; 45.00) |  |  |
|  | Median (min;max) | 30.00 (15.00 ; 45.00) | 30.00 (20.00 ; 45.00) | 32.50 (15.00 ; 45.00) |  |  |
| External rotation left hip ° |  | N=34 | n=20 | n=14 | WMW | 0.411 |
|  | Mean (± SD) | 52.21 (± 12.98) | 51.25 (± 13.75) | 53.57 (± 12.16) |  |  |
|  | Median (Q1;Q3) | 45.00 (45.00 ; 60.00) | 45.00 (45.00 ; 60.00) | 50.00 (45.00 ; 60.00) |  |  |
|  | Median (min;max) | 45.00 (35.00 ; 90.00) | 45.00 (35.00 ; 90.00) | 50.00 (35.00 ; 80.00) |  |  |

##### Walking Perimeter (V2)

|  | | | **Nom groupe rando** | |  | |
| --- | --- | --- | --- | --- | --- | --- |
| **variable** | **Details** | **total population** | **Programme MBSR** | **Classic follow-up and general advice** | **test** | **p** |
| Walking perimeter (m) |  | N=5 | n=3 | n=2 | WMW | 0.700 |
|  | Mean (± SD) | 420.00 (± 164.32) | 366.67 (± 208.17) | 500.00 (± 0.00) |  |  |
|  | Median (Q1;Q3) | 500.00 (300.00 ; 500.00) | 300.00 (200.00 ; 600.00) | 500.00 (500.00 ; 500.00) |  |  |
|  | Median (min;max) | 500.00 (200.00 ; 600.00) | 300.00 (200.00 ; 600.00) | 500.00 (500.00 ; 500.00) |  |  |
| No walking limitation (n(%col)) |  | N=29 | n=17 | n=12 | INVALID | . |
|  | Yes | 29 (100.00) | 17 (100.00) | 12 (100.00) |  |  |

#### Painkillers and/or NSAIDs (V2)

|  | | | **Group Name** | |  | |
| --- | --- | --- | --- | --- | --- | --- |
| **variable** | **Details** | **total population** | **Program MBSR** | **Classic follow-up and general advice** | **Test** | **p** |
| Analgesic level atV2 (n(% col)) |  | N=40 | n=20 | n=20 | FISHER | 0.480 |
|  | At least one level 1 | 15 (37.50) | 9 (45.00) | 6 (30.00) |  |  |
|  | At least one level 2 | 3 (7.50) | 2 (10.00) | 1 (5.00) |  |  |
|  | Nothing | 22 (55.00) | 9 (45.00) | 13 (65.00) |  |  |

|  | | | **Group Name** | |  | |
| --- | --- | --- | --- | --- | --- | --- |
| **variable** | **Details** | **total population** | **Program MBSR** | **Classic follow-up and general advice** | **Test** | **p** |
| At least one NSAID (n(% col)) at V2 |  | N=35 | n=20 | n=15 | FISHER | 0.712 |
|  | No | 25 (71.43) | 15 (75.00) | 10 (66.67) |  |  |
|  | Yes | 10 (28.57) | 5 (25.00) | 5 (33.33) |  |  |

#### Disease activity (V2)

|  | | | **Group Name** | |  | |
| --- | --- | --- | --- | --- | --- | --- |
| **variable** | **Details** | **total population** | **Program MBSR** | **Classic follow-up and general advice** | **Test** | **p** |
| VAS: physician's assessment of illness (F/NF) (n(%col)) |  | N=36 | n=20 | n=16 | FISHER | 0.190 |
|  | Not Done | 2 (5.56) | 0 (0.00) | 2 (12.50) |  |  |
|  | Done | 34 (94.44) | 20 (100.00) | 14 (87.50) |  |  |
| VAS: physician's assessment of illness |  | N=34 | n=20 | n=14 | STUDENT | 0.156 |
|  | Mean (± SD) | 42.06 (± 21.04) | 37.75 (± 23.20) | 48.21 (± 16.36) |  |  |
|  | Median (Q1;Q3) | 50.00 (30.00 ; 60.00) | 40.00 (20.00 ; 60.00) | 50.00 (30.00 ; 60.00) |  |  |
|  | Median (min;max) | 50.00 (0.00 ; 80.00) | 40.00 (0.00 ; 80.00) | 50.00 (20.00 ; 70.00) |  |  |

#### Auto-questionnaire (V2)

|  | | | **Group Name** | |  | |
| --- | --- | --- | --- | --- | --- | --- |
| **variable** | **Details** | **total population** | **Program MBSR** | **Classic follow-up and general advice** | **Test** | **p** |
| VAS: patient pain assessment F/NF (n(%col)) |  | N=36 | n=20 | n=16 | INVALID | . |
|  | Done | 36 (100.00) | 20 (100.00) | 16 (100.00) |  |  |
| VAS: patient's assessment of pain |  | N=36 | n=20 | n=16 | STUDENT | 0.125 |
|  | Mean (± SD) | 40.75 (± 27.63) | 34.40 (± 27.21) | 48.69 (± 26.89) |  |  |
|  | Median (Q1;Q3) | 45.00 (11.50 ; 60.50) | 32.00 (10.50 ; 60.00) | 57.50 (28.00 ; 64.50) |  |  |
|  | Median (min;max) | 45.00 (0.00 ; 92.00) | 32.00 (0.00 ; 85.00) | 57.50 (0.00 ; 92.00) |  |  |
| VAS: patient assessment of disease activity F/NF (n(%col)) |  | N=36 | n=20 | n=16 | INVALID | . |
|  | Done | 36 (100.00) | 20 (100.00) | 16 (100.00) |  |  |
| VAS: patient's assessment of disease activity |  | N=36 | n=20 | n=16 | STUDENT | 0.610 |
|  | Mean (± SD) | 46.83 (± 27.51) | 44.70 (± 25.73) | 49.50 (± 30.23) |  |  |
|  | Median (Q1;Q3) | 44.50 (24.00 ; 70.00) | 40.00 (25.50 ; 63.50) | 59.50 (24.00 ; 74.50) |  |  |
|  | Median (min;max) | 44.50 (0.00 ; 95.00) | 40.00 (0.00 ; 95.00) | 59.50 (0.00 ; 95.00) |  |  |
| VAS: patient assessment of health status F/NF (n(%col)) |  | N=36 | n=20 | n=16 | INVALID | . |
|  | Done | 36 (100.00) | 20 (100.00) | 16 (100.00) |  |  |
| VAS: patient's assessment of health status |  | N=36 | n=20 | n=16 | STUDENT | 0.243 |
|  | Mean (± SD) | 58.06 (± 24.98) | 62.45 (± 23.23) | 52.56 (± 26.73) |  |  |
|  | Median (Q1;Q3) | 60.00 (37.00 ; 79.00) | 65.50 (45.00 ; 82.00) | 50.00 (35.00 ; 70.00) |  |  |
|  | Median (min;max) | 60.00 (6.00 ; 100.00) | 65.50 (20.00 ; 95.00) | 50.00 (6.00 ; 100.00) |  |  |
| WOMAC F/NF (n(%col)) |  | N=36 | n=20 | n=16 | INVALID | . |
|  | Done | 36 (100.00) | 20 (100.00) | 16 (100.00) |  |  |
| SF36 F/NF (n(%col)) |  | N=36 | n=20 | n=16 | INVALID | . |
|  | Done | 36 (100.00) | 20 (100.00) | 16 (100.00) |  |  |
| HAD F/NF (n(%col)) |  | N=36 | n=20 | n=16 | INVALID | . |
|  | Done | 36 (100.00) | 20 (100.00) | 16 (100.00) |  |  |
| FFMQ F/NF (n(%col)) |  | N=36 | n=20 | n=16 | INVALID | . |
|  | Done | 36 (100.00) | 20 (100.00) | 16 (100.00) |  |  |
| Meditation practice Y/N (n(%col)) |  | N=24 | n=20 | n=4 | INVALID | . |
|  | Done | 24 (100.00) | 20 (100.00) | 4 (100.00) |  |  |

#### Indice WOMAC (V2)

|  | | | **Group Name** | |  | |
| --- | --- | --- | --- | --- | --- | --- |
| **variable** | **Details** | **total population** | **Program MBSR** | **Classic follow-up and general advice** | **test** | **p** |
| Womac : Score pain |  | N=35 | n=20 | n=15 | STUDENT | 0.809 |
|  | Mean (± SD) | 38.71 (± 19.72) | 38.00 (± 19.76) | 39.67 (± 20.31) |  |  |
|  | Median (Q1;Q3) | 35.00 (25.00 ; 55.00) | 35.00 (27.50 ; 55.00) | 45.00 (20.00 ; 55.00) |  |  |
|  | Median (min;max) | 35.00 (0.00 ; 80.00) | 35.00 (0.00 ; 70.00) | 45.00 (0.00 ; 80.00) |  |  |
| WOMAC : Score function |  | N=32 | n=18 | n=14 | STUDENT | 0.869 |
|  | Mean (± SD) | 39.02 (± 20.30) | 38.48 (± 19.57) | 39.71 (± 21.93) |  |  |
|  | Median (Q1;Q3) | 39.71 (26.47 ; 54.41) | 39.71 (26.47 ; 54.41) | 43.39 (26.47 ; 54.41) |  |  |
|  | Median (min;max) | 39.71 (0.00 ; 79.41) | 39.71 (0.00 ; 66.18) | 43.39 (0.00 ; 79.41) |  |  |
| WOMAC : Score stiffness |  | N=36 | n=20 | n=16 | STUDENT | 0.279 |
|  | Mean (± SD) | 44.10 (± 21.23) | 40.63 (± 16.66) | 48.44 (± 25.77) |  |  |
|  | Median (Q1;Q3) | 43.75 (25.00 ; 56.25) | 37.50 (25.00 ; 50.00) | 50.00 (31.25 ; 68.75) |  |  |
|  | Median (min;max) | 43.75 (0.00 ; 87.50) | 37.50 (12.50 ; 75.00) | 50.00 (0.00 ; 87.50) |  |  |
| WOMAC : Score Total |  | N=32 | n=18 | n=14 | STUDENT | 0.670 |
|  | Mean (± SD) | 41.99 (± 19.02) | 40.70 (± 16.49) | 43.65 (± 22.41) |  |  |
|  | Median (Q1;Q3) | 44.95 (29.22 ; 54.39) | 43.24 (30.29 ; 52.01) | 48.34 (27.99 ; 56.62) |  |  |
|  | Median (min;max) | 44.95 (0.00 ; 82.30) | 43.24 (8.33 ; 64.61) | 48.34 (0.00 ; 82.30) |  |  |

#### SF-36 health status questionnaire without inconsistencies (V2)

|  | | | **Group Name** | |  | |
| --- | --- | --- | --- | --- | --- | --- |
| **variable** | **Details** | **total population** | **Program MBSR** | **Classic follow-up and general advice** | **test** | **p** |
| Score : Physical Activity(i) |  | N=36 | n=20 | n=16 | STUDENT | 0.973 |
|  | Mean (± SD) | 57.35 (± 25.18) | 57.48 (± 25.43) | 57.19 (± 25.69) |  |  |
|  | Median (Q1;Q3) | 55.00 (35.00 ; 77.50) | 56.67 (35.00 ; 80.00) | 55.00 (45.00 ; 67.50) |  |  |
|  | Median (min;max) | 55.00 (6.25 ; 100.00) | 56.67 (6.25 ; 95.00) | 55.00 (10.00 ; 100.00) |  |  |
| Score : Limitations due to physical condition(i) |  | N=36 | n=20 | n=16 | WMW | 0.351 |
|  | Mean (± SD) | 49.54 (± 44.00) | 56.25 (± 43.59) | 41.15 (± 44.46) |  |  |
|  | Median (Q1;Q3) | 41.67 (0.00 ; 100.00) | 75.00 (12.50 ; 100.00) | 29.17 (0.00 ; 100.00) |  |  |
|  | Median (min;max) | 41.67 (0.00 ; 100.00) | 75.00 (0.00 ; 100.00) | 29.17 (0.00 ; 100.00) |  |  |
| Score : Limitations due to psychological state(i) |  | N=36 | n=20 | n=16 | WMW | 0.143 |
|  | Mean (± SD) | 56.48 (± 48.35) | 66.67 (± 47.14) | 43.75 (± 48.26) |  |  |
|  | Median (Q1;Q3) | 100.00 (0.00 ; 100.00) | 100.00 (0.00 ; 100.00) | 16.67 (0.00 ; 100.00) |  |  |
|  | Median (min;max) | 100.00 (0.00 ; 100.00) | 100.00 (0.00 ; 100.00) | 16.67 (0.00 ; 100.00) |  |  |
| Score : Life and relationship with others(i) |  | N=36 | n=20 | n=16 | STUDENT | 0.065 |
|  | Mean (± SD) | 64.58 (± 26.64) | 71.88 (± 21.41) | 55.47 (± 30.27) |  |  |
|  | Median (Q1;Q3) | 62.50 (50.00 ; 87.50) | 75.00 (50.00 ; 87.50) | 56.25 (25.00 ; 81.25) |  |  |
|  | Median (min;max) | 62.50 (12.50 ; 100.00) | 75.00 (37.50 ; 100.00) | 56.25 (12.50 ; 100.00) |  |  |
| Score : Physical pain(i) |  | N=36 | n=20 | n=16 | STUDENT | 0.940 |
|  | Mean (± SD) | 47.75 (± 26.12) | 48.05 (± 25.89) | 47.38 (± 27.25) |  |  |
|  | Median (Q1;Q3) | 46.00 (31.00 ; 62.00) | 51.00 (31.50 ; 62.00) | 41.00 (26.50 ; 72.00) |  |  |
|  | Median (min;max) | 46.00 (0.00 ; 100.00) | 51.00 (0.00 ; 100.00) | 41.00 (12.00 ; 100.00) |  |  |
| Score : Psychological health(i) |  | N=36 | n=20 | n=16 | WMW | 0.044 |
|  | Mean (± SD) | 65.83 (± 17.87) | 71.70 (± 13.74) | 58.50 (± 20.07) |  |  |
|  | Median (Q1;Q3) | 72.00 (51.00 ; 80.00) | 74.00 (70.00 ; 80.00) | 56.00 (40.00 ; 76.00) |  |  |
|  | Median (min;max) | 72.00 (24.00 ; 92.00) | 74.00 (44.00 ; 92.00) | 56.00 (24.00 ; 92.00) |  |  |
| Score : Vitality(i) |  | N=36 | n=20 | n=16 | STUDENT | 0.135 |
|  | Mean (± SD) | 50.32 (± 21.23) | 55.08 (± 17.58) | 44.38 (± 24.35) |  |  |
|  | Median (Q1;Q3) | 53.33 (37.50 ; 65.00) | 55.00 (42.50 ; 65.83) | 42.50 (30.00 ; 65.00) |  |  |
|  | Median (min;max) | 53.33 (5.00 ; 85.00) | 55.00 (20.00 ; 85.00) | 42.50 (5.00 ; 85.00) |  |  |
| Score : Perceived health(i) |  | N=35 | n=20 | n=15 | STUDENT | 0.795 |
|  | Mean (± SD) | 57.60 (± 24.47) | 58.55 (± 20.45) | 56.33 (± 29.72) |  |  |
|  | Median (Q1;Q3) | 62.00 (40.00 ; 77.00) | 59.50 (41.00 ; 72.00) | 67.00 (20.00 ; 82.00) |  |  |
|  | Median (min;max) | 62.00 (5.00 ; 100.00) | 59.50 (22.00 ; 100.00) | 67.00 (5.00 ; 97.00) |  |  |
| Aggregate physical score(i) |  | N=35 | n=20 | n=15 | STUDENT | 0.685 |
|  | Mean (± SD) | 39.22 (± 10.92) | 38.56 (± 10.48) | 40.10 (± 11.79) |  |  |
|  | Median (Q1;Q3) | 39.68 (29.10 ; 47.28) | 41.69 (28.68 ; 46.53) | 36.65 (32.64 ; 50.19) |  |  |
|  | Median (min;max) | 39.68 (19.62 ; 61.57) | 41.69 (19.62 ; 57.22) | 36.65 (22.96 ; 61.57) |  |  |
| Aggregate psychic score(i) |  | N=35 | n=20 | n=15 | WMW | 0.113 |
|  | Mean (± SD) | 46.30 (± 12.02) | 50.02 (± 8.59) | 41.34 (± 14.30) |  |  |
|  | Median (Q1;Q3) | 52.70 (36.34 ; 56.11) | 53.17 (43.61 ; 56.34) | 40.95 (26.63 ; 56.11) |  |  |
|  | Median (min;max) | 52.70 (22.05 ; 62.26) | 53.17 (31.31 ; 58.79) | 40.95 (22.05 ; 62.26) |  |  |
| Sum of the scores of the 8 dim of the SF36(/800)(i) |  | N=35 | n=20 | n=15 | STUDENT | 0.273 |
|  | Mean (± SD) | 454.03 (± 194.36) | 485.65 (± 173.14) | 411.86 (± 218.46) |  |  |
|  | Median (Q1;Q3) | 432.50 (268.75 ; 637.50) | 494.17 (316.92 ; 631.50) | 424.67 (208.00 ; 651.50) |  |  |
|  | Median (min;max) | 432.50 (124.50 ; 772.00) | 494.17 (231.00 ; 772.00) | 424.67 (124.50 ; 755.00) |  |  |
| Average of the scores of the 8 dim of the SF36 (i) |  | N=35 | n=20 | n=15 | STUDENT | 0.273 |
|  | Mean (± SD) | 56.75 (± 24.30) | 60.71 (± 21.64) | 51.48 (± 27.31) |  |  |
|  | Median (Q1;Q3) | 54.06 (33.59 ; 79.69) | 61.77 (39.61 ; 78.94) | 53.08 (26.00 ; 81.44) |  |  |
|  | Median (min;max) | 54.06 (15.56 ; 96.50) | 61.77 (28.88 ; 96.50) | 53.08 (15.56 ; 94.38) |  |  |

#### HADS (V2)

|  | | | **Group Name** | |  | |
| --- | --- | --- | --- | --- | --- | --- |
| **variable** | **Details** | **total population** | **Program MBSR** | **Classic follow-up and general advice** | **test** | **p** |
| HADS : Score Anxiety |  | N=36 | n=20 | n=16 | WMW | 0.084 |
|  | Mean (± SD) | 7.17 (± 4.88) | 6.15 (± 4.43) | 8.44 (± 5.27) |  |  |
|  | Median (Q1;Q3) | 6.00 (3.50 ; 8.50) | 5.50 (3.00 ; 7.00) | 7.50 (5.00 ; 9.50) |  |  |
|  | Median (min;max) | 6.00 (1.00 ; 20.00) | 5.50 (1.00 ; 18.00) | 7.50 (2.00 ; 20.00) |  |  |
| HADS : Score Dwpression |  | N=36 | n=20 | n=16 | WMW | 0.211 |
|  | Mean (± SD) | 4.19 (± 3.42) | 3.50 (± 2.89) | 5.06 (± 3.91) |  |  |
|  | Median (Q1;Q3) | 4.00 (1.50 ; 6.00) | 2.50 (1.50 ; 5.50) | 4.50 (2.50 ; 6.50) |  |  |
|  | Median (min;max) | 4.00 (0.00 ; 16.00) | 2.50 (0.00 ; 11.00) | 4.50 (0.00 ; 16.00) |  |  |
| HADS : Score Total |  | N=36 | n=20 | n=16 | WMW | 0.094 |
|  | Mean (± SD) | 11.36 (± 7.62) | 9.65 (± 6.92) | 13.50 (± 8.12) |  |  |
|  | Median (Q1;Q3) | 10.50 (5.50 ; 14.00) | 7.00 (5.00 ; 12.00) | 11.50 (9.50 ; 15.00) |  |  |
|  | Median (min;max) | 10.50 (2.00 ; 30.00) | 7.00 (2.00 ; 29.00) | 11.50 (3.00 ; 30.00) |  |  |

#### FFMQ (V2)

|  | | | **Group Name** | |  | |
| --- | --- | --- | --- | --- | --- | --- |
| **variable** | **Details** | **total population** | **Program MBSR** | **Classic follow-up and general advice** | **test** | **p** |
| FFMQ : Observation Factor |  | N=36 | n=20 | n=16 | STUDENT | 0.010 |
|  | Mean (± SD) | 29.97 (± 5.68) | 32.10 (± 4.48) | 27.31 (± 6.02) |  |  |
|  | Median (Q1;Q3) | 31.00 (24.50 ; 35.00) | 32.50 (30.50 ; 35.00) | 27.00 (22.50 ; 33.00) |  |  |
|  | Median (min;max) | 31.00 (18.00 ; 39.00) | 32.50 (24.00 ; 39.00) | 27.00 (18.00 ; 37.00) |  |  |
| FFMQ: Description Factor Experience |  | N=36 | n=20 | n=16 | WMW | 0.836 |
|  | Mean (± SD) | 30.58 (± 6.40) | 31.05 (± 4.72) | 30.00 (± 8.17) |  |  |
|  | Median (Q1;Q3) | 32.00 (25.00 ; 36.00) | 32.00 (28.50 ; 34.00) | 34.50 (23.50 ; 36.00) |  |  |
|  | Median (min;max) | 32.00 (16.00 ; 40.00) | 32.00 (22.00 ; 38.00) | 34.50 (16.00 ; 40.00) |  |  |
| FFMQ: Mindfulness Factor |  | N=35 | n=20 | n=15 | STUDENT | 0.762 |
|  | Mean (± SD) | 29.80 (± 6.61) | 30.10 (± 6.60) | 29.40 (± 6.84) |  |  |
|  | Median (Q1;Q3) | 30.00 (25.00 ; 36.00) | 30.50 (25.50 ; 35.50) | 30.00 (25.00 ; 36.00) |  |  |
|  | Median (min;max) | 30.00 (18.00 ; 40.00) | 30.50 (20.00 ; 40.00) | 30.00 (18.00 ; 37.00) |  |  |
| FFMQ : Factor Evt prive |  | N=36 | n=20 | n=16 | STUDENT | 0.258 |
|  | Mean (± SD) | 22.22 (± 4.85) | 23.05 (± 5.24) | 21.19 (± 4.26) |  |  |
|  | Median (Q1;Q3) | 22.00 (19.50 ; 26.00) | 24.00 (19.00 ; 26.50) | 21.00 (20.00 ; 24.50) |  |  |
|  | Median (min;max) | 22.00 (11.00 ; 32.00) | 24.00 (12.00 ; 32.00) | 21.00 (11.00 ; 27.00) |  |  |
| FFMQ : Non-judgment factor |  | N=36 | n=20 | n=16 | STUDENT | 0.136 |
|  | Mean (± SD) | 29.08 (± 7.02) | 30.65 (± 6.36) | 27.13 (± 7.50) |  |  |
|  | Median (Q1;Q3) | 29.50 (24.00 ; 35.00) | 30.50 (26.00 ; 36.50) | 27.00 (23.00 ; 31.50) |  |  |
|  | Median (min;max) | 29.50 (14.00 ; 40.00) | 30.50 (19.00 ; 40.00) | 27.00 (14.00 ; 40.00) |  |  |
| FFMQ : Mindfulness |  | N=35 | n=20 | n=15 | STUDENT | 0.083 |
|  | Mean (± SD) | 141.29 (± 22.31) | 146.95 (± 17.32) | 133.73 (± 26.37) |  |  |
|  | Median (Q1;Q3) | 145.00 (126.00 ; 159.00) | 149.00 (139.50 ; 158.00) | 132.00 (119.00 ; 161.00) |  |  |
|  | Median (min;max) | 145.00 (87.00 ; 177.00) | 149.00 (113.00 ; 177.00) | 132.00 (87.00 ; 170.00) |  |  |

#### Daily meditation practive at 3 months self-questionnaire

|  | | | **Group Name** | |  | |
| --- | --- | --- | --- | --- | --- | --- |
| **variable** | **Details** | **total population** | **Program MBSR** | **Classic follow-up and general advice** | **Test** | **p** |
| Meditation sessions Q 1 (n(%col)) |  | N=8 | n=6 | n=2 | INVALID | . |
|  | Yes | 8 (100.00) | 6 (100.00) | 2 (100.00) |  |  |
| Meditation sessions Q 2 (n(%col)) |  | N=7 | n=7 | n=0 | INVALID | . |
|  | Yes | 7 (100.00) | 7 (100.00) | 0 (0.00) |  |  |
| Meditation sessions Q 3 (n(%col)) |  | N=4 | n=4 | n=0 | INVALID | . |
|  | Yes | 4 (100.00) | 4 (100.00) | 0 (0.00) |  |  |
| Meditation sessions Q 4 (n(%col)) |  | N=2 | n=2 | n=0 | INVALID | . |
|  | Yes | 2 (100.00) | 2 (100.00) | 0 (0.00) |  |  |
| Meditation sessions Q 5 (n(%col)) |  | N=1 | n=1 | n=0 | INVALID | . |
|  | Yes | 1 (100.00) | 1 (100.00) | 0 (0.00) |  |  |
| Reason for meditation NF (n(%col)) |  | N=12 | n=10 | n=2 | FISHER | 1.000 |
|  | - EASEMENT TO BE ACCEPTED ON THE ORGANIZATION OF THE DAY - INCOMPLETE WAITING | 1 (8.33) | 1 (10.00) | 0 (0.00) |  |  |
|  | STOPPED PRACTICING MEDITATION. SHE STARTED TO PRACTICE SPORTS QUITE A BIT. | 1 (8.33) | 1 (10.00) | 0 (0.00) |  |  |
|  | CLASSIC ARM | 1 (8.33) | 0 (0.00) | 1 (50.00) |  |  |
|  | PAIN | 1 (8.33) | 1 (10.00) | 0 (0.00) |  |  |
|  | ONLY WHEN NEEDED | 1 (8.33) | 1 (10.00) | 0 (0.00) |  |  |
|  | BIG PHYSICAL AND PSYCHIC FATIGUE | 1 (8.33) | 1 (10.00) | 0 (0.00) |  |  |
|  | LACK OF MOTIVATION AND THE DAILY LIFE THAT DOESN'T HELP A LOT TO MEDIATE | 1 (8.33) | 1 (10.00) | 0 (0.00) |  |  |
|  | COULD NOT PRACTICE BECAUSE OF FAMILY PROBLEMS | 1 (8.33) | 0 (0.00) | 1 (50.00) |  |  |
|  | COULD NOT PRACTICE FOR 3 WEEKS BECAUSE OF A TRIP | 1 (8.33) | 1 (10.00) | 0 (0.00) |  |  |
|  | NO TIME | 1 (8.33) | 1 (10.00) | 0 (0.00) |  |  |
|  | BUSY WITH MY ACTIVITIES, OR TRAVEL CONTEXT WHERE IT WAS MORE DIFFICULT TO PRACTICE | 1 (8.33) | 1 (10.00) | 0 (0.00) |  |  |
|  | RESUMPTION OF SPORT | 1 (8.33) | 1 (10.00) | 0 (0.00) |  |  |

|  | | | **Group Name** | |  | |
| --- | --- | --- | --- | --- | --- | --- |
| **variable** | **Details** | **total population** | **Program MBSR** | **Classic follow-up and general advice** | **Test** | **p** |
| V2_Recommended daily meditation practice (n(% col)) |  | N=22 | n=20 | n=2 | FISHER | 0.619 |
|  | 0 to 1 time / week | 8 (36.36) | 6 (30.00) | 2 (100.00) |  |  |
|  | 2 to 3 times / week | 7 (31.82) | 7 (35.00) | 0 (0.00) |  |  |
|  | 4 to 5 times / week | 4 (18.18) | 4 (20.00) | 0 (0.00) |  |  |
|  | Every day of the week, but incomplete in terms of what was said | 2 (9.09) | 2 (10.00) | 0 (0.00) |  |  |
|  | Every day of the week is fairly complete | 1 (4.55) | 1 (5.00) | 0 (0.00) |  |  |

### End of the study

|  | | | **Group Name** | |  | |
| --- | --- | --- | --- | --- | --- | --- |
| **variable** | **Details** | **total population** | **Program MBSR** | **Classic follow-up and general advice** | **Test** | **p** |
| Study continued to completion (ON) (n(%col)) |  | N=40 | n=20 | n=20 | FISHER | 0.106 |
|  | No | 4 (10.00) | 0 (0.00) | 4 (20.00) |  |  |
|  | Yes | 36 (90.00) | 20 (100.00) | 16 (80.00) |  |  |
| Reason for leaving study (n(%col)) |  | N=4 | n=0 | n=4 | INVALID | . |
|  | Patient refuses to continue the study | 3 (75.00) | 0 (0.00) | 3 (75.00) |  |  |
|  | Lost sight of | 1 (25.00) | 0 (0.00) | 1 (25.00) |  |  |
| Reason for refusal to continue study (n(%col)) |  | N=2 | n=0 | n=2 | INVALID | . |
|  | NO TIME. LAUNCH OF HER ACTIVITY (HYPNOTHERAPY PRACTICE) | 1 (50.00) | 0 (0.00) | 1 (50.00) |  |  |
|  | PATIENT NO LONGER FEELS CONCERNED BECAUSE NO MORE PAIN. RE-NOTICED TO HER PARENTS. | 1 (50.00) | 0 (0.00) | 1 (50.00) |  |  |
| Other reason for leaving school (n(%col)) |  | N=1 | n=0 | n=1 | INVALID | . |
|  | PATIENT DID NOT COME EITHER V1 OR V2. NO ANSWER TO PHONE OR EMAIL | 1 (100.00) | 0 (0.00) | 1 (100.00) |  |  |

### SUMMARY OF ADVERSE EVENTS

|  | | | **Group Name** | |  | |
| --- | --- | --- | --- | --- | --- | --- |
| **variable** | **Details** | **total population** | **Program MBSR** | **Classic follow-up and general advice** | **Test** | **p** |
| AR: Standard report (n(%col)) |  | N=10 | n=6 | n=4 | FISHER | 1.000 |
|  | Initial | 7 (70.00) | 4 (66.67) | 3 (75.00) |  |  |
|  | Follow-up | 3 (30.00) | 2 (33.33) | 1 (25.00) |  |  |
| AE: Follow-up number (n(%col)) |  | N=3 | n=2 | n=1 | INVALID | . |
|  | 2 | 3 (100.00) | 2 (100.00) | 1 (100.00) |  |  |
| AE: Study exit (ON) (n(%col)) |  | N=10 | n=6 | n=4 | INVALID | . |
|  | No | 10 (100.00) | 6 (100.00) | 4 (100.00) |  |  |
| AE: Main symptoms (n(%col)) |  | N=10 | n=6 | n=4 | FISHER | 1.000 |
|  | SEVERE PAIN, COULD NOT WALK. EXTERNAL AND INTERNAL MENISECTOMIES | 1 (10.00) | 1 (16.67) | 0 (0.00) |  |  |
|  | GASTROSCOPY AND COLONOSCOPY SCHEDULED FOR CONTROL OF EBO | 1 (10.00) | 0 (0.00) | 1 (25.00) |  |  |
|  | GENE, PAIN FOR MORTON SYNDROME | 1 (10.00) | 0 (0.00) | 1 (25.00) |  |  |
|  | GRIPPE | 1 (10.00) | 1 (16.67) | 0 (0.00) |  |  |
|  | CATARACT SURGERY AND IMPLANTS | 1 (10.00) | 1 (16.67) | 0 (0.00) |  |  |
|  | CATARACT SURGERY ON THE LEFT EYE AND LENS PLACEMENT | 1 (10.00) | 1 (16.67) | 0 (0.00) |  |  |
|  | RHIZARTHROSIS AND CARPAL TUNNEL SURGERY ON RIGHT HAND | 1 (10.00) | 1 (16.67) | 0 (0.00) |  |  |
|  | PNEUMOPATHY | 1 (10.00) | 0 (0.00) | 1 (25.00) |  |  |
|  | RHIZARTHROSIS RIGHT THUMB | 1 (10.00) | 1 (16.67) | 0 (0.00) |  |  |
|  | CORONARY SYNDROME | 1 (10.00) | 0 (0.00) | 1 (25.00) |  |  |
| IE: Diagnosis retained (n(%col)) |  | N=10 | n=6 | n=4 | FISHER | 1.000 |
|  | SURGERY FOR MORTON'S NEUROMA RIGHT FOOT | 1 (10.00) | 0 (0.00) | 1 (25.00) |  |  |
|  | GASTROSCOPY | 1 (10.00) | 0 (0.00) | 1 (25.00) |  |  |
|  | GRIPPE | 1 (10.00) | 1 (16.67) | 0 (0.00) |  |  |
|  | EXTERNAL AND INTERNAL MENISECTOMIES LEFT KNEE UNDER ARTHROSCOPY | 1 (10.00) | 1 (16.67) | 0 (0.00) |  |  |
|  | RHIZARTHROSIS AND CARPAL TUNNEL SURGERY | 1 (10.00) | 1 (16.67) | 0 (0.00) |  |  |
|  | CATARACT SURGERY | 1 (10.00) | 1 (16.67) | 0 (0.00) |  |  |
|  | CATARACT SURGERY ON THE RIGHT EYE AND IMPLANTATION OF IMPLANTS | 1 (10.00) | 1 (16.67) | 0 (0.00) |  |  |
|  | PNEUMOPATHY | 1 (10.00) | 0 (0.00) | 1 (25.00) |  |  |
|  | TRAPEZOMETACARPAL PROSTHESIS | 1 (10.00) | 1 (16.67) | 0 (0.00) |  |  |
|  | CORONARY SYNDROME. LATERAL AKINESIA WITHOUT OEDEMA AND WITHOUT SIGNS IN FAVOUR | 1 (10.00) | 0 (0.00) | 1 (25.00) |  |  |
| AEs: SAEs (ON) (n(%col)) |  | N=10 | n=6 | n=4 | FISHER | 0.133 |
|  | No | 8 (80.00) | 6 (100.00) | 2 (50.00) |  |  |
|  | Yes | 2 (20.00) | 0 (0.00) | 2 (50.00) |  |  |
| AE: Hospitalization/extension (ON) (n(%col)) |  | N=2 | n=0 | n=2 | INVALID | . |
|  | Yes | 2 (100.00) | 0 (0.00) | 2 (100.00) |  |  |
| AE: Intensity (n(%col)) |  | N=10 | n=6 | n=4 | FISHER | 0.333 |
|  | Grade 1: Mild | 4 (40.00) | 2 (33.33) | 2 (50.00) |  |  |
|  | Grade 2: Moderate | 5 (50.00) | 4 (66.67) | 1 (25.00) |  |  |
|  | Grade 4: Severe | 1 (10.00) | 0 (0.00) | 1 (25.00) |  |  |
| AE: Course (n(%col)) |  | N=10 | n=6 | n=4 | FISHER | 0.467 |
|  | Event in progress | 2 (20.00) | 2 (33.33) | 0 (0.00) |  |  |
|  | Resolution without sequelae | 7 (70.00) | 4 (66.67) | 3 (75.00) |  |  |
|  | Resolution with sequelae | 1 (10.00) | 0 (0.00) | 1 (25.00) |  |  |
| AE: Nature of sequelae (n(%col)) |  | N=1 | n=0 | n=1 | INVALID | . |
|  | DISCHARGED FROM HOSPITAL ON 24/12, BUT CONTINUES EXPLORATIONS IN HOSPITAL | 1 (100.00) | 0 (0.00) | 1 (100.00) |  |  |
| AE: Accountability for experimental procedure (ON) (n(%col)) |  | N=10 | n=6 | n=4 | INVALID | . |
|  | No | 10 (100.00) | 6 (100.00) | 4 (100.00) |  |  |
| AE: Attributable study protocol (ON) (n(%col)) |  | N=10 | n=6 | n=4 | INVALID | . |
|  | No | 10 (100.00) | 6 (100.00) | 4 (100.00) |  |  |
| AEs: Attributable to other concomitant illness (NA) (n(%col)) |  | N=10 | n=6 | n=4 | FISHER | 0.571 |
|  | No | 4 (40.00) | 3 (50.00) | 1 (25.00) |  |  |
|  | Yes | 6 (60.00) | 3 (50.00) | 3 (75.00) |  |  |
| AEs: Attributable disease progression (ON) (n(%col)) |  | N=10 | n=6 | n=4 | FISHER | 1.000 |
|  | No | 9 (90.00) | 5 (83.33) | 4 (100.00) |  |  |
|  | Yes | 1 (10.00) | 1 (16.67) | 0 (0.00) |  |  |
| AEs: Attributable to other concomitant therapy (ON) (n(%col)) |  | N=10 | n=6 | n=4 | INVALID | . |
|  | No | 10 (100.00) | 6 (100.00) | 4 (100.00) |  |  |
| AE: Attributable to other (ON) (n(%col)) |  | N=10 | n=6 | n=4 | FISHER | 1.000 |
|  | No | 8 (80.00) | 5 (83.33) | 3 (75.00) |  |  |
|  | Yes | 2 (20.00) | 1 (16.67) | 1 (25.00) |  |  |
| AE: Accountability other (precision) (n(%col)) |  | N=2 | n=1 | n=1 | FISHER | 1.000 |
|  | DISEASE SEASON WINTER | 1 (50.00) | 0 (0.00) | 1 (100.00) |  |  |
|  | RHIZARTHROSIS OF THE THUMB | 1 (50.00) | 1 (100.00) | 0 (0.00) |  |  |
| AE: Number (n(%col)) |  | N=200 | n=100 | n=100 | CHI2 | 1.000 |
|  | 1 | 40 (20.00) | 20 (20.00) | 20 (20.00) |  |  |
|  | 2 | 40 (20.00) | 20 (20.00) | 20 (20.00) |  |  |
|  | 3 | 40 (20.00) | 20 (20.00) | 20 (20.00) |  |  |
|  | 4 | 40 (20.00) | 20 (20.00) | 20 (20.00) |  |  |
|  | 5 | 40 (20.00) | 20 (20.00) | 20 (20.00) |  |  |
| SAE: Experimental procedure 1: Description (n(%col)) |  | N=2 | n=0 | n=2 | INVALID | . |
|  | NO PROCEDURE (CLASSIC GROUP) | 1 (50.00) | 0 (0.00) | 1 (50.00) |  |  |
|  | NO PROCEDURE (CLASSIC GROUP) | 1 (50.00) | 0 (0.00) | 1 (50.00) |  |  |
| SAE: Experimental procedure 1: Accountability (n(%col)) |  | N=2 | n=0 | n=2 | INVALID | . |
|  | Unbound | 2 (100.00) | 0 (0.00) | 2 (100.00) |  |  |
| SAE: Treatment 1: Description (n(%col)) |  | N=2 | n=0 | n=2 | INVALID | . |
|  | ANTI-PLATELET AGGREGATION AGENT | 1 (50.00) | 0 (0.00) | 1 (50.00) |  |  |
|  | KCL 20 | 1 (50.00) | 0 (0.00) | 1 (50.00) |  |  |
| SAE: Treatment 1: Dose/unit (n(%col)) |  | N=1 | n=0 | n=1 | INVALID | . |
|  | 10 ML | 1 (100.00) | 0 (0.00) | 1 (100.00) |  |  |
| SAE: Treatment 1: Route (n(%col)) |  | N=1 | n=0 | n=1 | INVALID | . |
|  | IV | 1 (100.00) | 0 (0.00) | 1 (100.00) |  |  |
| SAE: Treatment 1: Indication (n(%col)) |  | N=2 | n=0 | n=2 | INVALID | . |
|  | ANTIAGGREGANT | 1 (50.00) | 0 (0.00) | 1 (50.00) |  |  |
|  | NUTRITION HYDRATION | 1 (50.00) | 0 (0.00) | 1 (50.00) |  |  |
| SAE: Treatment 2: Description (n(%col)) |  | N=1 | n=0 | n=1 | INVALID | . |
|  | NACL 0.9 | 1 (100.00) | 0 (0.00) | 1 (100.00) |  |  |
| SAE: Treatment 2: Dose/unit (n(%col)) |  | N=1 | n=0 | n=1 | INVALID | . |
|  | 500ML | 1 (100.00) | 0 (0.00) | 1 (100.00) |  |  |
| SAE: Treatment 2: Route (n(%col)) |  | N=1 | n=0 | n=1 | INVALID | . |
|  | INTRAVENEUS | 1 (100.00) | 0 (0.00) | 1 (100.00) |  |  |
| SAE: Treatment 2: Indication (n(%col)) |  | N=1 | n=0 | n=1 | INVALID | . |
|  | NUTRITION HYDRATION | 1 (100.00) | 0 (0.00) | 1 (100.00) |  |  |
| SAE: Treatment 3: Description (n(%col)) |  | N=1 | n=0 | n=1 | INVALID | . |
|  | DIFFU K | 1 (100.00) | 0 (0.00) | 1 (100.00) |  |  |
| SAE: Treatment 3: Dose/unit (n(%col)) |  | N=1 | n=0 | n=1 | INVALID | . |
|  | 600 MG | 1 (100.00) | 0 (0.00) | 1 (100.00) |  |  |
| SAE: Treatment 3: Route (n(%col)) |  | N=1 | n=0 | n=1 | INVALID | . |
|  | ORAL | 1 (100.00) | 0 (0.00) | 1 (100.00) |  |  |
| SAE: Treatment #3: Indication (n(%col)) |  | N=1 | n=0 | n=1 | INVALID | . |
|  | 6 G PER DAY | 1 (100.00) | 0 (0.00) | 1 (100.00) |  |  |
| SAE: Relevant medical history (n(%col)) |  | N=1 | n=0 | n=1 | INVALID | . |
|  | EXAMINATION FINDINGS SHOW LATERAL AKINESIA WITHOUT S-OEDEMA | 1 (100.00) | 0 (0.00) | 1 (100.00) |  |  |
| SAE: Comments (n(%col)) |  | N=1 | n=0 | n=1 | INVALID | . |
|  | NO CONSULTATION PLANNED AFTER HOSPITALISATION NEXT COLONOSCOPY IN 3 DAYS | 1 (100.00) | 0 (0.00) | 1 (100.00) |  |  |

## Primary evaluation criterion

Improvement in WOMAC pain score between initiation (V0) and end of program (V1)

|  | | | **Group Name** | |  | |
| --- | --- | --- | --- | --- | --- | --- |
| **variable** | **Details** | **total population** | **Program MBSR** | **Classic follow-up and general advice** | **test** | **p** |
| Change between Tfin and Tstart of the pain score (Womac) (V1-V0) |  | N=28 | n=16 | n=12 | STUDENT | 0.682 |
|  | Moyenne (± ET) | -6.61 (± 18.81) | -5.31 (± 14.55) | -8.33 (± 23.96) |  |  |
|  | Médiane (Q1;Q3) | -5.00 (-15.00 ; 5.00) | -5.00 (-12.50 ; 0.00) | -7.50 (-27.50 ; 12.50) |  |  |
|  | Médiane (min;max) | -5.00 (-45.00 ; 30.00) | -5.00 (-30.00 ; 20.00) | -7.50 (-45.00 ; 30.00) |  |  |

## SECONDARY ASSESSMENT CRITERIA

### (V1-V0) : VARIATION BETWEEN INITIATION AND TERMINATION OF THE PROGRAM

#### Score pain (EVA/100)

|  | | | **Group Name** | |  | |
| --- | --- | --- | --- | --- | --- | --- |
| **variable** | **Details** | **total population** | **Program MBSR** | **Classic follow-up and general advice** | **test** | **p** |
| Variation between Tfin and Tbegin of the pain score felt by the patient |  | N=36 | n=20 | n=16 | STUDENT | 0.375 |
|  | Moyenne (± ET) | -19.31 (± 24.50) | -22.60 (± 22.31) | -15.19 (± 27.15) |  |  |
|  | Médiane (Q1;Q3) | -15.00 (-43.00 ; -0.50) | -17.50 (-45.00 ; -7.00) | -10.00 (-36.50 ; 9.00) |  |  |
|  | Médiane (min;max) | -15.00 (-63.00 ; 19.00) | -17.50 (-61.00 ; 13.00) | -10.00 (-63.00 ; 19.00) |  |  |

#### Score of disease activity (E100)

##### Patient

|  | | | **Group Name** | |  | |
| --- | --- | --- | --- | --- | --- | --- |
| **variable** | **Details** | **total population** | **Program MBSR** | **Classic follow-up and general advice** | **test** | **p** |
| Change between Tfin and Tstart in patient's global assessment of disease activity |  | N=36 | n=20 | n=16 | WMW | 0.381 |
|  | Mean (± SD) | 3.86 (± 95.44) | -14.60 (± 25.64) | 26.94 (± 139.29) |  |  |
|  | Median (Q1;Q3) | -13.50 (-28.00 ; 6.50) | -16.50 (-30.50 ; -0.50) | -6.50 (-24.50 ; 16.00) |  |  |
|  | Median (min;max) | -13.50 (-79.00 ; 537.00) | -16.50 (-79.00 ; 38.00) | -6.50 (-61.00 ; 537.00) |  |  |

##### Doctor

|  | | | **Group Name** | |  | |
| --- | --- | --- | --- | --- | --- | --- |
| **variable** | **Details** | **total population** | **Program MBSR** | **Classic follow-up and general advice** | **test** | **p** |
| Change between Tfin and Tstart in physician's global assessment of disease activity |  | N=32 | n=19 | n=13 | STUDENT | 0.444 |
|  | Mean (± SD) | -9.06 (± 17.43) | -11.05 (± 16.88) | -6.15 (± 18.50) |  |  |
|  | Median (Q1;Q3) | -5.00 (-20.00 ; 2.50) | -10.00 (-20.00 ; 0.00) | 0.00 (-20.00 ; 10.00) |  |  |
|  | Median (min;max) | -5.00 (-50.00 ; 20.00) | -10.00 (-50.00 ; 10.00) | 0.00 (-40.00 ; 20.00) |  |  |

#### WOMAC (V1-V0)

##### Total

|  | | | **Group Name** | |  | |
| --- | --- | --- | --- | --- | --- | --- |
| **variable** | **Details** | **total population** | **Program MBSR** | **Classic follow-up and general advice** | **test** | **p** |
| Variation between T end and T start of score total (Womac) |  | N=19 | n=9 | n=10 | STUDENT | 0.859 |
|  | Mean (± SD) | -5.04 (± 14.01) | -5.67 (± 11.48) | -4.48 (± 16.57) |  |  |
|  | Median (Q1;Q3) | -4.17 (-14.58 ; 6.25) | -5.21 (-8.33 ; 0.00) | -2.08 (-14.58 ; 6.25) |  |  |
|  | Median (min;max) | -4.17 (-33.33 ; 17.71) | -5.21 (-28.12 ; 9.38) | -2.08 (-33.33 ; 17.71) |  |  |

##### Pain

|  | | | **Group Name** | |  | |
| --- | --- | --- | --- | --- | --- | --- |
| **variable** | **Details** | **total population** | **Program MBSR** | **Classic follow-up and general advice** | **test** | **p** |
| Variation between T end and T start of score pain (Womac) |  | N=28 | n=16 | n=12 | STUDENT | 0.682 |
|  | Mean (± SD) | -6.61 (± 18.81) | -5.31 (± 14.55) | -8.33 (± 23.96) |  |  |
|  | Median (Q1;Q3) | -5.00 (-15.00 ; 5.00) | -5.00 (-12.50 ; 0.00) | -7.50 (-27.50 ; 12.50) |  |  |
|  | Median (min;max) | -5.00 (-45.00 ; 30.00) | -5.00 (-30.00 ; 20.00) | -7.50 (-45.00 ; 30.00) |  |  |

##### Function

|  | | | **Group Name** | |  | |
| --- | --- | --- | --- | --- | --- | --- |
| **variable** | **Details** | **total population** | **Program MBSR** | **Classic follow-up and general advice** | **test** | **p** |
| Variation between T end and T start of score function (Womac) |  | N=23 | n=11 | n=12 | STUDENT | 0.685 |
|  | Mean (± SD) | -2.94 (± 14.62) | -1.60 (± 13.31) | -4.17 (± 16.22) |  |  |
|  | Median (Q1;Q3) | -5.88 (-11.76 ; 7.36) | -1.47 (-8.82 ; 10.30) | -5.88 (-16.18 ; 7.35) |  |  |
|  | Median (min;max) | -5.88 (-32.35 ; 19.12) | -1.47 (-29.41 ; 17.65) | -5.88 (-32.35 ; 19.12) |  |  |

##### Stifness

|  | | | **Group Name** | |  | |
| --- | --- | --- | --- | --- | --- | --- |
| **variable** | **Details** | **total population** | **Program MBSR** | **Classic follow-up and general advice** | **test** | **p** |
| Variation between T end and T start of score stiffness (Womac) |  | N=34 | n=19 | n=15 | WMW | 0.872 |
|  | Mean (± SD) | -2.21 (± 19.58) | -2.63 (± 23.04) | -1.67 (± 14.84) |  |  |
|  | Median (Q1;Q3) | 0.00 (-12.50 ; 12.50) | 0.00 (-25.00 ; 12.50) | 0.00 (-12.50 ; 0.00) |  |  |
|  | Median (min;max) | 0.00 (-37.50 ; 50.00) | 0.00 (-37.50 ; 50.00) | 0.00 (-25.00 ; 37.50) |  |  |

#### Reponse OARSI-OMERACT (V1-V0)

|  | | | **Group Name** | |  | |
| --- | --- | --- | --- | --- | --- | --- |
| **variable** | **Details** | **total population** | **Program MBSR** | **Classic follow-up and general advice** | **Test** | **p** |
| Responders OMERACT-OARSI between T end and T start of (n(%col)) |  | N=19 | n=11 | n=8 | FISHER | 1.000 |
|  | No | 7 (36.84) | 4 (36.36) | 3 (37.50) |  |  |
|  | yes | 12 (63.16) | 7 (63.64) | 5 (62.50) |  |  |

#### Score SF-36 (V0-V1)

##### Score global psychic l (PCS)

|  | | | **Group Name** | |  | |
| --- | --- | --- | --- | --- | --- | --- |
| **variable** | **Details** | **total population** | **Program MBSR** | **Classic follow-up and general advice** | **test** | **p** |
| Variation between T end and T start of score aggregate physical PCS (score SF-36) |  | N=34 | n=20 | n=14 | STUDENT | 0.757 |
|  | Mean (± SD) | 2.07 (± 7.36) | 1.73 (± 7.08) | 2.54 (± 7.99) |  |  |
|  | Median (Q1;Q3) | 1.30 (-2.14 ; 6.81) | 0.69 (-2.05 ; 5.23) | 2.15 (-3.19 ; 7.41) |  |  |
|  | Median (min;max) | 1.30 (-11.08 ; 20.99) | 0.69 (-11.08 ; 19.05) | 2.15 (-10.36 ; 20.99) |  |  |

##### Score global psychic (MCS)

|  | | | **Group Name** | |  | |
| --- | --- | --- | --- | --- | --- | --- |
| **variable** | **Details** | **total population** | **Program MBSR** | **Classic follow-up and general advice** | **test** | **p** |
| Variation between T end and T start of score psychic aggregate MCS (score SF-36) |  | N=34 | n=20 | n=14 | STUDENT | 0.597 |
|  | Mean (± SD) | 1.94 (± 8.28) | 2.58 (± 7.89) | 1.03 (± 9.03) |  |  |
|  | Median (Q1;Q3) | 1.15 (-3.01 ; 6.52) | 1.15 (-2.25 ; 6.33) | 0.99 (-3.34 ; 8.97) |  |  |
|  | Median (min;max) | 1.15 (-19.46 ; 22.87) | 1.15 (-12.83 ; 22.87) | 0.99 (-19.46 ; 14.27) |  |  |

##### Question general health (GH)

|  | | | **Group Name** | |  | |
| --- | --- | --- | --- | --- | --- | --- |
| **variable** | **Details** | **total population** | **Program MBSR** | **Classic follow-up and general advice** | **test** | **p** |
| Variation between T end and T start of perceived health (GH) (score SF-36) |  | N=36 | n=20 | n=16 | STUDENT | 0.592 |
|  | Mean (± SD) | 0.92 (± 12.68) | 1.95 (± 12.09) | -0.38 (± 13.66) |  |  |
|  | Median (Q1;Q3) | 0.00 (-6.00 ; 5.38) | 1.50 (-5.00 ; 7.87) | 0.00 (-8.50 ; 5.00) |  |  |
|  | Median (min;max) | 0.00 (-20.00 ; 35.00) | 1.50 (-20.00 ; 35.00) | 0.00 (-20.00 ; 35.00) |  |  |

#### Score HAD

##### Score anxiety

|  | | | **Group Name** | |  | |
| --- | --- | --- | --- | --- | --- | --- |
| **variable** | **Details** | **total population** | **Program MBSR** | **Classic follow-up and general advice** | **test** | **p** |
| Variation between T end and T start of score HAD anxiete |  | N=35 | n=20 | n=15 | STUDENT | 0.177 |
|  | Mean (± SD) | -1.49 (± 3.08) | -2.10 (± 2.38) | -0.67 (± 3.75) |  |  |
|  | Median (Q1;Q3) | -1.00 (-4.00 ; 0.00) | -2.00 (-4.00 ; -0.50) | 0.00 (-4.00 ; 2.00) |  |  |
|  | Median (min;max) | -1.00 (-9.00 ; 5.00) | -2.00 (-6.00 ; 2.00) | 0.00 (-9.00 ; 5.00) |  |  |

##### Score depression

|  | | | **Group Name** | |  | |
| --- | --- | --- | --- | --- | --- | --- |
| **variable** | **Details** | **total population** | **Program MBSR** | **Classic follow-up and general advice** | **test** | **p** |
| Variation between T end and T start of score HAD depression |  | N=35 | n=20 | n=15 | STUDENT | 0.925 |
|  | Mean (± SD) | -0.71 (± 2.53) | -0.75 (± 2.38) | -0.67 (± 2.79) |  |  |
|  | Median (Q1;Q3) | 0.00 (-3.00 ; 1.00) | 0.00 (-3.00 ; 0.00) | 0.00 (-2.00 ; 1.00) |  |  |
|  | Median (min;max) | 0.00 (-7.00 ; 4.00) | 0.00 (-5.00 ; 4.00) | 0.00 (-7.00 ; 4.00) |  |  |

#### Five Facts Mindfulness Questionnaire (FFMQ)

##### Score total (Mindfulness)

|  | | | **Group Name** | |  | |
| --- | --- | --- | --- | --- | --- | --- |
| **variable** | **Details** | **total population** | **Program MBSR** | **Classic follow-up and general advice** | **test** | **p** |
| Variation between T end and T start of score FFMQ total |  | N=29 | n=16 | n=13 | STUDENT | 0.104 |
|  | Mean (± SD) | 6.62 (± 16.01) | 10.75 (± 19.34) | 1.54 (± 8.93) |  |  |
|  | Median (Q1;Q3) | 1.00 (-4.00 ; 14.00) | 8.50 (-2.50 ; 30.50) | -1.00 (-5.00 ; 9.00) |  |  |
|  | Median (min;max) | 1.00 (-21.00 ; 41.00) | 8.50 (-21.00 ; 41.00) | -1.00 (-12.00 ; 20.00) |  |  |

##### Factor observation

|  | | | **Group Name** | |  | |
| --- | --- | --- | --- | --- | --- | --- |
| **variable** | **Details** | **total population** | **Program MBSR** | **Classic follow-up and general advice** | **test** | **p** |
| Variation between T end and T start of the observation factor (FFMQ score) |  | N=35 | n=19 | n=16 | STUDENT | 0.115 |
|  | Mean (± SD) | 1.06 (± 5.13) | 2.32 (± 5.79) | -0.44 (± 3.88) |  |  |
|  | Median (Q1;Q3) | 0.00 (-2.00 ; 4.00) | 3.00 (-3.00 ; 6.00) | -1.00 (-2.00 ; 1.50) |  |  |
|  | Median (min;max) | 0.00 (-10.00 ; 13.00) | 3.00 (-10.00 ; 13.00) | -1.00 (-9.00 ; 8.00) |  |  |

##### Factor Description Experience

|  | | | **Group Name** | |  | |
| --- | --- | --- | --- | --- | --- | --- |
| **variable** | **Details** | **total population** | **Program MBSR** | **Classic follow-up and general advice** | **test** | **p** |
| Variation between Tfin and Tstart of the experience description factor (FFM score) |  | N=35 | n=20 | n=15 | WMW | 0.451 |
|  | Mean (± SD) | 0.83 (± 3.78) | 1.10 (± 3.63) | 0.47 (± 4.09) |  |  |
|  | Median (Q1;Q3) | 0.00 (-2.00 ; 4.00) | 1.00 (-2.00 ; 4.00) | -1.00 (-3.00 ; 4.00) |  |  |
|  | Median (min;max) | 0.00 (-6.00 ; 10.00) | 1.00 (-6.00 ; 7.00) | -1.00 (-4.00 ; 10.00) |  |  |

##### Factor mindfulness

|  | | | **Group Name** | |  | |
| --- | --- | --- | --- | --- | --- | --- |
| **variable** | **Details** | **total population** | **Program MBSR** | **Classic follow-up and general advice** | **test** | **p** |
| Variation between Tfin and Tstart of the mindfulness factor (FFMQ score) |  | N=33 | n=18 | n=15 | STUDENT | 0.919 |
|  | Mean (± SD) | 0.58 (± 5.51) | 0.67 (± 5.84) | 0.47 (± 5.28) |  |  |
|  | Median (Q1;Q3) | -1.00 (-3.00 ; 2.00) | -1.00 (-4.00 ; 3.00) | 0.00 (-3.00 ; 2.00) |  |  |
|  | Median (min;max) | -1.00 (-9.00 ; 16.00) | -1.00 (-8.00 ; 16.00) | 0.00 (-9.00 ; 13.00) |  |  |

##### Private event factor

|  | | | **Group Name** | |  | |
| --- | --- | --- | --- | --- | --- | --- |
| **variable** | **Details** | **total population** | **Program MBSR** | **Classic follow-up and general advice** | **test** | **p** |
| Variation between T end and T start of the private event factor (FFMQ score) |  | N=33 | n=18 | n=15 | STUDENT | 0.001 |
|  | Mean (± SD) | 2.27 (± 5.88) | 5.06 (± 6.29) | -1.07 (± 3.01) |  |  |
|  | Median (Q1;Q3) | 2.00 (-1.00 ; 5.00) | 4.50 (2.00 ; 11.00) | -1.00 (-2.00 ; 1.00) |  |  |
|  | Median (min;max) | 2.00 (-9.00 ; 16.00) | 4.50 (-9.00 ; 16.00) | -1.00 (-8.00 ; 3.00) |  |  |

##### Factor non jugemental

|  | | | **Group Name** | |  | |
| --- | --- | --- | --- | --- | --- | --- |
| **variable** | **Details** | **total population** | **Program MBSR** | **Classic follow-up and general advice** | **test** | **p** |
| Variation between Tfin and Tstart of the non judgment factor (FFMQ score) |  | N=35 | n=20 | n=15 | STUDENT | 0.525 |
|  | Mean (± SD) | 0.74 (± 6.25) | 0.15 (± 7.32) | 1.53 (± 4.56) |  |  |
|  | Median (Q1;Q3) | 1.00 (-4.00 ; 6.00) | 0.50 (-5.00 ; 4.50) | 1.00 (-1.00 ; 6.00) |  |  |
|  | Median (min;max) | 1.00 (-14.00 ; 13.00) | 0.50 (-14.00 ; 13.00) | 1.00 (-9.00 ; 8.00) |  |  |

### (V2-V1) : Variation between Tfin+3months and Tfin

#### Score pain (VAS/100)

|  | | | **Group Name** | |  | |
| --- | --- | --- | --- | --- | --- | --- |
| **variable** | **Details** | **total population** | **Program MBSR** | **Classic follow-up and general advice** | **test** | **p** |
| Variation between Tfin+3months and Tfin of the pain score felt by the patient |  | N=35 | n=20 | n=15 | STUDENT | 0.227 |
|  | Mean (± SD) | -3.31 (± 20.59) | -7.00 (± 22.03) | 1.60 (± 18.05) |  |  |
|  | Median (Q1;Q3) | -2.00 (-16.00 ; 7.00) | -4.00 (-20.00 ; 4.50) | -1.00 (-15.00 ; 14.00) |  |  |
|  | Median (min;max) | -2.00 (-65.00 ; 47.00) | -4.00 (-65.00 ; 47.00) | -1.00 (-30.00 ; 36.00) |  |  |

#### Score of disease activity (VAS/100)

##### Patient

|  | | | **Group Name** | |  | |
| --- | --- | --- | --- | --- | --- | --- |
| **variable** | **Details** | **total population** | **Program MBSR** | **Classic follow-up and general advice** | **test** | **p** |
| Change from Tfin+3months to Tfin in patient's global assessment of disease activity |  | N=35 | n=20 | n=15 | WMW | 0.934 |
|  | Mean (± SD) | -18.34 (± 97.75) | -4.10 (± 19.81) | -37.33 (± 148.32) |  |  |
|  | Median (Q1;Q3) | 1.00 (-13.00 ; 8.00) | 1.50 (-12.00 ; 6.50) | -2.00 (-13.00 ; 9.00) |  |  |
|  | Median (min;max) | 1.00 (-571.00 ; 29.00) | 1.50 (-56.00 ; 22.00) | -2.00 (-571.00 ; 29.00) |  |  |

##### Doctor

|  | | | **Group Name** | |  | |
| --- | --- | --- | --- | --- | --- | --- |
| **variable** | **Details** | **total population** | **Program MBSR** | **Classic follow-up and general advice** | **test** | **p** |
| Change from Tfin+3months to Tfin in physician's global assessment of disease activity |  | N=32 | n=19 | n=13 | STUDENT | 0.165 |
|  | Mean (± SD) | -6.88 (± 26.84) | -12.37 (± 28.50) | 1.15 (± 22.93) |  |  |
|  | Median (Q1;Q3) | -10.00 (-27.50 ; 10.00) | -20.00 (-30.00 ; 10.00) | 0.00 (-20.00 ; 10.00) |  |  |
|  | Median (min;max) | -10.00 (-50.00 ; 50.00) | -20.00 (-50.00 ; 40.00) | 0.00 (-30.00 ; 50.00) |  |  |

#### WOMAC

##### Total

|  | | | **Group Name** | |  | |
| --- | --- | --- | --- | --- | --- | --- |
| **variable** | **Details** | **total population** | **Program MBSR** | **Classic follow-up and general advice** | **test** | **p** |
| Change between Tfin+3months and Tfin in score total (Womac) |  | N=19 | n=11 | n=8 | STUDENT | 0.546 |
|  | Mean (± SD) | -8.17 (± 13.69) | -9.85 (± 15.97) | -5.86 (± 10.36) |  |  |
|  | Median (Q1;Q3) | -3.13 (-11.46 ; -1.04) | -9.38 (-12.50 ; -1.04) | -3.13 (-8.85 ; -1.04) |  |  |
|  | Median (min;max) | -3.13 (-41.67 ; 13.54) | -9.38 (-41.67 ; 13.54) | -3.13 (-28.13 ; 7.29) |  |  |

##### Pain

|  | | | **Group Name** | |  | |
| --- | --- | --- | --- | --- | --- | --- |
| **variable** | **Details** | **total population** | **Program MBSR** | **Classic follow-up and general advice** | **test** | **p** |
| Change between Tfin+3months and Tfin in score of pain (Womac) |  | N=28 | n=18 | n=10 | WMW | 0.420 |
|  | Mean (± SD) | -11.07 (± 16.06) | -12.22 (± 18.01) | -9.00 (± 12.43) |  |  |
|  | Median (Q1;Q3) | -5.00 (-25.00 ; 0.00) | -5.00 (-25.00 ; -5.00) | -5.00 (-15.00 ; 0.00) |  |  |
|  | Median (min;max) | -5.00 (-50.00 ; 25.00) | -5.00 (-50.00 ; 25.00) | -5.00 (-40.00 ; 0.00) |  |  |

##### Function

|  | | | **Group Name** | |  | |
| --- | --- | --- | --- | --- | --- | --- |
| **variable** | **Details** | **total population** | **Program MBSR** | **Classic follow-up and general advice** | **test** | **p** |
| Change between Tfin+3months and Tfin in score of function (Womac) |  | N=22 | n=12 | n=10 | STUDENT | 0.373 |
|  | Mean (± SD) | -5.55 (± 14.26) | -8.09 (± 16.43) | -2.50 (± 11.22) |  |  |
|  | Median (Q1;Q3) | -2.94 (-10.30 ; 2.94) | -5.88 (-13.24 ; 2.21) | -1.47 (-5.88 ; 2.94) |  |  |
|  | Median (min;max) | -2.94 (-45.59 ; 14.70) | -5.88 (-45.59 ; 11.76) | -1.47 (-25.00 ; 14.70) |  |  |

##### Stiffnessr

|  | | | **Group Name** | |  | |
| --- | --- | --- | --- | --- | --- | --- |
| **variable** | **Details** | **total population** | **Program MBSR** | **Classic follow-up and general advice** | **test** | **p** |
| Change between Tfin+3months and Tfin in score of stifness (Womac) |  | N=33 | n=19 | n=14 | STUDENT | 0.127 |
|  | Mean (± SD) | -9.85 (± 20.19) | -14.47 (± 21.76) | -3.57 (± 16.57) |  |  |
|  | Median (Q1;Q3) | -12.50 (-25.00 ; 12.50) | -12.50 (-37.50 ; 0.00) | -6.25 (-12.50 ; 12.50) |  |  |
|  | Median (min;max) | -12.50 (-50.00 ; 25.00) | -12.50 (-50.00 ; 25.00) | -6.25 (-25.00 ; 25.00) |  |  |

#### Reponse OARSI-OMERACT

|  | | | **Group Name** | |  | |
| --- | --- | --- | --- | --- | --- | --- |
| **variable** | **Details** | **total population** | **Program MBSR** | **Classic follow-up and general advice** | **Test** | **p** |
| OMERACT-OARSI responders between Tfin+3 months and Tfin (n(%col)) |  | N=17 | n=10 | n=7 | FISHER | 1.000 |
|  | No | 7 (41.18) | 4 (40.00) | 3 (42.86) |  |  |
|  | Yesi | 10 (58.82) | 6 (60.00) | 4 (57.14) |  |  |

#### Score SF-36

##### Score global physic

|  | | | **Group Name** | |  | |
| --- | --- | --- | --- | --- | --- | --- |
| **variable** | **Details** | **total population** | **Program MBSR** | **Classic follow-up and general advice** | **test** | **p** |
| Change between Tfin+3months and Tfin in the aggregate physical PCS score (SF-36 score) |  | N=33 | n=20 | n=13 | STUDENT | 0.586 |
|  | Mean (± SD) | 1.50 (± 6.29) | 1.99 (± 6.20) | 0.74 (± 6.61) |  |  |
|  | Median (Q1;Q3) | 1.21 (-2.54 ; 4.14) | 0.64 (-2.06 ; 4.19) | 1.52 (-4.45 ; 3.34) |  |  |
|  | Median (min;max) | 1.21 (-11.01 ; 15.09) | 0.64 (-11.01 ; 15.09) | 1.52 (-10.99 ; 14.33) |  |  |

##### Score global physic

|  | | | **Group Name** | |  | |
| --- | --- | --- | --- | --- | --- | --- |
| **variable** | **Details** | **total population** | **Program MBSR** | **Classic follow-up and general advice** | **test** | **p** |
| Variation between Tfin+3months and Tfin of the MCS aggregate psychic score (SF-36 score) |  | N=33 | n=20 | n=13 | STUDENT | 0.807 |
|  | Mean (± SD) | -0.41 (± 7.87) | -0.68 (± 6.39) | 0.02 (± 10.01) |  |  |
|  | Median (Q1;Q3) | -0.07 (-6.75 ; 3.99) | -0.36 (-5.72 ; 2.67) | 0.76 (-7.51 ; 6.66) |  |  |
|  | Median (min;max) | -0.07 (-16.80 ; 21.24) | -0.36 (-14.26 ; 11.27) | 0.76 (-16.80 ; 21.24) |  |  |

##### General health question

|  | | | **Group Name** | |  | |
| --- | --- | --- | --- | --- | --- | --- |
| **variable** | **Details** | **total population** | **Program MBSR** | **Classic follow-up and general advice** | **test** | **p** |
| Change between Tfin+3months and Tfin in perceived health (GH) (SF-36 score) |  | N=34 | n=20 | n=14 | STUDENT | 0.343 |
|  | Mean (± SD) | 2.50 (± 12.30) | 0.80 (± 12.92) | 4.93 (± 11.38) |  |  |
|  | Median (Q1;Q3) | 4.00 (-5.00 ; 8.00) | 0.00 (-10.00 ; 10.00) | 5.00 (0.00 ; 7.00) |  |  |
|  | Median (min;max) | 4.00 (-22.00 ; 32.00) | 0.00 (-22.00 ; 25.00) | 5.00 (-15.00 ; 32.00) |  |  |

#### Score HAD

##### Score anxiety

|  | | | **Group Name** | |  | |
| --- | --- | --- | --- | --- | --- | --- |
| **variable** | **Details** | **total population** | **Program MBSR** | **Classic follow-up and general advice** | **test** | **p** |
| Change between Tfin+3months and Tfin in score HAD anxiety |  | N=34 | n=20 | n=14 | WMW | 0.859 |
|  | Mean (± SD) | 0.65 (± 3.06) | 1.00 (± 3.39) | 0.14 (± 2.57) |  |  |
|  | Median (Q1;Q3) | 0.00 (-1.00 ; 1.00) | 0.00 (-1.00 ; 1.00) | 0.00 (-1.00 ; 2.00) |  |  |
|  | Median (min;max) | 0.00 (-6.00 ; 11.00) | 0.00 (-3.00 ; 11.00) | 0.00 (-6.00 ; 4.00) |  |  |

##### Score depression

|  | | | **Group Name** | |  | |
| --- | --- | --- | --- | --- | --- | --- |
| **variable** | **Details** | **total population** | **Program MBSR** | **Classic follow-up and general advice** | **test** | **p** |
| Change between Tfin+3months and Tfin in score HAD depression |  | N=34 | n=20 | n=14 | STUDENT | 0.817 |
|  | Mean (± SD) | 0.09 (± 2.04) | 0.15 (± 2.50) | 0.00 (± 1.18) |  |  |
|  | Median (Q1;Q3) | 0.00 (-1.00 ; 1.00) | 0.00 (-1.50 ; 1.00) | 0.00 (-1.00 ; 1.00) |  |  |
|  | Median (min;max) | 0.00 (-4.00 ; 6.00) | 0.00 (-4.00 ; 6.00) | 0.00 (-2.00 ; 2.00) |  |  |

#### Five Facts Mindfulness Questionnaire (FFMQ)

##### Score total (Mindfulness)

|  | | | **Group Name** | |  | |
| --- | --- | --- | --- | --- | --- | --- |
| **variable** | **Details** | **total population** | **Program MBSR** | **Classic follow-up and general advice** | **test** | **p** |
| Change between Tfin+3months and Tfin in total FFMQ score |  | N=30 | n=18 | n=12 | STUDENT | 0.689 |
|  | Mean (± SD) | 3.43 (± 10.73) | 2.78 (± 11.54) | 4.42 (± 9.80) |  |  |
|  | Median (Q1;Q3) | 4.00 (-3.00 ; 10.00) | 4.00 (-3.00 ; 10.00) | 5.00 (-0.50 ; 10.50) |  |  |
|  | Median (min;max) | 4.00 (-24.00 ; 23.00) | 4.00 (-24.00 ; 23.00) | 5.00 (-17.00 ; 18.00) |  |  |

##### Observation Factor

|  | | | **Group Name** | |  | |
| --- | --- | --- | --- | --- | --- | --- |
| **variable** | **Details** | **total population** | **Program MBSR** | **Classic follow-up and general advice** | **test** | **p** |
| Variation between Tfin+3months and Tfin of the observation factor (FFMQ score) |  | N=35 | n=20 | n=15 | STUDENT | 0.615 |
|  | Mean (± SD) | 0.51 (± 3.52) | 0.25 (± 3.97) | 0.87 (± 2.90) |  |  |
|  | Median (Q1;Q3) | 0.00 (-2.00 ; 3.00) | 0.00 (-2.00 ; 2.00) | 2.00 (-1.00 ; 3.00) |  |  |
|  | Median (min;max) | 0.00 (-5.00 ; 10.00) | 0.00 (-5.00 ; 10.00) | 2.00 (-5.00 ; 5.00) |  |  |

##### Experience Description Factor

|  | | | **Group Name** | |  | |
| --- | --- | --- | --- | --- | --- | --- |
| **variable** | **Details** | **total population** | **Program MBSR** | **Classic follow-up and general advice** | **test** | **p** |
| Variation between Tfin+3months and Tfin of the experience description factor (FFMQ score) |  | N=34 | n=20 | n=14 | STUDENT | 0.950 |
|  | Mean (± SD) | 0.97 (± 3.17) | 1.00 (± 2.97) | 0.93 (± 3.54) |  |  |
|  | Median (Q1;Q3) | 1.00 (-1.00 ; 3.00) | 1.00 (-0.50 ; 2.50) | 0.00 (-2.00 ; 3.00) |  |  |
|  | Median (min;max) | 1.00 (-4.00 ; 8.00) | 1.00 (-4.00 ; 8.00) | 0.00 (-4.00 ; 7.00) |  |  |

##### the Mindfulness Factor

|  | | | **Group Name** | |  | |
| --- | --- | --- | --- | --- | --- | --- |
| **variable** | **Details** | **total population** | **Program MBSR** | **Classic follow-up and general advice** | **test** | **p** |
| Variation between Tfin+3months and Tfin of the mindfulness factor (FFMQ score) |  | N=33 | n=19 | n=14 | WMW | 0.728 |
|  | Mean (± SD) | 1.33 (± 4.50) | 1.16 (± 4.62) | 1.57 (± 4.48) |  |  |
|  | Median (Q1;Q3) | 1.00 (0.00 ; 4.00) | 1.00 (0.00 ; 4.00) | 2.00 (-1.00 ; 4.00) |  |  |
|  | Median (min;max) | 1.00 (-14.00 ; 11.00) | 1.00 (-14.00 ; 8.00) | 2.00 (-7.00 ; 11.00) |  |  |

##### Private event factor

|  | | | **Group Name** | |  | |
| --- | --- | --- | --- | --- | --- | --- |
| **variable** | **Details** | **total population** | **Program MBSR** | **Classic follow-up and general advice** | **test** | **p** |
| Variation between Tfin+3months and Tfin of the private event factor (FFMQ score) |  | N=33 | n=19 | n=14 | STUDENT | 0.044 |
|  | Mean (± SD) | 0.09 (± 4.19) | -1.16 (± 4.00) | 1.79 (± 3.96) |  |  |
|  | Median (Q1;Q3) | 0.00 (-2.00 ; 2.00) | -2.00 (-3.00 ; 1.00) | 0.50 (0.00 ; 4.00) |  |  |
|  | Median (min;max) | 0.00 (-12.00 ; 9.00) | -2.00 (-12.00 ; 6.00) | 0.50 (-6.00 ; 9.00) |  |  |

##### Non-judgement facotr

|  | | | **Group Name** | |  | |
| --- | --- | --- | --- | --- | --- | --- |
| **variable** | **Details** | **total population** | **Program MBSR** | **Classic follow-up and general advice** | **test** | **p** |
| Variation between Tfin+3months and Tfin of the non-judgment factor (FFMQ score) |  | N=34 | n=20 | n=14 | WMW | 0.106 |
|  | Mean (± SD) | 1.12 (± 5.04) | 2.50 (± 5.49) | -0.86 (± 3.66) |  |  |
|  | Median (Q1;Q3) | 0.00 (-2.00 ; 4.00) | 0.50 (-1.50 ; 5.50) | -1.00 (-3.00 ; 2.00) |  |  |
|  | Median (min;max) | 0.00 (-8.00 ; 16.00) | 0.50 (-4.00 ; 16.00) | -1.00 (-8.00 ; 5.00) |  |  |

### (V2-V0) : VARIATION BETWEEN TEND+3MONTHS OF THE PROGRAM AND TSTART

#### Score pain (VAs/100)

|  | | | **Group Name** | |  | |
| --- | --- | --- | --- | --- | --- | --- |
| **variable** | **Details** | **total population** | **Program MBSR** | **Classic follow-up and general advice** | **test** | **p** |
| Variation between Tfin+3months and Tstart of the pain score felt by the patient (VAS) |  | N=36 | n=20 | n=16 | STUDENT | 0.031 |
|  | Mean (± SD) | -20.56 (± 28.41) | -29.60 (± 26.58) | -9.25 (± 27.26) |  |  |
|  | Median (Q1;Q3) | -15.00 (-47.50 ; 6.00) | -37.50 (-51.00 ; -8.00) | -2.00 (-29.50 ; 10.00) |  |  |
|  | Median (min;max) | -15.00 (-74.00 ; 24.00) | -37.50 (-74.00 ; 20.00) | -2.00 (-66.00 ; 24.00) |  |  |

#### Score of disease activity (VAS/100)

##### Patient

|  | | | **Group Name** | |  | |
| --- | --- | --- | --- | --- | --- | --- |
| **variable** | **Details** | **total population** | **Program MBSR** | **Classic follow-up and general advice** | **test** | **p** |
| Change between Tfin+3months and Tstart in patient's global assessment of disease activity |  | N=36 | n=20 | n=16 | STUDENT | 0.146 |
|  | Mean (± SD) | -12.08 (± 30.37) | -18.70 (± 25.20) | -3.81 (± 34.87) |  |  |
|  | Median (Q1;Q3) | -8.50 (-37.00 ; 4.50) | -12.50 (-41.50 ; 2.50) | 0.50 (-30.50 ; 23.00) |  |  |
|  | Median (min;max) | -8.50 (-63.00 ; 72.00) | -12.50 (-57.00 ; 24.00) | 0.50 (-63.00 ; 72.00) |  |  |

##### Doctor

|  | | | **Group Name** | |  | |
| --- | --- | --- | --- | --- | --- | --- |
| **variable** | **Details** | **total population** | **Program MBSR** | **Classic follow-up and general advice** | **test** | **p** |
| Variation between Tfin+3months and Tstart of the global evaluation of the activity by the physician |  | N=34 | n=20 | n=14 | WMW | 0.027 |
|  | Mean (± SD) | -15.59 (± 18.94) | -21.75 (± 18.37) | -6.79 (± 16.60) |  |  |
|  | Median (Q1;Q3) | -20.00 (-30.00 ; 0.00) | -20.00 (-40.00 ; -5.00) | -5.00 (-20.00 ; 10.00) |  |  |
|  | Median (min;max) | -20.00 (-45.00 ; 20.00) | -20.00 (-45.00 ; 10.00) | -5.00 (-30.00 ; 20.00) |  |  |

#### WOMAC index

##### Total

|  | | | **Group Name** | |  | |
| --- | --- | --- | --- | --- | --- | --- |
| **variable** | **Details** | **total population** | **Program MBSR** | **Classic follow-up and general advice** | **test** | **p** |
| Variation between Tfin+3months and Tstart of the total score (Womac) |  | N=27 | n=14 | n=13 | STUDENT | 0.215 |
|  | Mean (± SD) | -12.92 (± 14.45) | -16.29 (± 11.94) | -9.29 (± 16.46) |  |  |
|  | Median (Q1;Q3) | -14.58 (-18.75 ; -2.08) | -17.19 (-18.75 ; -8.33) | -7.29 (-14.58 ; 2.08) |  |  |
|  | Median (min;max) | -14.58 (-40.63 ; 13.54) | -17.19 (-40.63 ; 3.13) | -7.29 (-39.58 ; 13.54) |  |  |

##### Pain

|  | | | **Group Name** | |  | |
| --- | --- | --- | --- | --- | --- | --- |
| **variable** | **Details** | **total population** | **Program MBSR** | **Classic follow-up and general advice** | **test** | **p** |
| Change between Tfin+3months and Tstart in pain score (Womac) |  | N=31 | n=17 | n=14 | STUDENT | 0.337 |
|  | Mean (± SD) | -14.03 (± 17.15) | -16.76 (± 15.20) | -10.71 (± 19.30) |  |  |
|  | Median (Q1;Q3) | -10.00 (-30.00 ; 0.00) | -15.00 (-30.00 ; -5.00) | -7.50 (-15.00 ; 0.00) |  |  |
|  | Median (min;max) | -10.00 (-55.00 ; 20.00) | -15.00 (-40.00 ; 5.00) | -7.50 (-55.00 ; 20.00) |  |  |

##### Function

|  | | | **Group Name** | |  | |
| --- | --- | --- | --- | --- | --- | --- |
| **variable** | **Details** | **total population** | **Program MBSR** | **Classic follow-up and general advice** | **test** | **p** |
| Change between Tfin+3months and Tstart in function score (Womac) |  | N=29 | n=15 | n=14 | STUDENT | 0.392 |
|  | Mean (± SD) | -11.36 (± 15.73) | -13.82 (± 16.28) | -8.72 (± 15.26) |  |  |
|  | Median (Q1;Q3) | -11.76 (-19.12 ; -1.47) | -16.17 (-23.53 ; -1.47) | -8.09 (-14.71 ; 4.41) |  |  |
|  | Median (min;max) | -11.76 (-44.12 ; 23.53) | -16.17 (-44.12 ; 23.53) | -8.09 (-35.29 ; 16.17) |  |  |

##### Stiffness

|  | | | **Group Name** | |  | |
| --- | --- | --- | --- | --- | --- | --- |
| **variable** | **Details** | **total population** | **Program MBSR** | **Classic follow-up and general advice** | **test** | **p** |
| Variation between Tfin+3months and Tstart of the stiffness score (Womac) |  | N=36 | n=20 | n=16 | STUDENT | 0.109 |
|  | Mean (± SD) | -12.50 (± 24.46) | -18.75 (± 17.44) | -4.69 (± 29.89) |  |  |
|  | Median (Q1;Q3) | -12.50 (-25.00 ; 0.00) | -25.00 (-25.00 ; -12.50) | -6.25 (-31.25 ; 18.75) |  |  |
|  | Median (min;max) | -12.50 (-50.00 ; 50.00) | -25.00 (-50.00 ; 25.00) | -6.25 (-50.00 ; 50.00) |  |  |

#### Reponse OARSI-OMERACT

|  | | | **Group Name** | |  | |
| --- | --- | --- | --- | --- | --- | --- |
| **variable** | **Details** | **total population** | **Program MBSR** | **Classic follow-up and general advice** | **Test** | **p** |
| OMERACT-OARSI responders between Tfin+3months and Tstart (n(%col)) |  | N=22 | n=14 | n=8 | FISHER | 1.000 |
|  | No | 9 (40.91) | 6 (42.86) | 3 (37.50) |  |  |
|  | Yes | 13 (59.09) | 8 (57.14) | 5 (62.50) |  |  |

#### Score SF-36

##### Score physique global

|  | | | **Group Name** | |  | |
| --- | --- | --- | --- | --- | --- | --- |
| **variable** | **Details** | **total population** | **Program MBSR** | **Classic follow-up and general advice** | **test** | **p** |
| Change between Tfin+3months and Tstart in the aggregate physical PCS score (SF-36 score) |  | N=34 | n=20 | n=14 | STUDENT | 0.855 |
|  | Mean (± SD) | 3.91 (± 7.07) | 3.72 (± 6.74) | 4.18 (± 7.77) |  |  |
|  | Median (Q1;Q3) | 4.19 (-0.35 ; 7.67) | 3.40 (-0.00 ; 7.94) | 4.19 (-1.23 ; 7.67) |  |  |
|  | Median (min;max) | 4.19 (-11.00 ; 22.61) | 3.40 (-11.00 ; 15.98) | 4.19 (-6.05 ; 22.61) |  |  |

##### Global psychic score

|  | | | **Group Name** | |  | |
| --- | --- | --- | --- | --- | --- | --- |
| **variable** | **Details** | **total population** | **Program MBSR** | **Classic follow-up and general advice** | **test** | **p** |
| Variation between Tfin+3months and Tbeginning of the MCS aggregate psychic score (SF-36 score) |  | N=34 | n=20 | n=14 | WMW | 0.903 |
|  | Mean (± SD) | 1.47 (± 10.16) | 1.90 (± 9.47) | 0.86 (± 11.42) |  |  |
|  | Median (Q1;Q3) | -0.31 (-3.05 ; 7.50) | -0.22 (-4.45 ; 7.79) | -0.31 (-3.05 ; 3.79) |  |  |
|  | Median (min;max) | -0.31 (-27.72 ; 24.44) | -0.22 (-14.84 ; 24.44) | -0.31 (-27.72 ; 18.37) |  |  |

##### Question general health

|  | | | **Group Name** | |  | |
| --- | --- | --- | --- | --- | --- | --- |
| **variable** | **Details** | **total population** | **Program MBSR** | **Classic follow-up and general advice** | **test** | **p** |
| Change between Tfin+3months and Tstart in perceived health (GH) (SF-36 score) |  | N=35 | n=20 | n=15 | STUDENT | 0.900 |
|  | Mean (± SD) | 2.46 (± 15.50) | 2.75 (± 15.80) | 2.07 (± 15.63) |  |  |
|  | Median (Q1;Q3) | 0.00 (-5.00 ; 10.00) | 1.50 (-5.00 ; 10.63) | -2.00 (-5.00 ; 10.00) |  |  |
|  | Median (min;max) | 0.00 (-28.00 ; 42.00) | 1.50 (-28.00 ; 42.00) | -2.00 (-27.00 ; 40.00) |  |  |

#### Score HAD

##### Score anxiety

|  | | | **Group Name** | |  | |
| --- | --- | --- | --- | --- | --- | --- |
| **variable** | **Details** | **total population** | **Program MBSR** | **Classic follow-up and general advice** | **test** | **p** |
| Variation between Tfin+3months and Tstart of the HAD anxiety score |  | N=36 | n=20 | n=16 | STUDENT | 0.540 |
|  | Mean (± SD) | -0.83 (± 2.86) | -1.10 (± 2.67) | -0.50 (± 3.14) |  |  |
|  | Median (Q1;Q3) | -1.00 (-3.00 ; 1.00) | -1.00 (-2.50 ; 0.50) | -1.00 (-3.00 ; 2.00) |  |  |
|  | Median (min;max) | -1.00 (-6.00 ; 5.00) | -1.00 (-6.00 ; 5.00) | -1.00 (-6.00 ; 5.00) |  |  |

##### Score depression

|  | | | **Group Name** | |  | |
| --- | --- | --- | --- | --- | --- | --- |
| **variable** | **Details** | **total population** | **Program MBSR** | **Classic follow-up and general advice** | **test** | **p** |
| Variation between Tfin+3months and Tstart of the HAD depression score |  | N=36 | n=20 | n=16 | STUDENT | 0.744 |
|  | Mean (± SD) | -0.47 (± 2.57) | -0.60 (± 2.41) | -0.31 (± 2.82) |  |  |
|  | Median (Q1;Q3) | 0.00 (-1.50 ; 1.00) | -1.00 (-1.50 ; 1.00) | 0.00 (-1.50 ; 1.00) |  |  |
|  | Median (min;max) | 0.00 (-7.00 ; 5.00) | -1.00 (-7.00 ; 4.00) | 0.00 (-6.00 ; 5.00) |  |  |

#### Five Facts Mindfulness Questionnaire (FFMQ)

##### Score total (Mindfulness)

|  | | | **Group Name** | |  | |
| --- | --- | --- | --- | --- | --- | --- |
| **variable** | **Details** | **total population** | **Program MBSR** | **Classic follow-up and general advice** | **test** | **p** |
| Variation entre Tfin+3mois et Tdébut du score FFMQ total |  | N=32 | n=18 | n=14 | STUDENT | 0.072 |
|  | Mean (± SD) | 9.06 (± 14.29) | 13.06 (± 15.09) | 3.93 (± 11.76) |  |  |
|  | Median (Q1;Q3) | 10.00 (-1.50 ; 17.50) | 13.50 (3.00 ; 18.00) | 4.50 (-7.00 ; 10.00) |  |  |
|  | Median (min;max) | 10.00 (-16.00 ; 43.00) | 13.50 (-13.00 ; 43.00) | 4.50 (-16.00 ; 27.00) |  |  |

##### Factor observation

|  | | | **Group Name** | |  | |
| --- | --- | --- | --- | --- | --- | --- |
| **variable** | **Details** | **total population** | **Program MBSR** | **Classic follow-up and general advice** | **test** | **p** |
| Variation between Tfin+3months and Tstart of the observation factor (FFMQ score) |  | N=35 | n=19 | n=16 | STUDENT | 0.054 |
|  | Mean (± SD) | 1.60 (± 3.65) | 2.68 (± 3.71) | 0.31 (± 3.22) |  |  |
|  | Median (Q1;Q3) | 2.00 (-1.00 ; 4.00) | 3.00 (0.00 ; 5.00) | -0.50 (-2.00 ; 3.50) |  |  |
|  | Median (min;max) | 2.00 (-6.00 ; 10.00) | 3.00 (-5.00 ; 10.00) | -0.50 (-6.00 ; 5.00) |  |  |

##### Factor Description Experience

|  | | | **Group Name** | |  | |
| --- | --- | --- | --- | --- | --- | --- |
| **variable** | **Details** | **total population** | **Program MBSR** | **Classic follow-up and general advice** | **test** | **p** |
| Variation between Tfin+3months and Tstart of the experience description factor (FFMQ score) |  | N=36 | n=20 | n=16 | STUDENT | 0.190 |
|  | Mean (± SD) | 1.31 (± 4.03) | 2.10 (± 3.91) | 0.31 (± 4.08) |  |  |
|  | Median (Q1;Q3) | 2.00 (-2.00 ; 4.50) | 2.00 (-0.50 ; 6.00) | 0.50 (-2.50 ; 4.00) |  |  |
|  | Median (min;max) | 2.00 (-8.00 ; 9.00) | 2.00 (-4.00 ; 9.00) | 0.50 (-8.00 ; 5.00) |  |  |

##### Mindfulness factor

|  | | | **Group Name** | |  | |
| --- | --- | --- | --- | --- | --- | --- |
| **variable** | **Details** | **total population** | **Program MBSR** | **Classic follow-up and general advice** | **test** | **p** |
| Variation between Tfin+3months and Tstart of the mindfulness factor (FFMQ score) |  | N=33 | n=19 | n=14 | STUDENT | 0.991 |
|  | Mean (± SD) | 1.94 (± 4.70) | 1.95 (± 4.01) | 1.93 (± 5.66) |  |  |
|  | Median (Q1;Q3) | 1.00 (0.00 ; 6.00) | 2.00 (0.00 ; 6.00) | 0.00 (-2.00 ; 6.00) |  |  |
|  | Median (min;max) | 1.00 (-5.00 ; 16.00) | 2.00 (-4.00 ; 9.00) | 0.00 (-5.00 ; 16.00) |  |  |

##### Private event factor

|  | | | **Group Name** | |  | |
| --- | --- | --- | --- | --- | --- | --- |
| **variable** | **Details** | **total population** | **Program MBSR** | **Classic follow-up and general advice** | **test** | **p** |
| Variation between Tfin+3months and Tstart of the private event factor (FFMQ score) |  | N=35 | n=19 | n=16 | STUDENT | 0.003 |
|  | Moyenne (± ET) | 2.29 (± 3.89) | 4.00 (± 4.07) | 0.25 (± 2.52) |  |  |
|  | Médiane (Q1;Q3) | 2.00 (-1.00 ; 4.00) | 4.00 (1.00 ; 8.00) | 0.50 (-1.50 ; 2.00) |  |  |
|  | Médiane (min;max) | 2.00 (-4.00 ; 10.00) | 4.00 (-3.00 ; 10.00) | 0.50 (-4.00 ; 6.00) |  |  |

##### Facteur non jugement

|  | | | **Group Name** | |  | |
| --- | --- | --- | --- | --- | --- | --- |
| **variable** | **Details** | **total population** | **Program MBSR** | **Classic follow-up and general advice** | **test** | **p** |
| Variation between Tfin+3months and Tstart of the non judgment factor (FFMQ score) |  | N=36 | n=20 | n=16 | STUDENT | 0.293 |
|  | Mean (± SD) | 1.86 (± 4.96) | 2.65 (± 5.76) | 0.88 (± 3.69) |  |  |
|  | Median (Q1;Q3) | 1.00 (-0.50 ; 4.00) | 2.00 (0.00 ; 5.50) | 1.00 (-1.00 ; 3.00) |  |  |
|  | Median (min;max) | 1.00 (-9.00 ; 12.00) | 2.00 (-9.00 ; 12.00) | 1.00 (-7.00 ; 7.00) |  |  |
