# Supplementary material for: Evaluation of mindfulness based stress reduction in symptomatic knee or hip osteoarthritis patients: a pilot randomized controlled trial
Source: BMC Rheumatol. 2022 May 30;6:46. doi: 10.1186/s41927-022-00277-9 (PMC9150306; doi:10.1186/s41927-022-00277-9)
Supplement: Supplementary file 2 — Additional file 2. Analysis of assiduousness to the MBSR workshops. [file 41927_2022_277_MOESM2_ESM.docx]

**Supplementary files:**

**Supplementary Table S1:**

Painkillers consumption analysis

| Current medical use | | MBSR group | Usual care group | |
| --- | --- | --- | --- | --- |
|  |  | n/N (%) | n/N (%) | |
| Non-steroidal anti-inflammatory drug | baseline | 6/20 (30) | 6/20 (30) |  |
|  | V1 | 5/18 (28) | 7/14 (50) |  |
|  | V2 | 5/20 (25) | 5/15 (33) |  |
| Analgesic grade 1 | baseline | 0/20 | 6/20 (30) |  |
|  | V1 | 8/20 (40) | 7/20 (35) |  |
|  | V2 | 9/20 (45) | 6/20 (30) |  |
| Analgesic grade 2 | baseline | 2/20 (10) | 0/20 |  |
|  | V1 | 2/20 (10) | 3/20 (15) |  |
|  | V2 | 2/20 (10) | 1/20 (5) |  |

V1, the end of the MBSR program; V2, the final follow-up (6 months).

**Supplementary Table S2:**

Analysis of assiduousness to the MBSR workshops in the MBSR group

| MBSR sessions assiduousness | n/N |
| --- | --- |
| $\geq$6 sessions | 18/20 |
| $<$ 4/8 sessions | 2/20 |
| MBSR home training assiduousness (V1) | n/N |
| 7/7 days full duration | 5/19 |
| 7/7 days variable duration | 5/19 |
| 4-5 /week | 6/19 |
| 2-3 /week | 2/19 |
| 0-1 /week | 1/19 |
| MBSR home training assiduousness (V2) | n/N |
| 7/7 days full duration | 1/20 |
| 7/7 days variable duration | 2/20 |
| 4-5 /week | 4/20 |
| 2-3 /week | 7/20 |
| 0-1 /week | 6/20 |

V1, the end of the MBSR program; V2, the final follow-up (6 months).


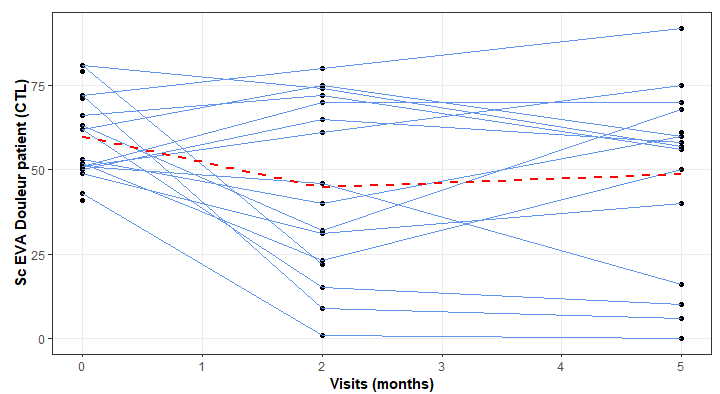


**Supplementary Fig. S1:** Analysis of pain VAS variation between baseline, V1 and V2 in the usual care group. V1, the end of the MBSR program; V2, the final follow-up (6 months).


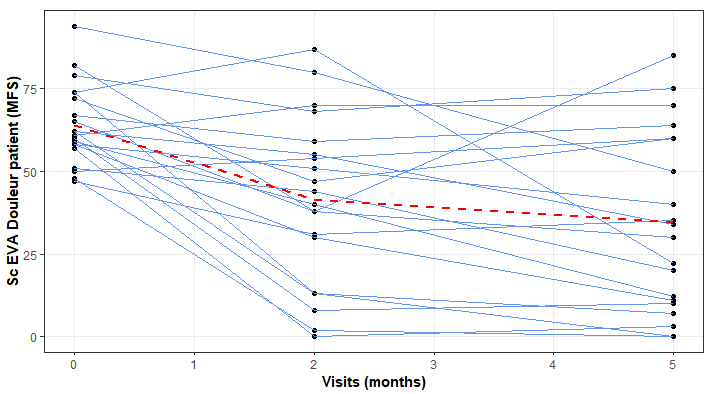


**Supplementary Fig. S2:** Analysis of pain VAS variation between baseline, V1 and V2 in the MBSR group. V1, the end of the MBSR program; V2, the final follow-up (6 months).
